# Total Flavonoids of Chuju Decrease Oxidative Stress and Cell Apoptosis in Ischemic Stroke Rats: Network and Experimental Analyses

**DOI:** 10.3389/fnins.2021.772401

**Published:** 2021-12-09

**Authors:** Cong Wang, Hao Chen, Hui-hui Jiang, Bin-bin Mao, Hao Yu

**Affiliations:** ^1^College of Life and Health Sciences, Anhui Science and Technology University, Chuzhou, China; ^2^School of Pharmacy, Anhui University of Chinese Medicine, Hefei, China; ^3^School of Chinese Medicine, Bozhou University, Bozhou, China; ^4^Department of Pharmacy, College of Life and Health Sciences, Anhui Science and Technology University, Chuzhou, China

**Keywords:** total flavonoids of Chuju, network pharmacology, ischemic stroke, MCAO, oxidative stress, apoptosis, PI3K/Akt/mTOR

## Abstract

**Background:** Pharmacological research results showed that total flavonoids of Chuju (TFCJ) could be used to treat acute myocardial ischemia and myocardial ischemia-reperfusion injury. In this study, we explored the protective effect of TFCJ on ischemic stroke (IS) in the IS rat model. We hypothesized that TFCJ might exert its neuroprotective effects by suppressing apoptosis and oxidative stress that are closely related to PI3K/Akt/mTOR signaling pathway.

**Method:** TFCJ (10, 20, and 40 mg/kg) was administered for 7 days. Rats (260 ± 20 g) were subjected to middle cerebral artery occlusion (MCAO) for 2 h and reperfusion for 24 h. The neuroprotective effect of TFCJ was substantiated in terms of neurological deficits, oxidative stress (superoxide dismutase, glutathione peroxidase, catalase, and malondialdehyde), pathomorphological changes (HE staining and TUNEL staining), and neurobehavioral functions in the rats. Then, we employed network pharmacology to reveal the potential mechanism of TFCJ against IS. Western blot was used to determine the levels of PI3K/AKT/mTOR pathway proteins. The expression of BCL-2, BAX, and cleaved-Caspase-3 was also measured by Western blots and RT-PCR.

**Results:** The histopathological assessment showed that TFCJ reduced MCAO-induced brain damage. Besides, TFCJ exerted a protective role in MCAO rats by alleviating cell apoptosis and oxidative stress. Network pharmacology showed that TFCJ might be used against IS through the PI3K/AKT signaling pathway. TFCJ reduced cell apoptosis and oxidative stress by increasing the level of p-AKT and p-mTOR in MCAO rats, while the effect of TFCJ was significantly reversed when applying LY294002 (PI3k inhibitor).

**Conclusion:** These results indicated that TFCJ might decrease oxidative stress and apoptosis that are closely related to PI3K/Akt/mTOR pathway in IS. TFCJ is a promising authentic traditional Chinese medicine for the management of IS.

## Introduction

Stroke is a common disease characterized by a decreased or blockage in the brain’s blood supply, which accounted for 11.59% of all deaths in 2019 (GBD 2019 Demographics Collaborators, 2020). In the United States, every year more than 795,000 people suffer a stroke, 610,000 of these being first or new cases (GBD 2019 Demographics Collaborators, 2020). Stroke can be categorized into ischaemic stroke (IS), cerebral hemorrhage, and subarachnoid hemorrhage (Garbuzova-Davis et al., 2016). In the United States, about 87% of all strokes are ischemic strokes (GBD 2019 Demographics Collaborators, 2020). IS mainly occurs due to the middle cerebral artery occlusion (MCAO), causing regional ischemia and hypoxia in brain tissue, leading to DNA damage and cell apoptosis (Li et al., 2018). Currently, there are no specific drugs for the clinical treatment of IS. While existing drugs can protect against free radical damage, thrombolytic drugs, antiplatelet aggregation drugs, anti-inflammatory drugs, and anticoagulants (Carvalhal et al., 2019; Kanazawa et al., 2019; Liu et al., 2019), they have also been associated with serious side effects and have a single mechanism of action (Allegaert et al., 2010; Ma et al., 2016; Polderman et al., 2018), which limits their clinical application (Ren et al., 2017).

Traditional Chinese medicines and their derived compounds have been increasingly used to treat ischemic cerebrovascular diseases. For instance, Xuesaitong (Shi et al., 2017; Yang et al., 2018), as a positive drug, is a commonly used drug for cerebrovascular diseases in clinic. Having multi-component, multi-pathway, and multi-target impact (Gao et al., 2015; Zhu et al., 2021), these medicines can provide new alternative strategies for the treatment of IS in China. Chuju is an authentic medicinal material produced in China, whose main active ingredients are total flavonoids of Chuju (TFCJ). TFCJ has been reported to have anti-myocardial ischemia (Nian et al., 2019), hypoglycemic (Yu et al., 2014), analgesic (Zhang, 2013), anti-inflammatory (Miao et al., 2012), anti-blood stasis (Yu et al., 2012a), and anti-oxidant pharmacological properties (Yu et al., 2012b). Pharmacological research results showed that TFCJ could resist acute myocardial ischemia and myocardial ischemiareperfusion injury, and the mechanism might be associated with anti-lipid peroxidation and inhibition of intracellular Ca^2 +^overload (Yu et al., 2012b).

There are few studies on the pharmacological effects of TFCJ, while the protective effects of TFCJ against apoptosis and oxidative stress in cerebral ischemia-reperfusion injury have not yet been reported. Bu et al. (2019) reported that acacetin, 5,7-dihydroxy-4′-methoxyflavone associated with inhibition of microglia-mediated inflammation and the NLRP3 signaling pathway, downregulates the protein expression of Toll-like receptor 4, nuclear factor kappa B, NLRP3, procaspase-1, caspase-1, pro-interleukin-1β, and interleukin-1β in the MCAO mice. Moreover, Pang et al. (2018) reported that apigenin, a low-toxicity and non-mutagenic flavone subclass of flavonoid, affects caveolin-1, VEGF, Bcl-2, cleaved-Caspase-3, Beclin-1, and mTOR expression, and promotes cell proliferation *in vivo* and *in vitro*, tube formation, and cell migration while inhibiting apoptosis and autophagy. In addition, Liu S. et al. (2020) reported that luteolin (3,4,5,7-tetrahydroxyflavone) upregulates SIRT3-targeted and p-mTOR expression, and downregulates p-AMPK expression *in vivo*. Also, Wang Y. Y. et al. (2020) reported that quercetin, a flavonoid present in many fruits and vegetables, could upregulate p-ERK and p-Akt expression *in vivo* and *in vitro*, which may have an important role against IS.

The MCAO involves two processes of ischemia and reperfusion, and the effects of the processes on the body at different periods are also different. In the early stage of reperfusion, the accumulation of free radicals, the cascade of reactions, and the calcium overload in nerve cells are the main causes of cerebral ischemic injury (Nishimura et al., 2016; Di Costanzo et al., 2020). Oxidative stress is the main reason for the aggravation of brain injury (Cornelius et al., 2013; Wang H. et al., 2020). After ischemia-reperfusion, the production of reactive oxygen species in cells is rapidly induced, leading to an imbalance in the ratio of oxidative factors and antioxidant factors in the brain.

PI3K/Akt/mTOR pathway is a core pathway involved in the occurrence and development of IS that has a regulatory effect on oxidative stress and apoptosis (Asati et al., 2016; Maiti et al., 2019). PI3K/Akt/mTOR signaling pathway is an important intracellular signal pathway (Chen et al., 2020) and one of the key signal pathways for neuroprotection. It is mainly involved in neuron proliferation, differentiation, cell metabolism, programmed apoptosis, and oxidative damage. As an intracellular phosphatidylinositol kinase (Chen et al., 2020; Qiao et al., 2021), PI3K regulates the activation or inhibition of downstream pathways in IS to inhibit apoptosis and oxidative stress. As one of the key downstream pathways (Chen et al., 2020; Wang et al), AKT also has an important role in cell survival and apoptosis. Downstream targets, threonine (Thr308), and serine (Ser473) sites on Akt protein are phosphorylated and activated to activate downstream mTOR (Zhang G. et al., 2020). Increased mTOR activity can reduce autophagy and restore the complete complement of lysosomes in cells (Zhang T. et al., 2020). Therefore, the activation of PI3K/Akt/mTOR can promote neuroprotection after central nervous system injury, thus promoting the recovery of the central nervous system by maintaining nerve metabolism. The phosphorylation of Akt and mTOR has a cytoprotective role in cell protection through PI3K/Akt/mTOR signaling pathway (Miao et al., 2020; Zhang T. et al., 2020). Many studies have demonstrated the critical role of the PI3K/Akt/mTOR signal pathway in alleviating cerebral ischemic injury (Tian et al., 2019; Zhang H. et al., 2020). However, the role of the PI3KAkt-mTOR signal pathway in TFCJ’s neuroprotection against IS has been rarely explored.

In this study, we explored the protective effect of TFCJ on IS in rats. We hypothesized that TFCJ might exert its neuroprotective effects by suppressing apoptosis and oxidative stress via activation of the PI3K/Akt/mTOR signaling pathway.

## Materials and Methods

### Drugs

Air-dried TFCJ was treated twice with 70% aqueous ethanol (1:25, w/v), mixed and sonicated 60°C for 40 min, then concentrated in a rotary evaporato as described by the previous method (Liu S. et al., 2020; Ma et al., 2021). The crude extract was purified by the D-101 macroporous resin. TFCJ (60.21% content) was measured by UV-vis-spectrophotometer, and rutin was used as a reference compound. TFCJ and Xuesaitong (5 mg/pill, KPC Xuesaitong Pharmaceutical Co., Ltd.) were dissolved in 0.5% Carboxymethylcellulose sodium (CMC) before use.

### Animals

Experiments were conducted on 7-weeks old-male Sprague-Dawley rats (240–280 g body weight). All the animals were purchased from Huaxing Experimental Animal Farm (Zhengzhou, China), and maintained in standard housing conditions in colony cages 12 h light/dark cycles with free access to food and water. The animal care and experimental procedures were approved by Animal Ethics Committee of Anhui Science and Technology University. Because estrogen has a certain effect on the experimental results, male rats were selected as experimental animals.

### Middle Cerebral Artery Occlusion Establishment

Before the operation, the rats were fasted for 12 h to reduce the mortality during and after the operation and anesthetized by intraperitoneal injection of 2% pentobarbital sodium (4 mL⋅kg^–1^). We slowly inserted the threaded plug from the left external carotid artery (ECA) through the common carotid artery (CCA) into the internal carotid artery (ICA) until the origin of middle cerebral artery (MCA) was blocked as the previous literature (Longa et al., 1989; Olga Nikolaevna et al., 2020). After ischemia 2 h, the blood supply was restored, and reperfusion was achieved. The reperfusion process continued for 24 h.

### Animal Grouping

#### Relationship Between Total Flavonoids of Chuju Anti-IS and Apoptosis and Oxidative Stress

To understand the relationship between TFCJ anti-IS and apoptosis and oxidative stress, 120 rats were randomly divided into six groups (*n* = 20/group). Sham group, MCAO group, TFCJ-H group (40 mg⋅kg^–1^ TFCJ), TFCJ-M group (20 mg⋅kg^–1^ TFCJ), TFCJ-L group (10 mg⋅kg^–1^ TFCJ), Xuesaitong group (40 mg⋅kg^–1^ Xuesaitong, equivalent dose conversion according to the body surface area of rats and humans).

#### Relationship Between Total Flavonoids of Chuju Anti-IS and PI3K/Akt/mTOR Pathway

To explore the relationship between TFCJ anti-IS and PI3K/Akt/mTOR pathway, 40 rats were randomly divided into four groups (*n* = 10/group) as follows:Sham group, MCAO group, TFCJ group (40 mg⋅kg^–1^ TFCJ), TFCJ + LY group (40 mg⋅kg^–1^ TFCJ, 300 μg⋅kg^–1^ LY294002 injection in the tail vein before reperfusion 30 min).

All rats received intragastric administration once daily for 7 days before MCAO, MCAO group and sham group received 10 mL⋅kg^–1^ 0.5% CMC. Sham-operated rats received all surgical procedures but without the suture insertion.

### Neurological Evaluation

After reperfusion for 24 h, the degree of neurological injury was performed by researchers who were blinded to the grouping (Joya et al., 2021). Evaluations were performed as follows: Grade I, no nerve injury symptoms; Grade II, unable to fully extend the left front paw; Grade III, unable to fully extend the left front paw, circle to the left when walking; Grade IV, unable to fully extend the left front paw, hemiplegia to the left when walking; Grade V, loss of consciousness, unable to walk spontaneously.

### Cerebral Infarct Area

After neurological evaluation, the rats were sacrificed, and the rat brain was sliced with 2 mm thin slices along the coronal plane. The slices were incubated with 2% TTC dye solution at 37°C for 15 min in dark. After rinsing, the slices were fixed in 4% paraformaldehyde over 24 h. Image pro plus 6.0 software (Media Cybernetics, Rockville, MD, United States) was employed for infarct area analysis. Red stained areas corresponded to unaffected areas, while that stained in white was the infarcted area (Joya et al., 2021). The cerebral infarct area percentage was: (cerebral infarct area/total brain area) × 100%, which is the percentage of the brain area affected by the infarct.

### Oxidative Stress Evaluation

The brain hemisphere ipsilateral to MCAO was homogenized with pre-cooled physiological salt, centrifuged like the blood, and the supernatant was immediately collected and stored in the refrigerator at −80°C until use. SOD, GSH-Px, CAT activities, and the content of MDA (Liao et al., 2020; Homma et al., 2021; Liu et al., 2021)were determined according to manufacturer’s instructions.

### Histology

The animals were anesthetized and decapitated, and brains were collected. The brain sample was embedded in paraffin, coronally sliced at 5-μm, and measured by HE staining (Zhang L. et al., 2021), and terminal deoxynucleotidyl transferase (TdT)-mediated dUTP nick-end labeling (TUNEL) staining (Lee et al., 2021). The areas around five randomly selected injury sites were quantified. Images were acquired by an Olympus CX41 microscope and analyzed by Image-Pro Plus.

### Prediction of the Targets of Total Flavonoids of Chuju

The chemical compositions of TFCJ were identified from Traditional Chinese Medicine Systems Pharmacology database and analysis platform (TCMSP^1^) (Ru et al., 2014), Integrative Pharmacology-Based Research Platform of Traditional Chinese Medicine (TCMIP^2^) (Xu et al., 2019), and Bioinformatics Analysis Tool for Molecular Mechanism of Traditional Chinese Medicine (BATMAN-TCM^3^) (Liu et al., 2016). The compound targets were downloaded from PharmMapper database^4^ (Wang et al., 2017).

### Prediction of IS-Related Targets

The key words ‘‘brain infarction,’’ ‘‘cerebral ischemia,’’ ‘‘cerebral infarction,’’ and ‘‘stroke’’ were input into the following GeneCards database^5^, OMIM database^6^ (Amberger et al., 2015), and DisGeNET database^7^ (Piñero et al., 2020) to search for IS-related targets.

### Network Construction and Analysis

The common TFCJ and IS-related targets were uploaded to the String11.0 database^8^ (Szklarczyk et al., 2019), the type was set to “Homo sapiens,” the confidence level was set to >0.700. Protein–protein interactions (PPI) network and herb-compound-target-disease network were constructed by Cytoscape 3.7.2(Shannon et al., 2003).

### Gene Set Enrichment Analysis

The common TFCJ and IS targets were uploaded to the DAVID 6.8 databases^9^ (Huang da et al., 2009) that used to analyze the GO and KEGG pathways enrichment of common targets. In our work, GO and KEGG terms with *P* < 0.01 were considered as significantly enrichment analyses.

### Oxidative Stress Evaluation

The brain hemisphere ipsilateral to MCAO was homogenized with pre-cooled physiological salt, and the supernatant was collected and stored in the refrigerator at −80°C until use. SOD, GSH-Px, CAT activities, and the content of MDA were determined according to manufacturer’s instructions.

### Real-Time Quantitative PCR

Total RNA of the ischemic penumbra of brain tissue was extracted using RNA isolater Total RNA Extraction Reagent (R401-01, Vazyme, Nanjing, China). The isolated RNA samples were reverse transcribed using HiScript^®^ QRT SuperMix for qPCR (R122-01, Vazyme, Nanjing, China) following standard techniques.

Data of BCL-2, BAX, and cleaved-Caspase-3 mRNA were normalized to β-actin mRNA (Table 1). RT-PCR was performed using a quantitative ChamQ Universal SYBR qPCR Master Mix (Q711-02, Vazyme, Nanjing, China).

**TABLE 1:** Primers for real-time quantitative PCR (RT-PCR) in this study.

Gene	Primer names	Primer sequences
BCL-2	Primer_F	CTTCTCTCGTCGCTACCGTC
	Primer_R	CAATCCTCCCCCAGTTCACC
BAX	Primer_F	GAACCATCATGGGCTGGACA
	Primer_R	GTGAGTGAGGCAGTGAGGAC
Cleaved-Caspase-3	Primer_F	CGGACCTGTGGACCTGAAAA
	Primer_R	AGTCAGACTCCGGCAGTAGT
β-actin	Primer_F	CCCATCTATGAGGGTTACGC
	Primer_R	TTTAATGTCACGCACGATTT

### Western Blot

We performed Western blot analysis following previously described protocols (Abbruzzese et al., 2020). The total proteins in the cerebral ischemia-reperfusion brain were purified, centrifuged, and collected in the supernatant. The protein concentrations were quantified with a bicinchoninic acid (BCA) protein assay kit (P0010, Beyotime, Shanghai, China). The protein samples were first loaded into 10% sodium dodecyl sulfate-polyacrylamide gel electrophoresis (SDS-PAGE) and then transferred to polyvinylidene fluoride (PVDF) membranes. After blocking with 5% fat-free milk or BSA for 1 h, the PVDF membranes were incubated with primary antibodies at 4°C overnight (Table 2), following incubation with HRP-conjugated goat anti-mouse secondary polyclonal antibody (1:5000, 66009-1-Ig, Proteintech, Wuhan, China) for 2 h at room temperature. The protein bands were visualized using enhanced chemiluminescence (ECL), β-actin served as a loading control, and densitometric analysis was determined by ImageJ software. The ratio of the OD of the target protein to β-actin was used to represent the relative expression levels of the target proteins.

**TABLE 2:** Primary antibodies for Western blot in this study.

Primary antibodies	Dilution	Company	Catalog
Anti-AKT	1:1000	Proteintech	10176-2-AP
Anti-mTOR	1:3000	Proteintech	20657-1-AP
Anti-phospho-AKT (Ser473)	1:2000	Proteintech	66444-1-Ig
Anti-phospho-mTOR (Ser2448)	1:2000	Proteintech	67778-1-Ig
Anti-BCL-2	1:1000	Proteintech	12789-1-AP
Anti-BAX	1:3000	Proteintech	50599-2-Ig
Anti-cleaved-caspase-3	1:1000	CST	#9661
Anti-β-actin	1:5000	Proteintech	66009-1-Ig
HRP-conjugated goat anti-mouse secondary polyclonal antibody	1:5000	Proteintech	66009-1-Ig

### Statistical Analysis

All data were expressed as mean ± SD; SPSS 25.0 statistical software (IBM SPSS Statistics for Windows, IBM Corp.) was adopted for all statistical analyses. The multiple variables were performed by one-way analysis of variance (ANOVA), and student’s *t*-tests were used for comparison of variable pairs. *P* < 0.05 was considered as statistically significant.

## Results

### Total Flavonoids of Chuju Alleviates Nerve Injury

**FIGURE 1:**
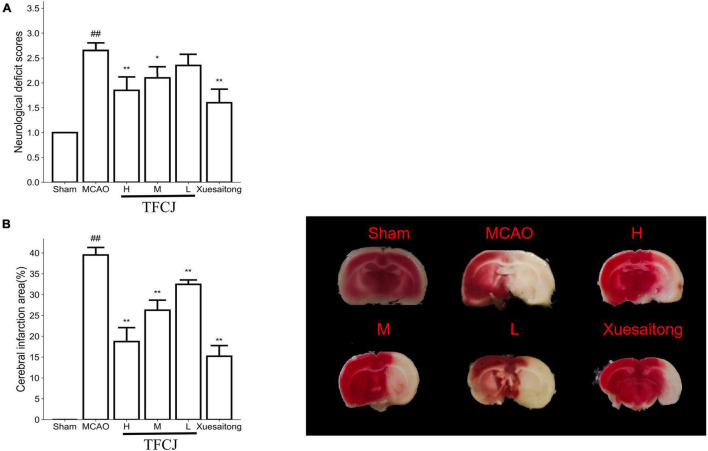
The protective effects of total flavonoids of Chuju (TFCJ) on middle cerebral artery occlusion (MCAO) inducing brain injury. **(A)** Effects of TFCJ on the neurofunctional score. **(B)** Effects of TFCJ on the infarction size. Results were shown as mean ± SD (*n* = 10, n, numbers of rats), ^#^*P* < 0.05; ^##,**^P < 0.01.

The neurological deficit scores and cerebral infarction area were evaluated (Figure 1) after reperfusion for 24 h. The neurological deficit scores in the MCAO group were higher than that in the sham group (Figure 1A, ^##^*P* < 0.01); the neurological deficits in the rats pre-treated with TFCJ (10, 20, and 40 mg⋅kg^–1^) were significantly lower than those in the MCAO group (see Figure 1A, *P < 0.05, ^**^P < 0.01); the neurological deficit scores in the Xuesaitong group significantly decreased compared to the MCAO group (Figure 1B, ^**^P < 0.01).

The sham group had a smaller cerebral infarct area, while the MCAO group had a bigger infarct area (Figure 1B, ^##^*P* < 0.01). Cerebral infarct area in three dose groups of TFCJ was much lower than MCAO group (Figure 1B, ^**^P < 0.01). The cerebral infarct area in the Xuesaitong group was significantly decreased than the MCAO group (Figure 1B, ^**^P < 0.01).

### Total Flavonoids of Chuju Reduces Oxidative Stress in Middle Cerebral Artery Occlusion Rats

**FIGURE 2:**
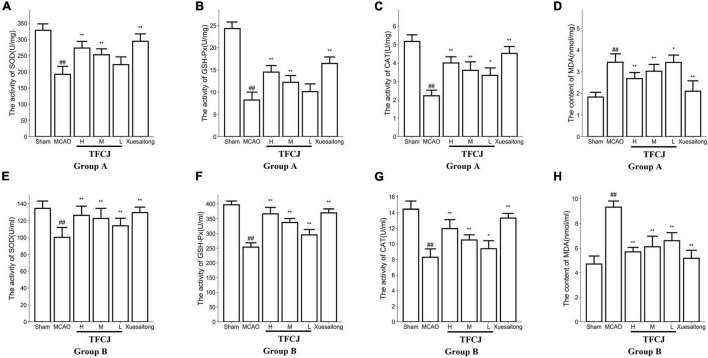
Effects of TFCJ on MCAO inducing oxidative stress. **(A)** SOD activity (Group A: brain tissue); **(B)** GSH-Px activity (Group A: brain tissue); **(C)** CAT activity (Group A: brain tissue); **(D)** the MDA level (Group A: brain tissue); **(E)** SOD activity (Group B: serum); **(F)** GSH-Px activity (Group B: serum); **(G)** CAT activity (Group B: serum); **(H)** MDA activity (Group B: serum). Results were showed as mean ± SD (*n* = 10), ^#^*P* < 0.05; ^##,**^P < 0.01.

The activity of SOD, GSH-Px, and CAT in the brain tissue and serum were lower in the MCAO group than in the sham group (Figures 2A–C,E–G, ^##^*P* < 0.01), and the concentration of MDA was higher in the MCAO group than in sham group (Figures 2D,H, ^##^*P* < 0.01), indicating that MCAO induced oxidative stress in the brain tissue and serum.

The activity of SOD, GSH-Px, and CAT in the brain tissue and serum of high and middle dose groups of TFCJ was much greater than the MCAO group (Figures 2A–C,E–G, ^**^P < 0.01). The concentration of MDA in the brain tissue and serum of the three-dose groups of TFCJ was significantly lower compared to the MCAO group (Figures 2D,H, *P < 0.05, ^**^P < 0.01).

Compared with the MCAO group, the activity of SOD and GSH-Px in the serum of the TFCJ-L group was significantly improved (Figures 2E,F, ^**^P < 0.01), and the activity of CAT in the serum and brain tissue was significantly increased (Figures 2C,G, *P < 0.05).

Compared with the MCAO group, the activity of SOD, GSH-Px, and CAT in the brain tissue and serum of the Xuesaitong group was significantly improved (Figures 2A–C,E–G, ^**^P < 0.01), and the concentration of MDA was significantly decreased (Figures 2D,H, ^**^P < 0.01).

### Total Flavonoids of Chuju Alleviates Pathomorphological Changes in Middle Cerebral Artery Occlusion Rats

**FIGURE 3:**
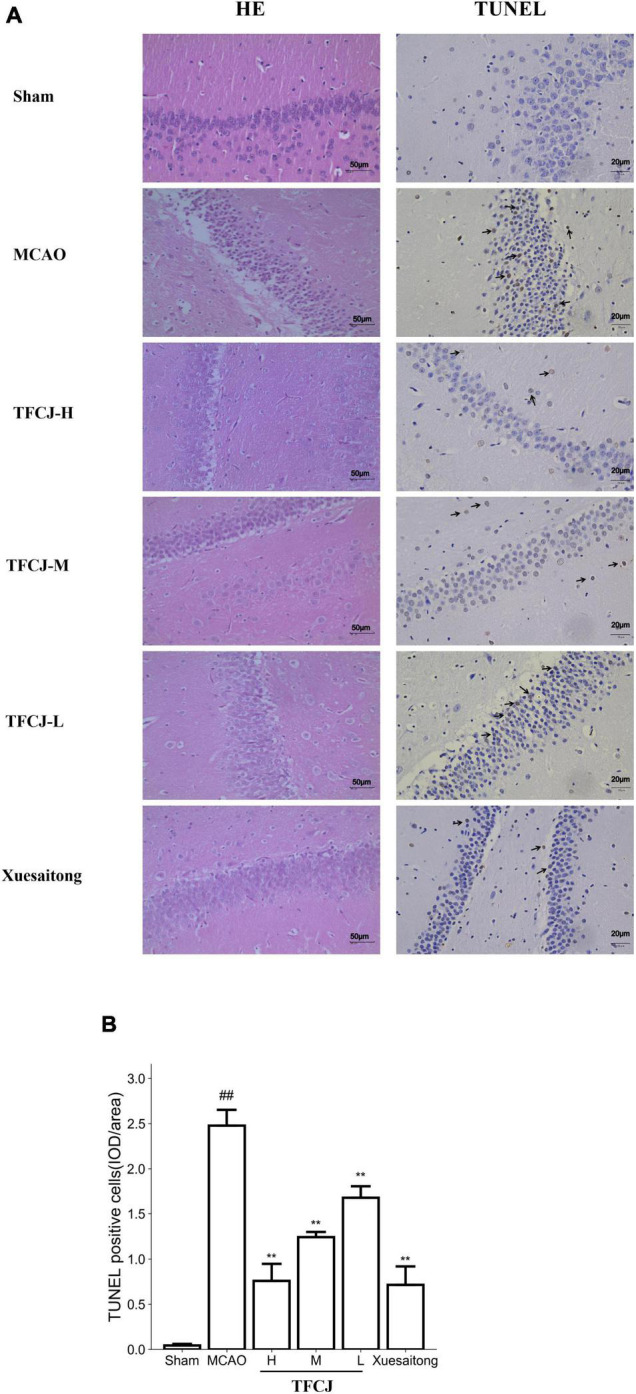
The protective effects of TFCJ on MCAO inducing histology changes. **(A)** Micrographs of HE staining, and TUNEL staining. **(B)** Effects of TFCJ on the numbers of TUNEL positive neurons. Results were showed as mean ± SD (*n* = 5), ^##,**^P < 0.01.

The structure of hippocampal neurons was intact in the sham group; the cells were regularly arranged and abundant in the cytoplasm; the cell nucleolus was large and obvious, and there was no pyknosis in the nucleus. Contrary, compared to the sham group, the neurons were disordered, and the cells were round vacuoles in the MCAO group. The arrangement of neuronal cells in the three-dose groups of TFCJ was regular, and the cell morphology was improved compared to the MCAO group (Figure 3A). Compared to the MCAO group, the neurons were neatly arranged, and the cell morphology was improved in the Xuesaitong group (Figure 3A).

### Total Flavonoids of Chuju Treatment Decreases Neuronal Apoptosis

The nucleus was stained in blue, and the TUNEL-positive neurons were brown. Almost no neuronal death was observed in the sham group. The numbers of TUNEL-positive neurons were significantly higher in the MCAO group than in the sham group (Figures 3A,B, ^##^*P* < 0.01), while they were reduced in the TFCJ group (Figure 3B, ^**^P < 0.01). Compared to the MCAO group, the numbers of TUNEL-positive neurons were significantly lower in the Xuesaitong group (Figure 3B, ^**^P < 0.01).

### The Predictive Target of Total Flavonoids of Chuju

The 12 compounds [luteolin, quercetin, isorhamnetin, kaempferol, acacetin, eupatorin, 5, 7-dihydroxy-2-(3-hydroxy-4-methoxyphenyl) chroman-4-one, linarin, diosmetin, chryseriol, naringenin, and artemetin in TFCJ are described in Supplementary Table 1]. The results retrieved from the PharmMapper databases were integrated to obtain the 300 targets with the highest matching degree with the 12 active TFCJ compounds. After deduplication, all information were merged, resulting in a database of 395 targets of TFCJ.

### Construction of the Protein–Protein Interactions and “Herb-Compound-Target-Disease” Relationship Network

**FIGURE 4:**
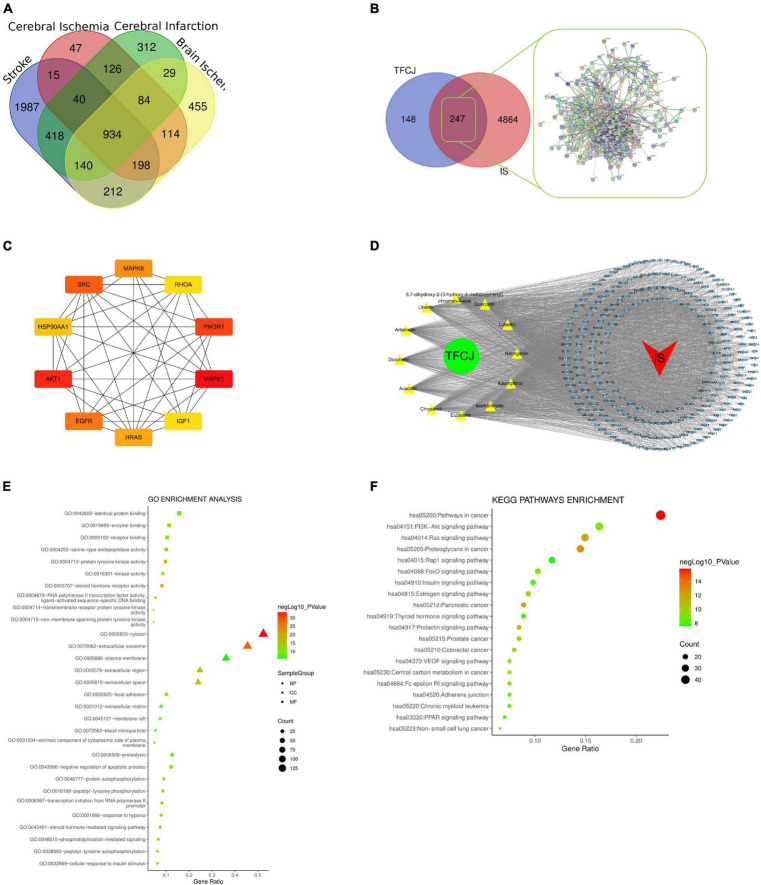
Analysis of TFCJ compound-target network. **(A)** The Venny genes of IS. **(B)** The Venny and PPI network target genes of TFCJ anti IS. **(C)** The network for core target connection. **(D)** Herb-compound-target- disease network. **(E)** The GO enrichment analysis of 247 nodes. **(F)** Top 20 of the KEGG enrichment analysis.

IS-related targets were identified from GeneCards, OMIM, and DisGeNET databases. After deduplication, all information were merged, resulting in a database of 5,111 IS-related targets, including 934 common targets (Figure 4A). A total of 247 common targets of TFCJ-anti-IS were identified using a Venn tool. The network was constructed with the String 11.0 database ignored disconnected targets, resulting in the PPI network of only 223 targets (Figure 4B). The top ten targets, selected according to the degree of the PPI network, were MAPK1, Akt1, PIK3R1, SRC, EGFR, MAPK8, HRAS, HSP90AA1, IGF1, and RHOA (Figure 4C). The herb-compound-target-disease network was composed of one herb, 12 compounds, 247 targets, and one disease. A diagram of this network is shown in Figure 4D.

### GO Enrichment Analysis and KEGG Pathway Analysis

We further analyzed the 247 targets of TFCJ-anti-IS using the David database. A total of 395 biological processes (BP), 50 cellular components (CC), 108 molecular functions (MF), and 110 KEGG pathways met the screening criteria of *P*-value < 0.05 (Supplementary Tables 2–5). The top ten terms of MF were identical protein binding, enzyme binding, receptor binding, serine-type endopeptidase activity, protein tyrosine kinase activity, kinase activity, steroid hormone receptor activity, RNA polymerase II transcription factor activity, ligand-activated sequence-specific DNA binding, transmembrane receptor protein tyrosine kinase activity, and non-membrane spanning protein tyrosine kinase activity. The top ten terms of CC were cytosol, extracellular exosome, plasma membrane, extracellular region, extracellular space, focal adhesion, extracellular matrix, membrane raft, blood microparticle, and an extrinsic component of cytoplasmic side of the plasma membrane. The top ten terms of BP were proteolysis, negative regulation of the apoptotic process, protein autophosphorylation, peptidyl-tyrosine phosphorylation, transcription initiation from RNA polymerase II promoter, response to hypoxia, steroid hormone mediated signaling pathway, phosphatidylinositol-mediated signaling, peptidyl-tyrosine autophosphorylation, and cellular response to insulin stimulus (Figure 4E).

The top 20 terms of KEGG pathways were pathways in cancer, PI3K-Akt signaling pathway, Ras signaling pathway, proteoglycans in cancer, Rap1 signaling pathway, FoxO signaling pathway, insulin signaling pathway, estrogen signaling pathway, pancreatic cancer, thyroid hormone signaling pathway, prolactin signaling pathway, prostate cancer, colorectal cancer, VEGF signaling pathway, central carbon metabolism in cancer, Fc epsilon RI signaling pathway, adherens junction, chronic myeloid leukemia, PPAR signaling pathway, and non-small cell lung cancer (Figure 4F).

### The Protein Expression of PI3K/AKT/mTOR Pathway Mediated by Total Flavonoids of Chuju

The first part of the article found that TFCJ has a brain-protective effect and was related to the inhibition of oxidative stress and cell apoptosis. As the top 20 of the KEGG pathway, the PI3K-Akt signaling pathway has an important role in apoptosis and oxidative stress. Therefore, we focused on the regulation of TFCJ on the PI3K-Akt signal pathway and its downstream mTOR signal pathway (the 86th position of the KEGG pathway).

**FIGURE 5:**
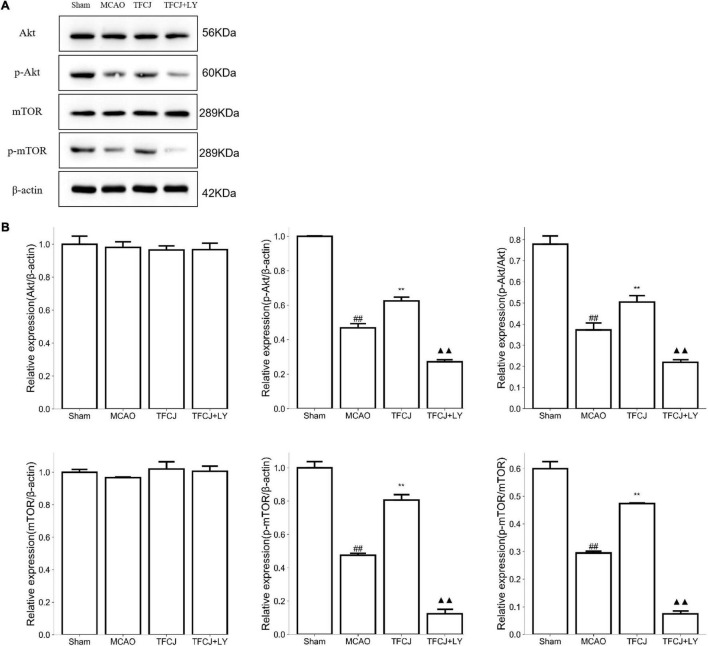
The effects of TFCJ on PI3K/Akt/mTOR pathway. **(A)** The Akt, mTOR, p-Akt, and p-mTOR expression were measured using Western blotting. **(B)** The data of Akt, mTOR, p-Akt, and p-mTOR expression. Results were shown as mean ± SD (*n* = 5), ^##,**,▲▲^*P* < 0.01.

The p-Akt and p-mTOR expression in the MCAO control rats were significantly lower than in the sham rats (Figures 5A,B, ^##^*P* < 0.01). The p-Akt and p-mTOR expression in the TFCJ treatment rats were significantly higher than in the MCAO control rats (Figure 5B, ^**^P < 0.01). After using LY294002, the TFCJ treatment was reversed (see Figure 5B, ^▲▲^*P* < 0.01). There was no significant change in total Akt and mTOR protein expression in each group (*P* > 0.05).

### Total Flavonoids of Chuju Reduces Oxidative Stress Through PI3K/AKT/mTOR Pathway

**FIGURE 6:**
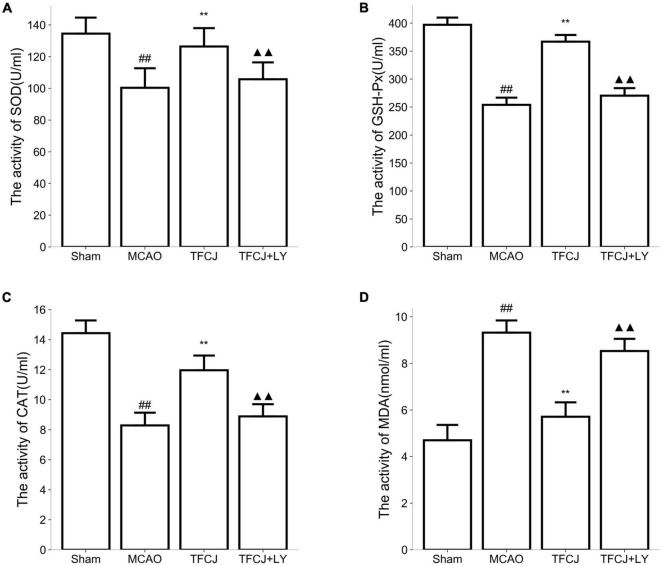
Effects of TFCJ on MCAO inducing oxidative stress. **(A)** SOD activity; **(B)** GSH-Px activity; **(C)** CAT activity; **(D)** MDA level. Results were shown as mean ± SD (*n* = 10), ^#^*P* < 0.05, ^##^*P* < 0.01 vs. sham group. ^##,**,▲▲^*P* < 0.01.

Compared with the sham group, the activities of SOD (Figure 6A), GSH-Px (Figure 6B), and CAT (Figure 6C) in the serum were significantly reduced in the MCAO control animals compared with the sham animals, while the level of MDA (Figure 6D) was significantly increased (Figures 6A–D, ^##^*P* < 0.01). In the TFCJ treatment animals, GSH-Px (Figure 6B) and CAT (Figure 6C) activities were higher, while the MDA level (Figure 6D) was lower compared to MCAO control (Figures 6A–D, ^**^P < 0.01). After using LY294002, the effect of TFCJ was reversed (Figures 6A–D, ^▲▲^*P* < 0.01). The result showed that TFCJ could inhibit oxidative stress caused by MCAO in the serum through PI3K/Akt/mTOR pathway.

### Total Flavonoids of Chuju Inhibited Apoptosis Through PI3K/AKT/mTOR Pathway

**FIGURE 7:**
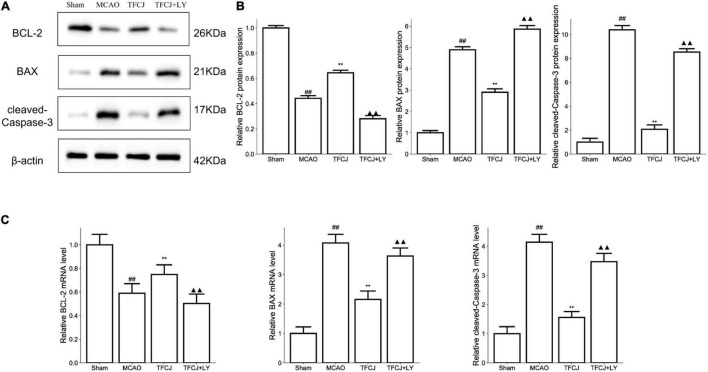
The effects of TFCJ on BCL-2, BAX, and cleaved-Caspase-3 expression. **(A,B)** The apoptosis-related factors measured using Western blotting. **(C)** The data of RT-PCR in apoptosis-related factors. Results were shown as mean ± SD (*n* = 5), ^#^*P* < 0.05, ^##^*P* < 0.01 vs. sham group. ^##,**,▲▲^*P* < 0.01.

As shown in Figure 7, the BCL-2 protein expression was reduced after IS 24h (Figure 7A, ^##^*P* < 0.01), whereas TFCJ upregulated BCL-2 protein expression (Figure 7A, **P < 0.01). The BAX and cleaved-Caspase-3 expression were increased after IS 24h (Figure 7A, ^##^*P* < 0.01), whereas TFCJ upregulated BAX and cleaved-Caspase-3 protein expression (Figure 7A, **P < 0.01). After using LY294002, the TFCJ treatment was reversed (see Figure 7A, ^▲▲^*P* < 0.01). Moreover, RT-PCR analysis indicated that MCAO reduced the expression of BCL-2 and increased the level of BAX and cleaved-Caspase-3 (Figure 7B, ^##^*P* < 0.01), while TFCJ induced the expression of BCL-2 and decreased the level of BAX and cleaved-Caspase-3 (Figure 7B, **P < 0.01). After using LY294002, the effect of TFCJ was significantly reversed (Figure 7B, ^▲▲^*P* < 0.01).

## Discussion

This study evaluated the neuroprotective effect of TFCJ against IS in rats. We hypothesized that TFCJ might exert its neuroprotective effects by suppressing apoptosis and oxidative stress by activating the PI3K/Akt/mTOR signaling pathway. The important predictors of IS are the neurological deficit scores and cerebral infarction (Zhang H. et al., 2020; Lin et al., 2021; Wang et al., 2021). In this study, rats were subjected to middle cerebral artery occlusion (MCAO) for 2 h and reperfusion for 24 h. In the early phase (first few minutes to a few hours) after intracerebral artery occlusion, the infarcted area containing ischemic tissue gradually spreads outwards. With time, the ability of brain tissue to recovery gradually decreases (Prabhakaran et al., 2015). In this study, MCAO rats showed typical IS features, including a dramatic increase of neurological deficit scores and cerebral infarction. Our data also showed that TFCJ could effectively improve neurological function and reduce the area of cerebral infarction. Moreover, in the MCAO rats, HE staining experiments showed that the neurons were arranged in a disorderly manner, and the cells were around vacuoles, while the arrangement of neuronal cells was regular, and the cell morphology was improved with the aid of TFCJ.

Next, we explored the underlying mechanism of TFCJ against IS. Previous studies (Zhang et al., 2019; Heidari et al., 2021; Luo et al., 2021) have reported that oxidative stress and cell apoptosis may have the strongest correlation with IS. Li et al. (2021) reported that miR-27A-3p reduces apoptosis and oxidative stress induced by oxygen-glucose deprivation/reoxygenation through FOXO1/p27 Kip1 signaling. Moreover, Yip et al. (2021) reported that melatonin reduces apoptotic oxidative stress by repairing impaired metabolic-redox circuits in IS.

GSH-Px is the main antioxidant that interacts with lipid hydrogen peroxide (Zhang L. et al., 2021). The degree of cerebral ischemia, hypoxia, and necrosis is related to the level of GSH-Px. The increase of GSH-Px levels can inhibit IS. SOD can have a role by scavenging oxygen free radicals in the body (Zhang L. et al., 2021). CAT can disproportionate and decompose oxygen free radicals to produce H_2_O_2_ through SOD disproportionation to avoid cytotoxicity (Wattanathorn et al., 2020). MDA is the final product of oxygen-derived free radicals and lipid peroxidation and an important sign of oxidative damage (Du et al., 2020). To assess the level of oxidative stress, we found that SOD, GSH-Px, CAT activities were reduced, while MDA level was increased in the serum and cerebral ischemic region after IS (after 24 h); while this effect was reversed by TFCJ. The results of TUNEL staining experiments showed that TFCJ markedly attenuated neuronal apoptosis, thus suggesting that TFCJ could regulate apoptosis after MCAO.

Then, potential targets and pathways of TFCJ were screened by network pharmacology. A total of 12 compounds of TFCJ were collected. We predicted 395 targets according to their structure and screened 5111 targets of IS. Finally, 247 common targets were obtained by a Venny tool. A total of 395 biological processes, 50 cellular components, 108 molecular functions, and 110 KEGG pathways were obtained by GO and KEGG analysis of 247 common targets.

PI3K/Akt/mTOR is a multifunctional signaling pathway associated with anti-apoptosis and anti-oxidant stress. Phosphorylation of Akt and mTOR were suppressed in the late stage of IS. Akt and mTOR overexpression improved the protective effects after cerebral ischemia-reperfusion injury in MCAO rats, suggesting that the activation of the PI3K/Akt/mTOR signaling pathway provides significant neuroprotective effects against cerebral ischemia-reperfusion injury. P-Akt and p-mTOR (Li et al., 2016; Yang et al., 2017; Park et al., 2019; Sun et al., 2020) are commonly recognized as markers for activation of the PI3K/Akt/mTOR pathway. In the present study, the levels of p-AKT and p-mTOR were significantly decreased following I/R injury and were activated by TFCJ treatment.

To further explore the molecular mechanisms underlying the TFCJ anti-apoptosis and anti-oxidant stress effect, we detected the expression changes of mediators of apoptosis and oxidant stress involved in ischemic brain damage under the inhibitory condition of PI3K/Akt/mTOR signaling pathway. BCL-2 is a type of anti-apoptotic protein, while BAX and cleaved-Caspase-3 are known to promote apoptosis (Veleta et al., 2020; Wei et al., 2020). The protein expression of BCL-2, BAX, and cleaved-Caspase-3 were detected by using western blot to evaluate the degree of apoptosis. The levels of BCL-2, SOD, CAT, and GSH-Px significantly decreased, while BAX, cleaved-Caspase-3, and MDA levels were increased following I/R injury; this process was reversed by TFCJ treatment. Moreover, the effect of TFCJ was significantly reversed when applying LY294002 (PI3k inhibitor).

In summary, our data suggested that TFCJ might decrease nerve damage in rat cerebral ischemia by regulating the PI3K/Akt signaling pathway. The findings of this study provide a reference for the clinical anti-apoptosis, anti-oxidant stress, and neuroprotection of TFCJ. In our future studies, we plan to further assess the effects of TFCJ on cell apoptosis and oxidative stress *in vitro*.

## Data Availability Statement

The original contributions presented in the study are included in the article/Supplementary Material, further inquiries can be directed to the corresponding author.

## Ethics Statement

The animal study was reviewed and approved by Anhui Science and Technology University institutional animal care. Written informed consent was obtained from the owners for the participation of their animals in this study.

## Author Contributions

CW, HC, B-BM, and HY contributed to conception and design of the study. CW organized the database and wrote the first draft of the manuscript. CW and H-HJ performed the statistical analysis. H-HJ wrote sections of the manuscript. All authors contributed to manuscript revision, read, and approved the submitted version.

## Conflict of Interest

The authors declare that the research was conducted in the absence of any commercial or financial relationships that could be construed as a potential conflict of interest.

## Publisher’s Note

All claims expressed in this article are solely those of the authors and do not necessarily represent those of their affiliated organizations, or those of the publisher, the editors and the reviewers. Any product that may be evaluated in this article, or claim that may be made by its manufacturer, is not guaranteed or endorsed by the publisher.

## Footnotes

1
http://tcmspw.com/tcmsp.php

2
http://www.tcmip.cn/

3
http://bionet.ncpsb.org.cn/batman-tcm/

4
http://www.lilab-ecust.cn/pharmmapper/

5
https:/www.genecards.org/

6
https://www.omim.org/

7
https://www.disgenet.org/

8
https://string-db.org/

9
https://david.ncifcrf.gov/
