# Supplementary material for: Total Flavonoids of Chuju Decrease Oxidative Stress and Cell Apoptosis in Ischemic Stroke Rats: Network and Experimental Analyses
Source: Front Neurosci. 2021 Dec 9;15:772401. doi: 10.3389/fnins.2021.772401 (PMC8695723; doi:10.3389/fnins.2021.772401)
Supplement: Supplementary file 1 [file Data_Sheet_1.docx]

Supplementary Fig. 1 The original WB images.

(A)The BAX original WB images.(B)The BCL-2 original WB images.

(C)The Caspase-3 original WB images.(D)The β-actin original WB images.

(E)The Akt original WB images.(F)The p-Akt original WB images.

(G)The mTOR original WB images.(H)The p-mTOR original WB images.

(I)The β-actin original WB images.

| 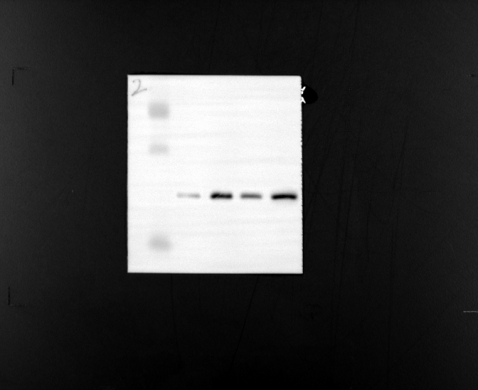 | 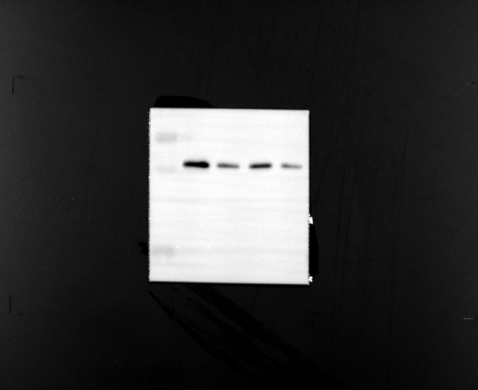 |
| --- | --- |
| (A) | (B) |
| 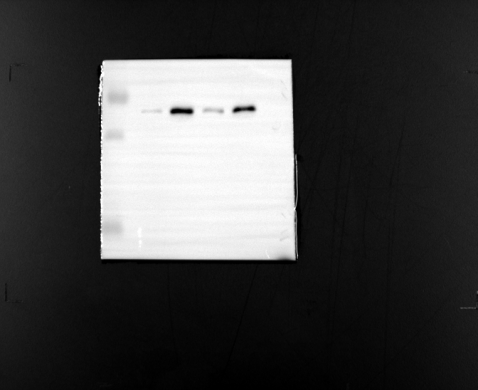 | 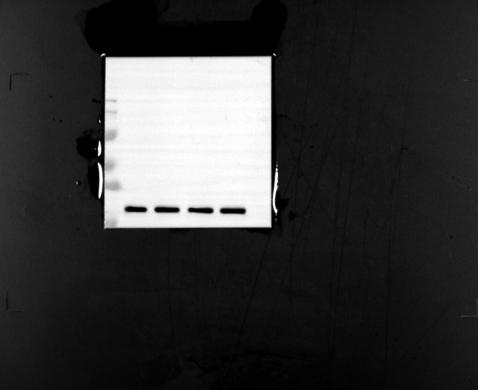 |
| (C) | (D) |
| 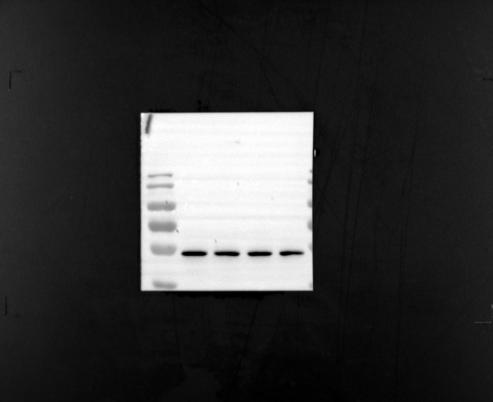 | 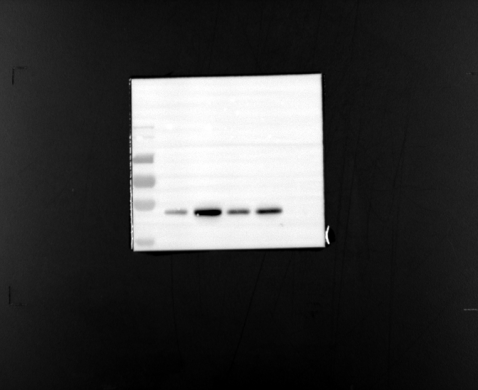 |
| (E) | (F) |
| 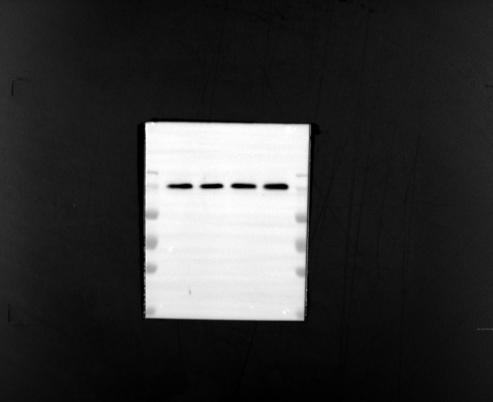 | 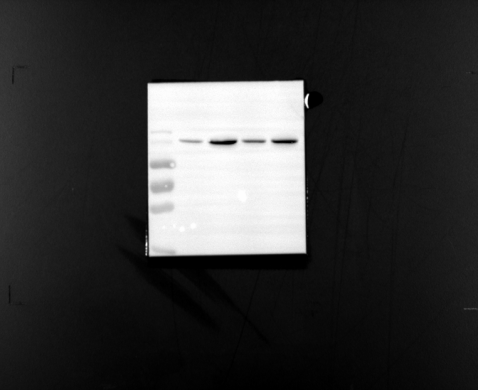 |
| (G) | (H) |
| 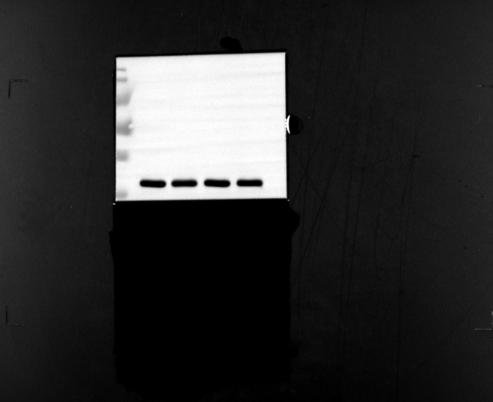 |  |
| (I) |  |
