# Supplementary material for: Total Flavonoids of Chuju Decrease Oxidative Stress and Cell Apoptosis in Ischemic Stroke Rats: Network and Experimental Analyses
Source: Front Neurosci. 2021 Dec 9;15:772401. doi: 10.3389/fnins.2021.772401 (PMC8695723; doi:10.3389/fnins.2021.772401)
Supplement: Supplementary file 2 [file Table_1.docx]

Supplementary Table 1 Basic information of ingredients in TFCJ

| CAS | Molecule Name | MW | OB (%) | Caco-2 | BBB | DL | HL |
| --- | --- | --- | --- | --- | --- | --- | --- |
| 491-70-3 | luteolin | 286.25 | 36.16 | 0.19 | -0.84 | 0.25 | 15.94 |
| 117-39-5 | quercetin | 302.25 | 46.43 | 0.05 | -0.77 | 0.28 | 14.4 |
| 480-19-3 | isorhamnetin | 316.28 | 49.6 | 0.31 | -0.54 | 0.31 | 14.34 |
| 520-18-3 | kaempferol | 286.25 | 41.88 | 0.26 | -0.55 | 0.24 | 14.74 |
| 480-44-4 | acacetin | 284.28 | 34.97 | 0.67 | -0.05 | 0.24 | 17.25 |
| 855-96-9 | eupatorin | 344.34 | 30.23 | 0.7 | -0.5 | 0.37 | 15.21 |
| 480-36-4 | linarin | 592.6 | 39.84 | -1.68 | -2.77 | 0.71 | 16.07 |
| [520-34-3](http://www.ichemistry.cn/chemistry/520-34-3.htm) | diosmetin | 300.28 | 31.14 | 0.46 | -0.66 | 0.27 | 16.34 |
| 491-71-4 | chryseriol | 300.28 | 35.85 | 0.39 | -0.53 | 0.27 | 16.31 |
| 480-41-1 | naringenin | 272.27 | 59.29 | 0.28 | -0.37 | 0.21 | 16.98 |
| 520-33-2 | 5,7-dihydroxy-2-(3-hydroxy-4-methoxyphenyl)chroman-4-one | 302.3 | 47.74 | 0.28 | -0.3 | 0.27 | 16.51 |
| 479-90-3 | artemetin | 388.4 | 49.55 | 0.81 | -0.09 | 0.48 | 15.01 |
