# Supplementary material for: Total Flavonoids of Chuju Decrease Oxidative Stress and Cell Apoptosis in Ischemic Stroke Rats: Network and Experimental Analyses
Source: Front Neurosci. 2021 Dec 9;15:772401. doi: 10.3389/fnins.2021.772401 (PMC8695723; doi:10.3389/fnins.2021.772401)
Supplement: Supplementary file 3 [file Table_2.docx]

Supplementary Table 2 Functions of potential target genes based on GO molecular function

| Category | Term | Count | PValue | Genes | FDR |
| --- | --- | --- | --- | --- | --- |
| GOTERM_MF_DIRECT | GO:0003707~steroid hormone receptor activity | 20 | 1.63E-21 | THRA, VDR, NR1H2, NR1I2, NR1H4, NR1H3, RORA, ESRRG, NR3C1, ESR1, ESR2, NR3C2, RXRB, AR, RXRA, RARB, PGR, PPARG, PPARA, PPARD | 9.61E-19 |
| GOTERM_MF_DIRECT | GO:0004713~protein tyrosine kinase activity | 24 | 1.69E-18 | MAP2K1, HSP90AA1, SYK, SRC, INSR, EGFR, PTK2, IGF1R, ZAP70, HCK, ERBB4, LCK, KIT, KDR, BTK, ABL1, CSK, TEK, JAK2, JAK3, MET, FGFR2, EPHA2, FGFR1 | 4.98E-16 |
| GOTERM_MF_DIRECT | GO:0004879~RNA polymerase II transcription factor activity, ligand-activated sequence-specific DNA binding | 13 | 6.44E-14 | NR1H2, NR1I2, NR1H4, NR1H3, RORA, ESR1, ESR2, RXRB, AR, RXRA, PPARG, PPARA, PPARD | 1.27E-11 |
| GOTERM_MF_DIRECT | GO:0004252~serine-type endopeptidase activity | 25 | 4.23E-13 | CFD, C1S, C1R, CTSS, DPP4, PLAU, CTSG, CTSD, ELANE, CTSB, MMP7, F10, MMP1, CMA1, MMP2, MMP3, F11, MMP8, F2, MMP9, MMP12, F7, MMP13, FAP, CFB | 5.84E-11 |
| GOTERM_MF_DIRECT | GO:0019899~enzyme binding | 28 | 4.94E-13 | SRC, EGFR, ACAT1, MAPK8, RXRA, CBS, AKT1, HMOX1, RAC1, PTPN1, BCHE, HSPA8, GSTM1, PARP1, STAT1, PDE4D, MAPK14, ESR1, ESR2, BACE1, FKBP1A, AR, MDM2, PPARG, PGR, RAF1, HSPA1B, HSPA1A | 5.84E-11 |
| GOTERM_MF_DIRECT | GO:0042802~identical protein binding | 39 | 1.64E-11 | SERPINA1, AHCY, C1S, SHMT1, XIAP, EGFR, SRM, IGF1R, DPP4, CDC42, TTR, IMPA1, CBS, AKT1, MAPK1, CSK, ACADM, NQO1, BCHE, G6PD, HSP90AA1, PARP1, APAF1, DAPK1, STAT1, LYZ, SOD2, MMP9, ESR1, NMNAT1, LCK, ALB, MDM2, BTK, PPARG, HPRT1, RAF1, FGFR1, BCL2L1 | 1.62E-09 |
| GOTERM_MF_DIRECT | GO:0005102~receptor binding | 26 | 7.18E-11 | SRC, GBA, DPP4, ABL1, CSK, JAK2, JAK3, TGFB2, NOS2, EPHX2, FGG, MIF, F2, PTK2, F7, AR, ZAP70, HCK, BTK, REN, ANG, PGR, AGXT, CRAT, HSPA1B, HSPA1A | 6.05E-09 |
| GOTERM_MF_DIRECT | GO:0004714~transmembrane receptor protein tyrosine kinase activity | 11 | 1.19E-10 | ERBB4, INSR, KIT, KDR, TEK, MET, EPHB4, EGFR, FGFR2, EPHA2, IGF1R | 8.77E-09 |
| GOTERM_MF_DIRECT | GO:0016301~kinase activity | 21 | 3.95E-10 | GSK3B, PKLR, CSNK2A1, DAPK1, PDPK1, SRC, PIK3R1, PIK3CG, MAPK8, PLAU, LCK, CHEK1, BTK, AKT1, MAPK1, CSK, JAK2, RAF1, FGFR2, EPHA2, CDK5R1 | 2.59E-08 |
| GOTERM_MF_DIRECT | GO:0004715~non-membrane spanning protein tyrosine kinase activity | 11 | 9.26E-10 | ZAP70, HCK, SYK, SRC, LCK, BTK, ABL1, CSK, JAK2, JAK3, PTK2 | 5.46E-08 |
| GOTERM_MF_DIRECT | GO:0005524~ATP binding | 53 | 2.42E-09 | GSK3B, ADK, PIK3CG, IGF1R, HK1, AKT2, CHEK1, KDR, AKT1, JAK2, PRKACA, JAK3, EPHB4, MAP2K1, HSP90AA1, CSNK2A1, APAF1, SYK, PDPK1, DAPK1, TAP1, TGFBR1, TGFBR2, ZAP70, HCK, LCK, KIT, BTK, RAF1, MET, EPHA2, SRC, EGFR, MAPK8, ERBB4, ABL1, PMS2, MAPK1, CSK, HSPA8, PKLR, INSR, MAPK14, GCK, PTK2, MAPK10, CDK6, NMNAT1, TEK, FGFR2, HSPA1B, FGFR1, HSPA1A | 1.30E-07 |
| GOTERM_MF_DIRECT | GO:0004672~protein kinase activity | 24 | 3.00E-09 | GSK3B, MAP2K1, SYK, CSNK2A1, DAPK1, PDPK1, SRC, MAPK14, EGFR, TGFBR1, PIK3CG, PTK2, MAPK10, CCL5, AKT2, CHEK1, BTK, ABL1, AKT1, TEK, JAK2, RAF1, MET, CDK5R1 | 1.44E-07 |
| GOTERM_MF_DIRECT | GO:0005496~steroid binding | 9 | 3.17E-09 | AR, SULT1E1, ESRRG, PGR, NR3C1, SHBG, ESR1, ESR2, NR3C2 | 1.44E-07 |
| GOTERM_MF_DIRECT | GO:0042803~protein homodimerization activity | 34 | 7.42E-09 | MAOB, SHMT1, NPR3, HMGCR, ADH5, SRM, ACAT1, DPP4, EEA1, IMPA1, ERBB4, CBS, CCL5, HMOX1, TGFB2, G6PD, HSP90AA1, GSTM1, NOS2, STAT1, EPHX2, APOA2, TAP1, GSTZ1, FKBP1A, FAP, KIT, LCN2, ANG, HPRT1, AGXT, FGFR2, FGFR1, BCL2L1 | 3.13E-07 |
| GOTERM_MF_DIRECT | GO:0008144~drug binding | 12 | 1.25E-08 | DHFR, CYP2C9, ACE, PNP, GSTP1, PDE4D, ALB, NR1I2, RARB, PPARG, PPARA, PPARD | 4.92E-07 |
| GOTERM_MF_DIRECT | GO:0008270~zinc ion binding | 44 | 1.55E-08 | THRA, ADH1C, GLO1, NR1I2, XIAP, RORA, NR3C1, ADH5, NR3C2, CRYZ, EEA1, RXRB, CA1, RXRA, CA2, LTA4H, PTPN1, MMP7, ACE, PARP1, MME, MMP1, VDR, NR1H2, MMP2, MMP3, NR1H4, NR1H3, ESRRG, MMP8, MMP9, ESR1, ESR2, MMP12, AR, BHMT, MMP13, MDM2, RARB, PPARG, PGR, PPARA, S100A9, PPARD | 5.71E-07 |
| GOTERM_MF_DIRECT | GO:0004175~endopeptidase activity | 10 | 7.69E-08 | MMP12, ACE, MME, FAP, MMP1, CMA1, MMP3, CASP1, MMP9, ELANE | 2.67E-06 |
| GOTERM_MF_DIRECT | GO:0005515~protein binding | 169 | 1.37E-07 | RORA, NR3C1, IGF1R, NR3C2, NDST1, LGALS3, LGALS2, PLAU, AKT2, CHEK1, KDR, AKT1, PRKACA, EPHB4, ARSA, MAP2K1, G6PD, CSNK2A1, DAPK1, TAP1, MIF, AR, RBP4, MTAP, ADAM17, BTK, PGR, PADI4, HPRT1, RAF1, PPIA, S100A9, EPHA2, RTN4R, FECH, SHMT1, PIK3R1, DPP4, TTPA, ABL1, HMOX1, LTA4H, ELANE, HSPA8, TGFB2, VDR, INSR, ESRRG, IGF1, SELE, ESR1, BMP7, ESR2, PTK2, SELP, CDK6, CD209, ALB, MDM2, RAB5A, FGFR2, BCL2L1, FGFR1, GSK3B, ARF1, SERPINA1, THRA, PDE3B, RND3, PIK3CG, HK1, EEA1, CASP7, CA1, IMPA1, CA2, CASP3, NCS1, CASP1, CTSG, RAC1, JAK2, JAK3, HRAS, CTSD, CTSB, HSP90AA1, TPI1, APAF1, PARP1, SYK, MME, PDPK1, GSTO1, PDE4D, MMP2, ANXA5, FGG, MMP3, APOA2, GP1BA, F2, MMP9, RHOA, TGFBR1, TGFBR2, BACE1, F7, ZAP70, HCK, RAP2A, LCK, KIT, ANG, PPARG, PPARA, AGXT, MET, PPARD, AHCY, C1S, SRC, C1R, GSTP1, GBA, NR1I2, XIAP, HMGCR, EGFR, SRM, RXRB, CDC42, MAPK8, TTR, RXRA, SULT1E1, ERBB4, CBS, CCL5, CTNNA1, PMS2, MAPK1, CSK, PTPN1, NQO1, F10, NOS2, STAT1, NOS3, NR1H2, F11, NR1H4, NR1H3, PTPN11, MAPK14, RAB11A, GCK, GSTZ1, MAPK10, FKBP1A, FABP3, NMNAT1, FABP5, FAP, TEK, RAN, HSPA1B, CDK5R1, HSPA1A | 4.48E-06 |
| GOTERM_MF_DIRECT | GO:0004716~receptor signaling protein tyrosine kinase activity | 6 | 1.50E-07 | SYK, ERBB4, INSR, KIT, KDR, EGFR | 4.65E-06 |
| GOTERM_MF_DIRECT | GO:0046934~phosphatidylinositol-4,5-bisphosphate 3-kinase activity | 9 | 3.11E-06 | ERBB4, LCK, KIT, PTPN11, PIK3R1, EGFR, FGFR2, PIK3CG, FGFR1 | 9.18E-05 |
| GOTERM_MF_DIRECT | GO:0019903~protein phosphatase binding | 9 | 3.52E-06 | HSP90AA1, LCK, CSK, PPARG, PIK3R1, MAPK14, JAK3, MET, EGFR | 9.89E-05 |
| GOTERM_MF_DIRECT | GO:0005158~insulin receptor binding | 7 | 4.89E-06 | PTPN1, PDPK1, SRC, PTPN11, IGF1, PIK3R1, IGF1R | 1.31E-04 |
| GOTERM_MF_DIRECT | GO:0004222~metalloendopeptidase activity | 11 | 6.00E-06 | MMP12, ADAM17, MMP13, MMP7, MME, FAP, MMP1, MMP2, MMP3, MMP8, MMP9 | 1.54E-04 |
| GOTERM_MF_DIRECT | GO:0043560~insulin receptor substrate binding | 5 | 1.34E-05 | INSR, PTPN11, PIK3R1, JAK2, IGF1R | 3.29E-04 |
| GOTERM_MF_DIRECT | GO:0005088~Ras guanyl-nucleotide exchange factor activity | 10 | 4.70E-05 | ERBB4, KIT, TEK, JAK2, JAK3, PTK2, EGFR, IL2, FGFR2, FGFR1 | 0.001110229 |
| GOTERM_MF_DIRECT | GO:0008233~peptidase activity | 9 | 5.03E-05 | BACE1, FOLH1, FAP, CASP3, REN, CTSG, LTA4H, ELANE, CTSB | 0.00114058 |
| GOTERM_MF_DIRECT | GO:0046965~retinoid X receptor binding | 5 | 5.29E-05 | VDR, NR1H2, NR1H4, RARB, PPARG | 0.001156868 |
| GOTERM_MF_DIRECT | GO:0019901~protein kinase binding | 17 | 1.34E-04 | PTPN1, GSK3B, MAP2K1, SYK, PARP1, PDPK1, EGFR, PTK2, CDC42, RHEB, LCK, NCS1, RAC1, JAK2, PRKACA, CDK5R1, BCL2L1 | 0.002732786 |
| GOTERM_MF_DIRECT | GO:0050661~NADP binding | 6 | 1.43E-04 | DHFR, G6PD, NOS2, NOS3, GSR, HMGCR | 0.002732786 |
| GOTERM_MF_DIRECT | GO:0005504~fatty acid binding | 5 | 1.44E-04 | FABP4, FABP5, ALB, ADH5, PPARD | 0.002732786 |
| GOTERM_MF_DIRECT | GO:0043548~phosphatidylinositol 3-kinase binding | 5 | 1.44E-04 | LCK, INSR, PIK3R1, JAK2, IGF1R | 0.002732786 |
| GOTERM_MF_DIRECT | GO:0030235~nitric-oxide synthase regulator activity | 4 | 1.62E-04 | HSP90AA1, AKT1, ESR1, EGFR | 0.002990342 |
| GOTERM_MF_DIRECT | GO:0043565~sequence-specific DNA binding | 20 | 2.21E-04 | THRA, VDR, NR1H2, NR1I2, NR1H4, NR1H3, RORA, ESRRG, NR3C1, ESR1, ESR2, NR3C2, RXRB, AR, RXRA, RARB, PGR, PPARG, PPARA, PPARD | 0.003951334 |
| GOTERM_MF_DIRECT | GO:0016597~amino acid binding | 5 | 2.62E-04 | TPH1, SHMT1, PAH, AGXT, OTC | 0.00454354 |
| GOTERM_MF_DIRECT | GO:0009055~electron carrier activity | 8 | 3.43E-04 | GCDH, MAOB, ALDH2, GSR, AKR1B1, ACADM, CYP19A1, ADH5 | 0.005774613 |
| GOTERM_MF_DIRECT | GO:0004674~protein serine/threonine kinase activity | 16 | 4.30E-04 | GSK3B, MAP2K1, SYK, CSNK2A1, DAPK1, PDPK1, MAPK14, TGFBR1, PIK3CG, MAPK8, AKT2, CHEK1, AKT1, MAPK1, RAF1, PRKACA | 0.007048139 |
| GOTERM_MF_DIRECT | GO:0005525~GTP binding | 16 | 5.34E-04 | ARF1, HSP90AA1, DAPK1, INSR, RND3, RHOA, RAB11A, CDC42, RAP2A, RHEB, RAC2, RAC1, PCK1, HRAS, RAB5A, RAN | 0.008507643 |
| GOTERM_MF_DIRECT | GO:0003924~GTPase activity | 12 | 6.62E-04 | CDC42, RAP2A, ARF1, RHEB, RAC2, RAC1, HRAS, RND3, RAB5A, RHOA, RAN, RAB11A | 0.01027989 |
| GOTERM_MF_DIRECT | GO:0016491~oxidoreductase activity | 11 | 7.22E-04 | HSD11B1, CYP2C9, MAOB, ALDH2, ADH1C, GSTO1, NOS3, GSR, AKR1B1, ADH5, CRYZ | 0.010916119 |
| GOTERM_MF_DIRECT | GO:0005178~integrin binding | 8 | 8.68E-04 | ADAM17, SYK, FAP, SRC, ICAM2, KDR, IGF1, EGFR | 0.012803376 |
| GOTERM_MF_DIRECT | GO:0004707~MAP kinase activity | 4 | 9.88E-04 | MAPK10, MAPK8, MAPK1, MAPK14 | 0.014218593 |
| GOTERM_MF_DIRECT | GO:0008237~metallopeptidase activity | 7 | 0.001174861 | ADAM17, ACE, MME, MMP2, MMP3, LTA4H, MMP9 | 0.016503998 |
| GOTERM_MF_DIRECT | GO:0005159~insulin-like growth factor receptor binding | 4 | 0.001221854 | INSR, REN, IGF1, PIK3R1 | 0.016690707 |
| GOTERM_MF_DIRECT | GO:0051022~Rho GDP-dissociation inhibitor binding | 3 | 0.001244731 | CDC42, HSP90AA1, RAC1 | 0.016690707 |
| GOTERM_MF_DIRECT | GO:0004190~aspartic-type endopeptidase activity | 5 | 0.001291807 | BACE1, CASP7, CASP3, REN, CTSD | 0.016937028 |
| GOTERM_MF_DIRECT | GO:0004197~cysteine-type endopeptidase activity | 6 | 0.001931839 | CASP7, CASP3, CASP1, CTSD, CTSS, CTSB | 0.02477794 |
| GOTERM_MF_DIRECT | GO:0043559~insulin binding | 3 | 0.002054605 | INSR, PIK3R1, IGF1R | 0.025254522 |
| GOTERM_MF_DIRECT | GO:0003708~retinoic acid receptor activity | 3 | 0.002054605 | RXRA, RARB, ESRRG | 0.025254522 |
| GOTERM_MF_DIRECT | GO:0008236~serine-type peptidase activity | 6 | 0.002229414 | CFD, DPP4, F7, FAP, C1R, CMA1 | 0.026843966 |
| GOTERM_MF_DIRECT | GO:0050660~flavin adenine dinucleotide binding | 6 | 0.002390103 | GCDH, MAOB, NOS2, NOS3, GSR, ACADM | 0.028037876 |
| GOTERM_MF_DIRECT | GO:0008201~heparin binding | 9 | 0.002423613 | SELP, MMP7, F11, CTSG, ANG, BMP7, FGFR2, ELANE, FGFR1 | 0.028037876 |
| GOTERM_MF_DIRECT | GO:0001948~glycoprotein binding | 6 | 0.002559048 | SELP, F7, HSP90AA1, SERPINA1, LCK, EGFR | 0.029035354 |
| GOTERM_MF_DIRECT | GO:0055131~C3HC4-type RING finger domain binding | 3 | 0.003052311 | HSPA8, HSPA1B, HSPA1A | 0.033978561 |
| GOTERM_MF_DIRECT | GO:0016303~1-phosphatidylinositol-3-kinase activity | 5 | 0.003477569 | PTPN11, PIK3R1, FGFR2, PIK3CG, FGFR1 | 0.037995657 |
| GOTERM_MF_DIRECT | GO:0002020~protease binding | 7 | 0.003607448 | DPP4, SERPINA1, FAP, CASP3, KIT, LCN2, ELANE | 0.038698076 |
| GOTERM_MF_DIRECT | GO:0020037~heme binding | 8 | 0.003967262 | CYP2C9, NOS2, SRC, CBS, NOS3, HMOX1, JAK2, CYP19A1 | 0.041797942 |
| GOTERM_MF_DIRECT | GO:0016004~phospholipase activator activity | 3 | 0.004232248 | GM2A, PDPK1, CCL5 | 0.043807475 |
| GOTERM_MF_DIRECT | GO:0004114~3',5'-cyclic-nucleotide phosphodiesterase activity | 4 | 0.0043643 | PDE4D, PDE3B, PDE4B, PDE5A | 0.044395466 |
| GOTERM_MF_DIRECT | GO:0051117~ATPase binding | 6 | 0.004491044 | AR, LCK, NR1H2, PDE4D, PGR, ESR1 | 0.044910443 |
| GOTERM_MF_DIRECT | GO:0019825~oxygen binding | 5 | 0.004803618 | CYP2C9, CBS, ALB, SOD2, CYP19A1 | 0.047235576 |
| GOTERM_MF_DIRECT | GO:0004887~thyroid hormone receptor activity | 3 | 0.005588934 | THRA, NR1I2, NR1H4 | 0.054056904 |
| GOTERM_MF_DIRECT | GO:0030145~manganese ion binding | 5 | 0.005995547 | IMPA1, ARG1, ABL1, PCK1, SOD2 | 0.057054401 |
| GOTERM_MF_DIRECT | GO:0046875~ephrin receptor binding | 4 | 0.006204878 | PTPN1, SRC, PIK3CG, CDK5R1 | 0.058109173 |
| GOTERM_MF_DIRECT | GO:0051721~protein phosphatase 2A binding | 4 | 0.006906328 | PTPN1, STAT1, AKT1, HMGCR | 0.063667713 |
| GOTERM_MF_DIRECT | GO:0005543~phospholipid binding | 6 | 0.008439919 | SEC14L2, F10, ANXA5, PLA2G2A, APOA2, OTC | 0.07549726 |
| GOTERM_MF_DIRECT | GO:0042169~SH2 domain binding | 4 | 0.008445456 | SRC, LCK, JAK2, PTK2 | 0.07549726 |
| GOTERM_MF_DIRECT | GO:0046790~virion binding | 3 | 0.008811237 | APCS, CD209, PPIA | 0.077591489 |
| GOTERM_MF_DIRECT | GO:0031625~ubiquitin protein ligase binding | 11 | 0.009590488 | HSPA8, GSK3B, GPI, TPI1, CBS, PDE4D, MDM2, PRKACA, EGFR, HSPA1B, HSPA1A | 0.081794988 |
| GOTERM_MF_DIRECT | GO:0005215~transporter activity | 9 | 0.009692894 | SEC14L2, FABP3, RBP4, FABP4, FABP5, TTPA, FABP7, LCN2, TAP1 | 0.081794988 |
| GOTERM_MF_DIRECT | GO:0008083~growth factor activity | 8 | 0.00970449 | GPI, TGFB2, REG1A, IGF1, F2, BMP7, IL2, TYMP | 0.081794988 |
| GOTERM_MF_DIRECT | GO:0005536~glucose binding | 3 | 0.010666484 | G6PD, GCK, HK1 | 0.087730354 |
| GOTERM_MF_DIRECT | GO:0042277~peptide binding | 5 | 0.010706077 | MME, CMA1, ANG, LTA4H, PPIA | 0.087730354 |
| GOTERM_MF_DIRECT | GO:0042623~ATPase activity, coupled | 3 | 0.01267774 | HSPA8, HSPA1B, HSPA1A | 0.101079275 |
| GOTERM_MF_DIRECT | GO:0004708~MAP kinase kinase activity | 3 | 0.01267774 | MAPK10, MAP2K1, MAPK14 | 0.101079275 |
| GOTERM_MF_DIRECT | GO:0004364~glutathione transferase activity | 4 | 0.014192115 | GSTZ1, GSTM1, GSTO1, GSTP1 | 0.111644635 |
| GOTERM_MF_DIRECT | GO:0097153~cysteine-type endopeptidase activity involved in apoptotic process | 3 | 0.0148401 | CASP7, CASP3, CASP1 | 0.115206039 |
| GOTERM_MF_DIRECT | GO:0042826~histone deacetylase binding | 6 | 0.016762238 | HSP90AA1, MAPK8, PARP1, RAC1, HSPA1B, HSPA1A | 0.128437924 |
| GOTERM_MF_DIRECT | GO:0010181~FMN binding | 3 | 0.019599066 | NOS2, NOS3, PNPO | 0.144543113 |
| GOTERM_MF_DIRECT | GO:0004115~3',5'-cyclic-AMP phosphodiesterase activity | 3 | 0.019599066 | PDE4D, PDE3B, PDE4B | 0.144543113 |
| GOTERM_MF_DIRECT | GO:0070402~NADPH binding | 3 | 0.019599066 | DHFR, HMGCR, CRYZ | 0.144543113 |
| GOTERM_MF_DIRECT | GO:0051082~unfolded protein binding | 6 | 0.022455666 | HSPA8, APCS, HSP90AA1, PPIA, HSPA1B, HSPA1A | 0.163565963 |
| GOTERM_MF_DIRECT | GO:0031072~heat shock protein binding | 4 | 0.023115459 | HSPA8, APAF1, HSPA1B, HSPA1A | 0.166318549 |
| GOTERM_MF_DIRECT | GO:0008134~transcription factor binding | 10 | 0.023736828 | AR, THRA, PARP1, MAPK1, PPARG, RORA, PIK3R1, PPARA, ESR1, PPARD | 0.168731671 |
| GOTERM_MF_DIRECT | GO:0001046~core promoter sequence-specific DNA binding | 4 | 0.024588301 | PPARG, RORA, ESR1, ESR2 | 0.170868787 |
| GOTERM_MF_DIRECT | GO:0031435~mitogen-activated protein kinase kinase kinase binding | 3 | 0.024906298 | CDC42, MAPK1, TGFBR2 | 0.170868787 |
| GOTERM_MF_DIRECT | GO:0008656~cysteine-type endopeptidase activator activity involved in apoptotic process | 3 | 0.024906298 | APAF1, CASP3, CASP1 | 0.170868787 |
| GOTERM_MF_DIRECT | GO:0098641~cadherin binding involved in cell-cell adhesion | 10 | 0.026686775 | HSPA8, PTPN1, STAT1, SRC, CTNNA1, EGFR, HSPA1B, RAN, EPHA2, HSPA1A | 0.180979282 |
| GOTERM_MF_DIRECT | GO:0043274~phospholipase binding | 3 | 0.027754374 | PDPK1, PTPN11, SELE | 0.186080464 |
| GOTERM_MF_DIRECT | GO:0038052~RNA polymerase II transcription factor activity, estrogen-activated sequence-specific DNA binding | 2 | 0.028933683 | ESR1, ESR2 | 0.187592011 |
| GOTERM_MF_DIRECT | GO:0004645~phosphorylase activity | 2 | 0.028933683 | MTAP, TYMP | 0.187592011 |
| GOTERM_MF_DIRECT | GO:0004053~arginase activity | 2 | 0.028933683 | ARG2, ARG1 | 0.187592011 |
| GOTERM_MF_DIRECT | GO:0005080~protein kinase C binding | 4 | 0.029303809 | HINT1, SRC, ABL1, AKT1 | 0.187926598 |
| GOTERM_MF_DIRECT | GO:0000287~magnesium ion binding | 8 | 0.030205714 | ARF1, PKLR, IMPA1, EPHX2, NCS1, ABL1, HPRT1, PCK1 | 0.191627647 |
| GOTERM_MF_DIRECT | GO:0008013~beta-catenin binding | 5 | 0.031821564 | GSK3B, AR, CTNNA1, RORA, ESR1 | 0.199731091 |
| GOTERM_MF_DIRECT | GO:0097110~scaffold protein binding | 4 | 0.032693802 | SRC, NOS3, PDE4D, MDM2 | 0.203045715 |
| GOTERM_MF_DIRECT | GO:0005509~calcium ion binding | 18 | 0.034227167 | APCS, ARSA, C1S, F10, C1R, MMP1, PLA2G2A, ANXA5, MMP3, F2, MMP8, MMP12, F7, MMP13, NCS1, PADI4, S100A9, CDK5R1 | 0.210354463 |
| GOTERM_MF_DIRECT | GO:0004519~endonuclease activity | 4 | 0.040058117 | PMS2, ANG, RNASE3, RAF1 | 0.239820068 |
| GOTERM_MF_DIRECT | GO:0004702~receptor signaling protein serine/threonine kinase activity | 4 | 0.04201943 | TGFB2, MAP2K1, TGFBR1, TGFBR2 | 0.239820068 |
| GOTERM_MF_DIRECT | GO:0008431~vitamin E binding | 2 | 0.043086317 | SEC14L2, TTPA | 0.239820068 |
| GOTERM_MF_DIRECT | GO:0004705~JUN kinase activity | 2 | 0.043086317 | MAPK10, MAPK8 | 0.239820068 |
| GOTERM_MF_DIRECT | GO:0004886~9-cis retinoic acid receptor activity | 2 | 0.043086317 | RXRB, RXRA | 0.239820068 |
| GOTERM_MF_DIRECT | GO:0004517~nitric-oxide synthase activity | 2 | 0.043086317 | NOS2, NOS3 | 0.239820068 |
| GOTERM_MF_DIRECT | GO:0070026~nitric oxide binding | 2 | 0.043086317 | CBS, GSTP1 | 0.239820068 |
| GOTERM_MF_DIRECT | GO:0008238~exopeptidase activity | 2 | 0.043086317 | ACE, MME | 0.239820068 |
| GOTERM_MF_DIRECT | GO:0070644~vitamin D response element binding | 2 | 0.043086317 | RXRA, VDR | 0.239820068 |
| GOTERM_MF_DIRECT | GO:0097200~cysteine-type endopeptidase activity involved in execution phase of apoptosis | 2 | 0.043086317 | CASP7, CASP3 | 0.239820068 |
| GOTERM_MF_DIRECT | GO:0019003~GDP binding | 4 | 0.044028225 | ARF1, PCK1, RAB5A, RAN | 0.242772457 |
| GOTERM_MF_DIRECT | GO:0030742~GTP-dependent protein binding | 3 | 0.047301541 | CDC42, EEA1, RAC1 | 0.258406566 |
