# Supplementary material for: Total Flavonoids of Chuju Decrease Oxidative Stress and Cell Apoptosis in Ischemic Stroke Rats: Network and Experimental Analyses
Source: Front Neurosci. 2021 Dec 9;15:772401. doi: 10.3389/fnins.2021.772401 (PMC8695723; doi:10.3389/fnins.2021.772401)
Supplement: Supplementary file 4 [file Table_3.docx]

Supplementary Table 3 Functions of potential target genes based on GO biological process

| Category | Term | Count | PValue | Genes | FDR |
| --- | --- | --- | --- | --- | --- |
| GOTERM_BP_DIRECT | GO:0043401~steroid hormone mediated signaling pathway | 18 | 2.65E-18 | THRA, VDR, NR1H2, NR1I2, NR1H4, NR1H3, RORA, ESRRG, BMP7, ESR1, NR3C2, RXRB, RXRA, RARB, PGR, PPARG, PPARA, PPARD | 6.46E-15 |
| GOTERM_BP_DIRECT | GO:0038083~peptidyl-tyrosine autophosphorylation | 15 | 2.08E-16 | SYK, SRC, INSR, PTK2, IGF1R, ZAP70, HCK, ERBB4, LCK, KDR, BTK, ABL1, CSK, JAK2, JAK3 | 2.54E-13 |
| GOTERM_BP_DIRECT | GO:0046777~protein autophosphorylation | 22 | 7.49E-14 | GSK3B, SYK, DAPK1, PDPK1, SRC, INSR, EGFR, PTK2, IGF1R, HCK, ERBB4, KIT, KDR, ABL1, AKT1, CSK, TEK, JAK2, PRKACA, EPHB4, FGFR2, FGFR1 | 4.92E-11 |
| GOTERM_BP_DIRECT | GO:0018108~peptidyl-tyrosine phosphorylation | 21 | 8.05E-14 | MAP2K1, HSP90AA1, SRC, INSR, EGFR, PTK2, ZAP70, HCK, ERBB4, KIT, KDR, BTK, ABL1, TEK, JAK2, JAK3, MET, EPHB4, FGFR2, EPHA2, FGFR1 | 4.92E-11 |
| GOTERM_BP_DIRECT | GO:0006367~transcription initiation from RNA polymerase II promoter | 20 | 7.83E-13 | THRA, VDR, NR1H2, NR1I2, NR1H4, NR1H3, RORA, ESRRG, NR3C1, ESR1, ESR2, NR3C2, RXRB, AR, RXRA, RARB, PGR, PPARG, PPARA, PPARD | 3.82E-10 |
| GOTERM_BP_DIRECT | GO:0032869~cellular response to insulin stimulus | 15 | 4.80E-12 | PKLR, PARP1, PDPK1, STAT1, SRC, GSTP1, INSR, PDE3B, PIK3R1, GCK, APRT, AKT2, AKT1, PPARG, PCK1 | 1.95E-09 |
| GOTERM_BP_DIRECT | GO:0043066~negative regulation of apoptotic process | 30 | 2.37E-11 | GSK3B, SRC, GSTP1, GLO1, XIAP, PIK3R1, EGFR, IGF1R, MAPK8, ERBB4, CASP3, KDR, AKT1, NQO1, ANXA5, NR1H4, IGF1, MIF, SOD2, MMP9, IL2, PTK2, HCK, ALB, MDM2, RARB, TEK, RAF1, BCL2L1, PPARD | 8.27E-09 |
| GOTERM_BP_DIRECT | GO:0048015~phosphatidylinositol-mediated signaling | 16 | 3.54E-11 | PDPK1, NPR3, PTPN11, PIK3R1, IGF1, RHOA, EGFR, PIK3CG, IGF1R, ERBB4, LCK, KIT, NCS1, AKT1, FGFR2, FGFR1 | 1.08E-08 |
| GOTERM_BP_DIRECT | GO:0006508~proteolysis | 31 | 4.68E-11 | CFD, C1S, C1R, CTSS, DPP4, CASP7, PLAU, CASP3, CASP1, CTSG, LTA4H, CTSD, ELANE, CTSB, MMP7, MME, MMP1, MMP2, MMP3, F11, MMP8, F2, MMP9, BACE1, MMP12, ADAM17, MMP13, FOLH1, FAP, REN, CFB | 1.27E-08 |
| GOTERM_BP_DIRECT | GO:0001666~response to hypoxia | 19 | 6.60E-11 | TGFB2, AHCY, PKLR, NOS2, APAF1, MMP2, SOD2, TGFBR2, DPP4, F7, ADAM17, PLAU, CASP3, CASP1, HMOX1, ANG, TEK, RAF1, PPARA | 1.61E-08 |
| GOTERM_BP_DIRECT | GO:0042493~response to drug | 24 | 1.09E-10 | BCHE, TGFB2, HSP90AA1, MAOB, FECH, STAT1, SRC, ARG1, APOA2, SOD2, DUSP6, TGFBR2, ADAM17, FABP3, PNP, LCK, CASP3, MDM2, ABL1, LCN2, PMS2, PPARG, HADH, OTC | 2.37E-08 |
| GOTERM_BP_DIRECT | GO:0030168~platelet activation | 16 | 1.17E-10 | SYK, PDPK1, SRC, FGG, PTPN11, GP1BA, PIK3R1, F2, RHOA, PIK3CG, LCK, RAC2, AKT1, MAPK1, RAC1, RAF1 | 2.37E-08 |
| GOTERM_BP_DIRECT | GO:0043627~response to estrogen | 13 | 1.38E-10 | ARSA, HSP90AA1, GBA, APOA2, ESR1, TGFBR2, F7, CA2, CTNNA1, HMOX1, MAPK1, PPARG, TEK | 2.40E-08 |
| GOTERM_BP_DIRECT | GO:0014068~positive regulation of phosphatidylinositol 3-kinase signaling | 13 | 1.38E-10 | TGFB2, IGF1, F2, PTK2, SELP, ERBB4, CCL5, KIT, KDR, TEK, JAK2, FGFR1, PPARD | 2.40E-08 |
| GOTERM_BP_DIRECT | GO:0007165~signal transduction | 47 | 3.69E-10 | SRC, PDE3B, NR1I2, PIK3R1, NR3C1, EGFR, NR3C2, IGF1R, HINT1, IMPA1, PLAU, ERBB4, AKT2, CASP1, PDE4B, RAC2, AKT1, MAPK1, JAK2, HRAS, MAP2K1, HSP90AA1, CSNK2A1, DAPK1, VDR, PDE4D, FGG, ANXA5, NR1H4, IGF1, MAPK14, ESR1, TGFBR1, ESR2, MAPK10, AR, RHEB, KIT, RARB, PDE5A, PPARG, PGR, TEK, RAF1, S100A9, MET, RAN | 6.00E-08 |
| GOTERM_BP_DIRECT | GO:0007169~transmembrane receptor protein tyrosine kinase signaling pathway | 14 | 1.30E-09 | SYK, INSR, EGFR, IGF1R, ZAP70, HCK, ERBB4, LCK, KIT, KDR, BTK, CSK, TEK, MET | 1.98E-07 |
| GOTERM_BP_DIRECT | GO:0030335~positive regulation of cell migration | 18 | 1.59E-09 | F10, INSR, PIK3R1, IGF1, EGFR, TGFBR1, PTK2, IGF1R, F7, ADAM17, PLAU, CCL5, AKT2, KIT, KDR, MAPK1, JAK2, HRAS | 2.28E-07 |
| GOTERM_BP_DIRECT | GO:0018107~peptidyl-threonine phosphorylation | 10 | 2.81E-09 | GSK3B, MAPK8, PDPK1, CHEK1, MAPK1, AKT1, PRKACA, TGFBR1, CDK5R1, TGFBR2 | 3.82E-07 |
| GOTERM_BP_DIRECT | GO:0001525~angiogenesis | 19 | 4.56E-09 | GPI, TGFB2, SYK, NOS3, MMP2, PDE3B, RORA, MAPK14, PIK3CG, PTK2, TYMP, FAP, KDR, HMOX1, ANG, TEK, EPHB4, FGFR2, FGFR1 | 5.41E-07 |
| GOTERM_BP_DIRECT | GO:0008284~positive regulation of cell proliferation | 27 | 4.63E-09 | REG1A, EGFR, IGF1R, DPP4, ERBB4, KDR, RAC2, MAPK1, HRAS, TGFB2, CSNK2A1, INSR, IGF1, F2, TGFBR1, IL2, PTK2, TGFBR2, AR, HCK, ADAM17, KIT, MDM2, RARB, FGFR2, FGFR1, BCL2L1 | 5.41E-07 |
| GOTERM_BP_DIRECT | GO:0001934~positive regulation of protein phosphorylation | 15 | 4.65E-09 | INSR, F2, MMP9, EGFR, PTK2, ADAM17, RAP2A, ERBB4, AKT2, KDR, ABL1, AKT1, TEK, RAC1, HRAS | 5.41E-07 |
| GOTERM_BP_DIRECT | GO:0022617~extracellular matrix disassembly | 12 | 1.21E-08 | MMP12, MMP13, MMP7, MMP1, CMA1, MMP2, MMP3, CTSG, MMP8, MMP9, CTSS, ELANE | 1.35E-06 |
| GOTERM_BP_DIRECT | GO:0006468~protein phosphorylation | 26 | 1.32E-08 | GSK3B, PIK3R1, PIK3CG, MAPK8, AKT1, MAPK1, CSK, CTSG, JAK2, PRKACA, JAK3, TGFB2, SYK, CSNK2A1, DAPK1, PDPK1, TGFBR1, TGFBR2, MAPK10, ZAP70, HCK, CDK6, LCK, BTK, RAF1, FGFR1 | 1.40E-06 |
| GOTERM_BP_DIRECT | GO:0048661~positive regulation of smooth muscle cell proliferation | 11 | 1.39E-08 | STAT1, CCL5, AKR1B1, HMOX1, AKT1, HMGCR, IGF1, EGFR, FGFR2, ELANE, TGFBR2 | 1.42E-06 |
| GOTERM_BP_DIRECT | GO:0050900~leukocyte migration | 14 | 2.53E-08 | MMP1, SRC, PTPN11, PIK3R1, MIF, F2, SELE, MMP9, SELP, LCK, PDE4B, TEK, HRAS, PPIA | 2.47E-06 |
| GOTERM_BP_DIRECT | GO:0030574~collagen catabolic process | 11 | 2.66E-08 | MMP12, MMP13, MMP7, MMP1, MMP2, MMP3, MMP8, CTSD, MMP9, CTSS, CTSB | 2.50E-06 |
| GOTERM_BP_DIRECT | GO:0048013~ephrin receptor signaling pathway | 12 | 4.56E-08 | CDC42, SRC, MMP2, PTPN11, RAC1, HRAS, MMP9, EPHB4, PTK2, RHOA, EPHA2, CDK5R1 | 4.12E-06 |
| GOTERM_BP_DIRECT | GO:0010628~positive regulation of gene expression | 19 | 5.57E-08 | TGFB2, MAP2K1, VDR, EPHX2, GBA, NR1I2, MAPK14, TGFBR1, CDC42, AR, MAPK8, CDK6, KIT, MDM2, LCN2, HRAS, HSPA1B, HSPA1A, PPARD | 4.85E-06 |
| GOTERM_BP_DIRECT | GO:0071222~cellular response to lipopolysaccharide | 13 | 9.14E-08 | NOS2, SRC, ARG1, GSTP1, PDE4D, NR1H4, NR1H3, MAPK14, MAPK8, PDE4B, ABL1, LCN2, PPARD | 7.69E-06 |
| GOTERM_BP_DIRECT | GO:0007173~epidermal growth factor receptor signaling pathway | 10 | 1.04E-07 | ADAM17, PDPK1, SRC, ABL1, PTPN11, CSK, PIK3R1, HRAS, PTK2, EGFR | 8.48E-06 |
| GOTERM_BP_DIRECT | GO:0043406~positive regulation of MAP kinase activity | 10 | 1.66E-07 | SRC, KIT, CSK, PDE5A, MIF, HRAS, EGFR, ELANE, PIK3CG, FGFR1 | 1.31E-05 |
| GOTERM_BP_DIRECT | GO:0070374~positive regulation of ERK1 and ERK2 cascade | 15 | 2.76E-07 | MAP2K1, SRC, PLA2G2A, FGG, PTPN11, HMGCR, MIF, EGFR, ERBB4, CCL5, KDR, ABL1, TEK, HRAS, FGFR2 | 2.07E-05 |
| GOTERM_BP_DIRECT | GO:0018105~peptidyl-serine phosphorylation | 13 | 2.79E-07 | GSK3B, SYK, PDPK1, SRC, MAPK14, TGFBR1, TGFBR2, MAPK8, AKT2, AKT1, MAPK1, PRKACA, CDK5R1 | 2.07E-05 |
| GOTERM_BP_DIRECT | GO:0045087~innate immune response | 23 | 3.38E-07 | APCS, SYK, C1S, C1R, SRC, NR1H4, MIF, PIK3CG, PTK2, ZAP70, LGALS3, CD209, LCK, BTK, ABL1, LCN2, CSK, ANG, PPARG, PADI4, JAK2, JAK3, S100A9 | 2.43E-05 |
| GOTERM_BP_DIRECT | GO:0006809~nitric oxide biosynthetic process | 6 | 7.28E-07 | NQO1, ARG2, NOS2, NOS3, AKT1, RORA | 5.08E-05 |
| GOTERM_BP_DIRECT | GO:0030522~intracellular receptor signaling pathway | 8 | 1.08E-06 | AR, THRA, NR1I2, NR1H4, NR1H3, RORA, PPARA, PPARD | 7.31E-05 |
| GOTERM_BP_DIRECT | GO:0007584~response to nutrient | 10 | 1.21E-06 | NQO1, ARSA, AHCY, PKLR, APAF1, STAT1, TTPA, PPARG, HMGCR, TGFBR2 | 7.99E-05 |
| GOTERM_BP_DIRECT | GO:0051090~regulation of sequence-specific DNA binding transcription factor activity | 7 | 1.24E-06 | MAPK10, HCK, MAPK8, SYK, HMOX1, MAPK1, MAPK14 | 7.99E-05 |
| GOTERM_BP_DIRECT | GO:0000165~MAPK cascade | 17 | 1.49E-06 | MAP2K1, EGFR, IL2, PTK2, DUSP6, NDST1, ERBB4, CCL5, KIT, MAPK1, TEK, JAK2, RAF1, HRAS, JAK3, FGFR2, FGFR1 | 9.35E-05 |
| GOTERM_BP_DIRECT | GO:0031295~T cell costimulation | 10 | 1.90E-06 | CDC42, DPP4, PDPK1, SRC, LCK, AKT1, PTPN11, CSK, RAC1, PIK3R1 | 1.13E-04 |
| GOTERM_BP_DIRECT | GO:0014066~regulation of phosphatidylinositol 3-kinase signaling | 10 | 1.90E-06 | ERBB4, LCK, KIT, MAPK1, AKT1, PTPN11, PIK3R1, EGFR, FGFR2, FGFR1 | 1.13E-04 |
| GOTERM_BP_DIRECT | GO:0007507~heart development | 14 | 2.82E-06 | TGFB2, MAP2K1, PTPN11, SOD2, TGFBR1, TGFBR2, CASP7, RBP4, ERBB4, PPARG, TEK, RAF1, PPARA, PPARD | 1.64E-04 |
| GOTERM_BP_DIRECT | GO:0007596~blood coagulation | 14 | 3.00E-06 | SERPINA1, F10, F11, ANXA5, FGG, GP1BA, F2, CDC42, F7, PLAU, RAC1, JAK2, PRKACA, RAB5A | 1.70E-04 |
| GOTERM_BP_DIRECT | GO:0043542~endothelial cell migration | 7 | 3.18E-06 | DPP4, FAP, STAT1, NOS3, PTK2, RHOA, TGFBR1 | 1.76E-04 |
| GOTERM_BP_DIRECT | GO:0030307~positive regulation of cell growth | 10 | 3.56E-06 | CDC42, TGFB2, ADAM17, CSNK2A1, AKT1, F2, S100A9, EGFR, TGFBR1, IL2 | 1.93E-04 |
| GOTERM_BP_DIRECT | GO:0043552~positive regulation of phosphatidylinositol 3-kinase activity | 7 | 4.81E-06 | CDC42, ERBB4, SRC, KIT, TEK, RAC1, PTK2 | 2.55E-04 |
| GOTERM_BP_DIRECT | GO:0032496~response to lipopolysaccharide | 13 | 5.05E-06 | MAOB, SOD2, SELE, ADH5, SELP, ADAM17, CASP3, CASP1, CTSG, REN, JAK2, PCK1, ELANE | 2.62E-04 |
| GOTERM_BP_DIRECT | GO:0046854~phosphatidylinositol phosphorylation | 10 | 9.06E-06 | IMPA1, ERBB4, LCK, KIT, PTPN11, PIK3R1, EGFR, FGFR2, PIK3CG, FGFR1 | 4.61E-04 |
| GOTERM_BP_DIRECT | GO:0048010~vascular endothelial growth factor receptor signaling pathway | 9 | 9.51E-06 | CDC42, HSP90AA1, SRC, KDR, RAC1, PIK3R1, MAPK14, PTK2, RHOA | 4.74E-04 |
| GOTERM_BP_DIRECT | GO:0035902~response to immobilization stress | 6 | 1.05E-05 | GPI, TPH1, REN, PPARG, HNMT, SOD2 | 5.11E-04 |
| GOTERM_BP_DIRECT | GO:0033628~regulation of cell adhesion mediated by integrin | 5 | 1.32E-05 | PLAU, PTPN11, PTK2, PIK3CG, EPHA2 | 6.22E-04 |
| GOTERM_BP_DIRECT | GO:0002003~angiotensin maturation | 5 | 1.32E-05 | ACE, MME, CMA1, REN, CTSG | 6.22E-04 |
| GOTERM_BP_DIRECT | GO:0006749~glutathione metabolic process | 8 | 1.59E-05 | GSTZ1, G6PD, GSTM1, GSTO1, GLO1, GSTP1, GSR, SOD2 | 7.31E-04 |
| GOTERM_BP_DIRECT | GO:0038128~ERBB2 signaling pathway | 7 | 1.66E-05 | HSP90AA1, ERBB4, SRC, AKT1, PIK3R1, HRAS, EGFR | 7.48E-04 |
| GOTERM_BP_DIRECT | GO:0042127~regulation of cell proliferation | 13 | 1.73E-05 | NOS2, SRC, XIAP, PTK2, TGFBR2, PLAU, LCK, KIT, CHEK1, BTK, ABL1, CSK, JAK2 | 7.70E-04 |
| GOTERM_BP_DIRECT | GO:0050728~negative regulation of inflammatory response | 9 | 1.90E-05 | GBA, NR1H4, NR1H3, RORA, TEK, PPARA, IL2, ELANE, PPARD | 8.28E-04 |
| GOTERM_BP_DIRECT | GO:0048870~cell motility | 6 | 2.11E-05 | ADAM17, MAP2K1, RAC1, PTK2, TGFBR1, EPHA2 | 9.03E-04 |
| GOTERM_BP_DIRECT | GO:0045893~positive regulation of transcription, DNA-templated | 22 | 2.15E-05 | MAP2K1, STAT1, SRC, NR1H2, INSR, NR1I2, NR1H3, RORA, ESRRG, IGF1, BMP7, ESR1, TGFBR1, ESR2, SEC14L2, AR, ERBB4, MAPK1, PPARG, PPARA, RAN, PPARD | 9.04E-04 |
| GOTERM_BP_DIRECT | GO:0051897~positive regulation of protein kinase B signaling | 9 | 2.98E-05 | F7, F10, SRC, INSR, TEK, PTK2, EGFR, TGFBR1, PIK3CG | 0.001218933 |
| GOTERM_BP_DIRECT | GO:0045740~positive regulation of DNA replication | 7 | 3.00E-05 | CDC42, INSR, RAC1, IGF1, HRAS, EGFR, IGF1R | 0.001218933 |
| GOTERM_BP_DIRECT | GO:0010629~negative regulation of gene expression | 11 | 3.19E-05 | CDC42, TGFB2, MAP2K1, ACE, NOS2, GBA, AKT1, PGR, MIF, HRAS, ESR1 | 0.001275424 |
| GOTERM_BP_DIRECT | GO:0009636~response to toxic substance | 9 | 3.25E-05 | NQO1, MAOB, CCL5, EPHX2, GSTP1, TTPA, MDM2, MAPK1, CES1 | 0.001280698 |
| GOTERM_BP_DIRECT | GO:0045429~positive regulation of nitric oxide biosynthetic process | 7 | 3.44E-05 | HSP90AA1, INSR, AKT1, JAK2, SOD2, ESR1, EGFR | 0.001324687 |
| GOTERM_BP_DIRECT | GO:0050727~regulation of inflammatory response | 8 | 3.47E-05 | HCK, FABP4, CMA1, CASP1, XIAP, JAK2, SELE, ESR1 | 0.001324687 |
| GOTERM_BP_DIRECT | GO:0043101~purine-containing compound salvage | 5 | 3.88E-05 | MTAP, PNP, ADK, HPRT1, APRT | 0.001458162 |
| GOTERM_BP_DIRECT | GO:0008217~regulation of blood pressure | 8 | 4.26E-05 | ACE, NOS3, EPHX2, NPR3, HMOX1, REN, PPARG, SOD2 | 0.00157703 |
| GOTERM_BP_DIRECT | GO:0016477~cell migration | 12 | 4.40E-05 | GSK3B, TGFB2, ERBB4, PDPK1, ABL1, CSK, ANG, JAK2, JAK3, RHOA, EPHA2, FGFR1 | 0.0016038 |
| GOTERM_BP_DIRECT | GO:2000145~regulation of cell motility | 6 | 4.65E-05 | CDK6, ERBB4, ABL1, RAF1, RHOA, EGFR | 0.001666779 |
| GOTERM_BP_DIRECT | GO:0030593~neutrophil chemotaxis | 8 | 4.71E-05 | LGALS3, TGFB2, SYK, CCL5, PDE4D, PDE4B, S100A9, PIK3CG | 0.001666779 |
| GOTERM_BP_DIRECT | GO:0042026~protein refolding | 5 | 5.23E-05 | FKBP1A, HSPA8, HSP90AA1, HSPA1B, HSPA1A | 0.001799641 |
| GOTERM_BP_DIRECT | GO:0045725~positive regulation of glycogen biosynthetic process | 5 | 5.23E-05 | AKT2, INSR, AKT1, IGF1, GCK | 0.001799641 |
| GOTERM_BP_DIRECT | GO:0045909~positive regulation of vasodilation | 6 | 5.55E-05 | NOS2, NOS3, EPHX2, HMOX1, EGFR, PPARD | 0.001882354 |
| GOTERM_BP_DIRECT | GO:0044267~cellular protein metabolic process | 10 | 5.63E-05 | BACE1, APCS, MMP13, TTR, MMP1, MMP2, CTSG, IGF1, F2, LYZ | 0.001882539 |
| GOTERM_BP_DIRECT | GO:0010887~negative regulation of cholesterol storage | 4 | 5.87E-05 | NR1H2, NR1H3, PPARG, PPARA | 0.001935774 |
| GOTERM_BP_DIRECT | GO:0002250~adaptive immune response | 11 | 6.16E-05 | ZAP70, SYK, CD209, BTK, TAP1, CSK, JAK2, JAK3, CTSS, IL2, PIK3CG | 0.002004671 |
| GOTERM_BP_DIRECT | GO:0046326~positive regulation of glucose import | 6 | 6.58E-05 | AKT2, INSR, AKT1, IGF1, PIK3R1, MAPK14 | 0.002114735 |
| GOTERM_BP_DIRECT | GO:0035556~intracellular signal transduction | 18 | 8.80E-05 | GSK3B, SYK, DAPK1, PDPK1, SRC, MAPK14, TGFBR1, ZAP70, CD209, AKT2, KIT, BTK, AKT1, HMOX1, RAC1, JAK2, RAF1, JAK3 | 0.002789415 |
| GOTERM_BP_DIRECT | GO:0042523~positive regulation of tyrosine phosphorylation of Stat5 protein | 5 | 8.92E-05 | ERBB4, KIT, IGF1, JAK2, IL2 | 0.002791603 |
| GOTERM_BP_DIRECT | GO:0031663~lipopolysaccharide-mediated signaling pathway | 6 | 9.09E-05 | HCK, NOS3, CCL5, MAPK1, AKT1, MAPK14 | 0.002807571 |
| GOTERM_BP_DIRECT | GO:0045944~positive regulation of transcription from RNA polymerase II promoter | 31 | 9.33E-05 | GSK3B, THRA, NR1I2, RORA, PIK3R1, NR3C1, EGFR, RXRB, RXRA, AKT1, HRAS, PARP1, STAT1, VDR, NR1H2, NR1H4, NR1H3, ESRRG, IGF1, MAPK14, BMP7, ESR1, IL2, AR, RARB, PPARG, PGR, RAF1, PPARA, MET, FGFR2 | 0.00284605 |
| GOTERM_BP_DIRECT | GO:0038096~Fc-gamma receptor signaling pathway involved in phagocytosis | 10 | 9.97E-05 | CDC42, HCK, HSP90AA1, SYK, SRC, ABL1, MAPK1, RAC1, PIK3R1, PTK2 | 0.003003815 |
| GOTERM_BP_DIRECT | GO:0060687~regulation of branching involved in prostate gland morphogenesis | 4 | 1.02E-04 | RXRA, ESR1, BMP7, FGFR2 | 0.00302426 |
| GOTERM_BP_DIRECT | GO:0042593~glucose homeostasis | 9 | 1.12E-04 | GPI, RBP4, INSR, NR1H4, AKT1, PTPN11, PPARG, PCK1, GCK | 0.003291933 |
| GOTERM_BP_DIRECT | GO:0048384~retinoic acid receptor signaling pathway | 5 | 1.13E-04 | RXRB, RXRA, NR1H2, RARB, ESRRG | 0.003294933 |
| GOTERM_BP_DIRECT | GO:0006954~inflammatory response | 17 | 1.40E-04 | SYK, EPHX2, NR1H4, MIF, LYZ, SELE, PIK3CG, SELP, NDST1, ZAP70, HCK, CCL5, KIT, AKT1, RAC1, S100A9, EPHA2 | 0.004022322 |
| GOTERM_BP_DIRECT | GO:0045471~response to ethanol | 9 | 1.47E-04 | NQO1, ARSA, G6PD, RBP4, MAOB, FECH, GSTP1, HMGCR, IL2 | 0.004170635 |
| GOTERM_BP_DIRECT | GO:0007568~aging | 11 | 1.53E-04 | NQO1, CASP7, MMP7, APAF1, ARG1, PDE4D, CTNNA1, AKT1, HMGCR, PCK1, TGFBR2 | 0.004282878 |
| GOTERM_BP_DIRECT | GO:0048762~mesenchymal cell differentiation | 4 | 1.61E-04 | BMP7, TGFBR1, FGFR2, FGFR1 | 0.004400351 |
| GOTERM_BP_DIRECT | GO:0042060~wound healing | 8 | 1.62E-04 | TGFB2, CASP3, RAF1, PPARA, EGFR, TGFBR1, TGFBR2, PPARD | 0.004400351 |
| GOTERM_BP_DIRECT | GO:0050714~positive regulation of protein secretion | 6 | 1.62E-04 | TGFB2, ARF1, FGG, ANG, IGF1, PPIA | 0.004400351 |
| GOTERM_BP_DIRECT | GO:0000187~activation of MAPK activity | 9 | 1.68E-04 | MAPK10, MAP2K1, INSR, KIT, MAPK1, PTPN11, IGF1, MAPK14, DUSP6 | 0.004494644 |
| GOTERM_BP_DIRECT | GO:0043410~positive regulation of MAPK cascade | 8 | 1.75E-04 | AR, INSR, KIT, KDR, IGF1, HRAS, FGFR2, FGFR1 | 0.004641274 |
| GOTERM_BP_DIRECT | GO:2001237~negative regulation of extrinsic apoptotic signaling pathway | 6 | 2.11E-04 | LGALS3, AR, SRC, GSTP1, IGF1, TGFBR1 | 0.005537351 |
| GOTERM_BP_DIRECT | GO:0008285~negative regulation of cell proliferation | 17 | 2.32E-04 | BCHE, TGFB2, MAP2K1, NOS3, VDR, SOD2, BMP7, AR, FABP3, CDK6, ERBB4, FABP7, RARB, CSK, JAK2, RAF1, HRAS | 0.006034223 |
| GOTERM_BP_DIRECT | GO:0060020~Bergmann glial cell differentiation | 4 | 2.39E-04 | MAP2K1, ABL1, MAPK1, PTPN11 | 0.006067365 |
| GOTERM_BP_DIRECT | GO:2000188~regulation of cholesterol homeostasis | 4 | 2.39E-04 | NR1H2, NR1H4, NR1H3, RORA | 0.006067365 |
| GOTERM_BP_DIRECT | GO:0007264~small GTPase mediated signal transduction | 13 | 2.63E-04 | ARF1, RND3, RHOA, RAB11A, CDC42, RAP2A, RHEB, RAC2, HMOX1, RAC1, HRAS, RAB5A, RAN | 0.006630574 |
| GOTERM_BP_DIRECT | GO:0043124~negative regulation of I-kappaB kinase/NF-kappaB signaling | 6 | 2.70E-04 | STAT1, GSTP1, NR1H4, ABL1, RORA, ESR1 | 0.006727861 |
| GOTERM_BP_DIRECT | GO:0008283~cell proliferation | 16 | 3.11E-04 | TGFB2, SYK, SRC, IGF1, MIF, EGFR, AR, ERBB4, AKT1, RAC1, RAF1, HRAS, MET, CDK5R1, BCL2L1, PPARD | 0.007668103 |
| GOTERM_BP_DIRECT | GO:0051156~glucose 6-phosphate metabolic process | 4 | 3.37E-04 | GPI, G6PD, GCK, HK1 | 0.008231742 |
| GOTERM_BP_DIRECT | GO:0043525~positive regulation of neuron apoptotic process | 6 | 3.81E-04 | CDC42, NQO1, TGFB2, CASP3, ABL1, CDK5R1 | 0.009215456 |
| GOTERM_BP_DIRECT | GO:0007179~transforming growth factor beta receptor signaling pathway | 8 | 3.85E-04 | FKBP1A, TGFB2, PARP1, SRC, PTK2, RHOA, TGFBR1, TGFBR2 | 0.009215456 |
| GOTERM_BP_DIRECT | GO:0006006~glucose metabolic process | 7 | 4.21E-04 | G6PD, FABP5, AKT2, AKT1, PCK1, MAPK14, PPARD | 0.009899466 |
| GOTERM_BP_DIRECT | GO:0055114~oxidation-reduction process | 21 | 4.22E-04 | NQO1, G6PD, TPH1, MAOB, NOS2, GSTO1, NOS3, GSR, AKR1B1, HMGCR, SOD2, CYP19A1, CRYZ, DHFR, HSD11B1, CYP2C9, CBS, PAH, PNPO, BLVRB, ACADM | 0.009899466 |
| GOTERM_BP_DIRECT | GO:0007159~leukocyte cell-cell adhesion | 5 | 4.33E-04 | SELP, SYK, CD209, CCL5, SELE | 0.009965186 |
| GOTERM_BP_DIRECT | GO:0042327~positive regulation of phosphorylation | 5 | 4.33E-04 | AR, CCL5, ANG, MIF, EGFR | 0.009965186 |
| GOTERM_BP_DIRECT | GO:0008584~male gonad development | 8 | 4.39E-04 | TGFB2, INSR, KIT, CTNNA1, REN, ESR1, TGFBR1, BCL2L1 | 0.010015754 |
| GOTERM_BP_DIRECT | GO:0043154~negative regulation of cysteine-type endopeptidase activity involved in apoptotic process | 7 | 4.94E-04 | GPI, CSNK2A1, SRC, MDM2, XIAP, AKT1, RAF1 | 0.011119121 |
| GOTERM_BP_DIRECT | GO:0071456~cellular response to hypoxia | 8 | 4.99E-04 | SRC, MDM2, HMOX1, AKT1, RORA, PCK1, BMP7, PPARD | 0.011119121 |
| GOTERM_BP_DIRECT | GO:0032148~activation of protein kinase B activity | 5 | 5.06E-04 | PDPK1, SRC, INSR, ANG, IGF1 | 0.011119121 |
| GOTERM_BP_DIRECT | GO:0061621~canonical glycolysis | 5 | 5.06E-04 | GPI, TPI1, PKLR, GCK, HK1 | 0.011119121 |
| GOTERM_BP_DIRECT | GO:0071260~cellular response to mechanical stimulus | 7 | 5.76E-04 | MAPK8, MMP7, CHEK1, CASP1, AKT1, RAC1, EGFR | 0.012524106 |
| GOTERM_BP_DIRECT | GO:0008630~intrinsic apoptotic signaling pathway in response to DNA damage | 6 | 5.80E-04 | ABL1, HMOX1, PIK3R1, SOD2, EPHA2, BCL2L1 | 0.012524106 |
| GOTERM_BP_DIRECT | GO:0009268~response to pH | 4 | 6.05E-04 | ARSA, CA2, TTPA, GBA | 0.012956119 |
| GOTERM_BP_DIRECT | GO:0033688~regulation of osteoblast proliferation | 3 | 6.25E-04 | NPR3, RHOA, FGFR2 | 0.013262184 |
| GOTERM_BP_DIRECT | GO:0006915~apoptotic process | 20 | 6.47E-04 | CSNK2A1, APAF1, DAPK1, STAT1, XIAP, MAPK14, NR3C1, TGFBR1, TGFBR2, CASP7, CASP3, AKT2, CASP1, LCN2, MAPK1, JAK2, RAF1, S100A9, FGFR2, PPARD | 0.013611762 |
| GOTERM_BP_DIRECT | GO:0050729~positive regulation of inflammatory response | 7 | 6.68E-04 | FABP4, CCL5, PLA2G2A, JAK2, S100A9, EGFR, IL2 | 0.013945217 |
| GOTERM_BP_DIRECT | GO:0048662~negative regulation of smooth muscle cell proliferation | 5 | 7.76E-04 | NPR3, HMOX1, ANG, PPARG, PPARD | 0.015698782 |
| GOTERM_BP_DIRECT | GO:0050435~beta-amyloid metabolic process | 4 | 7.78E-04 | BACE1, ACE, MME, REN | 0.015698782 |
| GOTERM_BP_DIRECT | GO:0010745~negative regulation of macrophage derived foam cell differentiation | 4 | 7.78E-04 | NR1H2, NR1H3, PPARG, PPARA | 0.015698782 |
| GOTERM_BP_DIRECT | GO:2001275~positive regulation of glucose import in response to insulin stimulus | 4 | 7.78E-04 | AKT2, NR1H4, PTPN11, PIK3R1 | 0.015698782 |
| GOTERM_BP_DIRECT | GO:0002223~stimulatory C-type lectin receptor signaling pathway | 8 | 8.53E-04 | SYK, PDPK1, CD209, SRC, ICAM2, RAF1, PRKACA, HRAS | 0.017072976 |
| GOTERM_BP_DIRECT | GO:0001501~skeletal system development | 9 | 8.86E-04 | TGFB2, VDR, NPR3, IGF1, MMP9, BMP7, TGFBR1, EPHA2, FGFR1 | 0.017579482 |
| GOTERM_BP_DIRECT | GO:0006805~xenobiotic metabolic process | 7 | 9.50E-04 | NQO1, CYP2C9, EPHX2, GSTP1, NR1I2, RORA, CES1 | 0.018694006 |
| GOTERM_BP_DIRECT | GO:0060065~uterus development | 4 | 9.80E-04 | TGFB2, RBP4, SRC, ESR1 | 0.018982248 |
| GOTERM_BP_DIRECT | GO:0048009~insulin-like growth factor receptor signaling pathway | 4 | 9.80E-04 | AKT1, IGF1, PIK3R1, IGF1R | 0.018982248 |
| GOTERM_BP_DIRECT | GO:0048146~positive regulation of fibroblast proliferation | 6 | 0.001100806 | CDK6, ABL1, IGF1, MIF, ESR1, EGFR | 0.020992723 |
| GOTERM_BP_DIRECT | GO:0050853~B cell receptor signaling pathway | 6 | 0.001100806 | ZAP70, SYK, LCK, BTK, ABL1, MAPK1 | 0.020992723 |
| GOTERM_BP_DIRECT | GO:0003007~heart morphogenesis | 5 | 0.001135957 | FKBP1A, TGFB2, INSR, EPHB4, PTK2 | 0.021495127 |
| GOTERM_BP_DIRECT | GO:1904707~positive regulation of vascular smooth muscle cell proliferation | 4 | 0.0012117 | MMP2, MDM2, JAK2, MMP9 | 0.022578324 |
| GOTERM_BP_DIRECT | GO:0060397~JAK-STAT cascade involved in growth hormone signaling pathway | 4 | 0.0012117 | PTPN1, MAPK1, JAK2, JAK3 | 0.022578324 |
| GOTERM_BP_DIRECT | GO:0006166~purine ribonucleoside salvage | 3 | 0.001237618 | MTAP, ADK, HPRT1 | 0.022886564 |
| GOTERM_BP_DIRECT | GO:1902042~negative regulation of extrinsic apoptotic signaling pathway via death domain receptors | 5 | 0.001278044 | DAPK1, NOS3, FGG, HMOX1, RAF1 | 0.023281379 |
| GOTERM_BP_DIRECT | GO:0015758~glucose transport | 5 | 0.001278044 | FABP5, AKT1, GCK, HK1, PPARD | 0.023281379 |
| GOTERM_BP_DIRECT | GO:0006919~activation of cysteine-type endopeptidase activity involved in apoptotic process | 7 | 0.001314111 | APAF1, LCK, CASP3, CASP1, PPARG, JAK2, S100A9 | 0.023761073 |
| GOTERM_BP_DIRECT | GO:0097192~extrinsic apoptotic signaling pathway in absence of ligand | 5 | 0.001432101 | GSK3B, CASP3, LCN2, IL2, BCL2L1 | 0.025516482 |
| GOTERM_BP_DIRECT | GO:0001837~epithelial to mesenchymal transition | 5 | 0.001432101 | GSK3B, TGFB2, BMP7, TGFBR1, FGFR2 | 0.025516482 |
| GOTERM_BP_DIRECT | GO:0050852~T cell receptor signaling pathway | 9 | 0.001457101 | ZAP70, PDPK1, LCK, PDE4D, PDE4B, MAPK1, CSK, PIK3R1, HRAS | 0.025542218 |
| GOTERM_BP_DIRECT | GO:0016032~viral process | 13 | 0.001474617 | HSPA8, SYK, MMP1, APOA2, PIK3R1, RHOA, HCK, LCK, MDM2, KDR, MAPK1, RAN, EPHA2 | 0.025542218 |
| GOTERM_BP_DIRECT | GO:0048011~neurotrophin TRK receptor signaling pathway | 4 | 0.001475401 | SRC, CASP3, PTPN11, RAF1 | 0.025542218 |
| GOTERM_BP_DIRECT | GO:0031396~regulation of protein ubiquitination | 4 | 0.001475401 | HSP90AA1, TGFBR1, HSPA1B, HSPA1A | 0.025542218 |
| GOTERM_BP_DIRECT | GO:0060749~mammary gland alveolus development | 4 | 0.001772443 | AR, TPH1, ERBB4, ESR1 | 0.029935033 |
| GOTERM_BP_DIRECT | GO:0043407~negative regulation of MAP kinase activity | 5 | 0.001778197 | PTPN1, GSTP1, GBA, HMGCR, BMP7 | 0.029935033 |
| GOTERM_BP_DIRECT | GO:0007611~learning or memory | 5 | 0.001778197 | GPI, GM2A, THRA, CASP3, EGFR | 0.029935033 |
| GOTERM_BP_DIRECT | GO:0009409~response to cold | 5 | 0.001778197 | HSP90AA1, THRA, PPARG, ACADM, SOD2 | 0.029935033 |
| GOTERM_BP_DIRECT | GO:0034332~adherens junction organization | 5 | 0.001971265 | CDC42, SRC, CTNNA1, CSK, ANG | 0.032733731 |
| GOTERM_BP_DIRECT | GO:2001240~negative regulation of extrinsic apoptotic signaling pathway in absence of ligand | 5 | 0.001971265 | CTNNA1, AKT1, HSPA1B, BCL2L1, HSPA1A | 0.032733731 |
| GOTERM_BP_DIRECT | GO:0006935~chemotaxis | 8 | 0.002040771 | MAP2K1, PLAU, CCL5, RAC2, MAPK1, MAPK14, HRAS, TYMP | 0.033245174 |
| GOTERM_BP_DIRECT | GO:0060336~negative regulation of interferon-gamma-mediated signaling pathway | 3 | 0.002042923 | NR1H2, NR1H3, PPARG | 0.033245174 |
| GOTERM_BP_DIRECT | GO:1905007~positive regulation of epithelial to mesenchymal transition involved in endocardial cushion formation | 3 | 0.002042923 | TGFB2, TGFBR1, TGFBR2 | 0.033245174 |
| GOTERM_BP_DIRECT | GO:0007597~blood coagulation, intrinsic pathway | 4 | 0.002104254 | F10, F11, GP1BA, F2 | 0.033857651 |
| GOTERM_BP_DIRECT | GO:0032355~response to estradiol | 7 | 0.002108301 | NQO1, GPI, F7, CASP3, GSTP1, ESR1, BMP7 | 0.033857651 |
| GOTERM_BP_DIRECT | GO:0001657~ureteric bud development | 5 | 0.002178359 | ARG2, RARB, BMP7, FGFR2, FGFR1 | 0.034754081 |
| GOTERM_BP_DIRECT | GO:0042632~cholesterol homeostasis | 6 | 0.002360157 | FABP3, FABP4, NR1H2, EPHX2, APOA2, NR1H3 | 0.037410027 |
| GOTERM_BP_DIRECT | GO:0051384~response to glucocorticoid | 6 | 0.002527074 | BCHE, CASP3, GBA, APOA2, HNMT, AGXT | 0.039797339 |
| GOTERM_BP_DIRECT | GO:0030163~protein catabolic process | 5 | 0.002888811 | BACE1, AKT1, REN, CTSD, ELANE | 0.04520249 |
| GOTERM_BP_DIRECT | GO:0030036~actin cytoskeleton organization | 8 | 0.002919853 | CDC42, PDPK1, RAC2, ABL1, RAC1, RND3, RHOA, RAN | 0.0453972 |
| GOTERM_BP_DIRECT | GO:0060440~trachea formation | 3 | 0.003035043 | MAP2K1, MAPK1, TGFBR2 | 0.046889497 |
| GOTERM_BP_DIRECT | GO:0007566~embryo implantation | 5 | 0.003156997 | RXRA, MMP2, MMP9, TGFBR2, PPARD | 0.048466857 |
| GOTERM_BP_DIRECT | GO:0001938~positive regulation of endothelial cell proliferation | 6 | 0.003281183 | ARG1, KDR, AKT1, ANG, TEK, TGFBR1 | 0.049738663 |
| GOTERM_BP_DIRECT | GO:1900182~positive regulation of protein localization to nucleus | 4 | 0.003321345 | TGFB2, SRC, AKT1, F2 | 0.049738663 |
| GOTERM_BP_DIRECT | GO:0042730~fibrinolysis | 4 | 0.003321345 | PLAU, FGG, GP1BA, F2 | 0.049738663 |
| GOTERM_BP_DIRECT | GO:0045861~negative regulation of proteolysis | 4 | 0.003321345 | NR1H2, NR1H3, AKT1, F2 | 0.049738663 |
| GOTERM_BP_DIRECT | GO:0048538~thymus development | 5 | 0.003441675 | MAP2K1, ABL1, MAPK1, RAF1, TGFBR1 | 0.051226389 |
| GOTERM_BP_DIRECT | GO:0098869~cellular oxidant detoxification | 6 | 0.003492522 | GSTZ1, GSTO1, GSTP1, ALB, GSR, S100A9 | 0.051356904 |
| GOTERM_BP_DIRECT | GO:0030855~epithelial cell differentiation | 6 | 0.003492522 | LGALS3, PPARG, BMP7, FGFR2, CTSB, CES1 | 0.051356904 |
| GOTERM_BP_DIRECT | GO:0023014~signal transduction by protein phosphorylation | 5 | 0.003743318 | TGFB2, INSR, KDR, TGFBR1, TGFBR2 | 0.054314126 |
| GOTERM_BP_DIRECT | GO:0070207~protein homotrimerization | 4 | 0.003804881 | ARG1, LCN2, MIF, OTC | 0.054314126 |
| GOTERM_BP_DIRECT | GO:1901687~glutathione derivative biosynthetic process | 4 | 0.003804881 | GSTZ1, GSTM1, GSTO1, GSTP1 | 0.054314126 |
| GOTERM_BP_DIRECT | GO:0051000~positive regulation of nitric-oxide synthase activity | 4 | 0.003804881 | DHFR, NPR3, AKT1, ESR1 | 0.054314126 |
| GOTERM_BP_DIRECT | GO:0060045~positive regulation of cardiac muscle cell proliferation | 4 | 0.003804881 | ERBB4, MAPK14, FGFR2, FGFR1 | 0.054314126 |
| GOTERM_BP_DIRECT | GO:0002576~platelet degranulation | 7 | 0.003917608 | SELP, CFD, TGFB2, SERPINA1, ALB, FGG, IGF1 | 0.055598149 |
| GOTERM_BP_DIRECT | GO:0016485~protein processing | 6 | 0.003944254 | F7, CASP7, CMA1, CASP1, CTSG, CTSS | 0.05565274 |
| GOTERM_BP_DIRECT | GO:0042981~regulation of apoptotic process | 10 | 0.004043341 | APAF1, STAT1, DAPK1, CASP1, JAK2, RAF1, ESR1, BMP7, CTSB, BCL2L1 | 0.056664583 |
| GOTERM_BP_DIRECT | GO:0006687~glycosphingolipid metabolic process | 5 | 0.004062393 | ARSA, STS, GM2A, GBA, KIT | 0.056664583 |
| GOTERM_BP_DIRECT | GO:0042178~xenobiotic catabolic process | 3 | 0.004208423 | GSTM1, GSTO1, CRYZ | 0.05771214 |
| GOTERM_BP_DIRECT | GO:0045822~negative regulation of heart contraction | 3 | 0.004208423 | PDE4D, JAK2, IL2 | 0.05771214 |
| GOTERM_BP_DIRECT | GO:0009072~aromatic amino acid family metabolic process | 3 | 0.004208423 | GSTZ1, TPH1, PAH | 0.05771214 |
| GOTERM_BP_DIRECT | GO:0003148~outflow tract septum morphogenesis | 4 | 0.004329128 | TGFB2, RARB, FGFR2, TGFBR2 | 0.059035766 |
| GOTERM_BP_DIRECT | GO:0051591~response to cAMP | 5 | 0.004399361 | PKLR, STAT1, REN, TEK, AGXT | 0.05966022 |
| GOTERM_BP_DIRECT | GO:0043065~positive regulation of apoptotic process | 12 | 0.004560222 | MAPK8, APAF1, ERBB4, SRC, ABL1, HMOX1, RARB, AKT1, RAC1, BMP7, DUSP6, BCL2L1 | 0.061293854 |
| GOTERM_BP_DIRECT | GO:0038095~Fc-epsilon receptor signaling pathway | 9 | 0.004570046 | MAPK10, MAPK8, SYK, PDPK1, BTK, MAPK1, RAC1, PIK3R1, HRAS | 0.061293854 |
| GOTERM_BP_DIRECT | GO:1900034~regulation of cellular response to heat | 6 | 0.004698065 | HSPA8, GSK3B, HSP90AA1, MAPK1, HSPA1B, HSPA1A | 0.06239867 |
| GOTERM_BP_DIRECT | GO:0031532~actin cytoskeleton reorganization | 5 | 0.004754671 | PTPN1, RAP2A, KIT, S100A9, RHOA | 0.06239867 |
| GOTERM_BP_DIRECT | GO:0031100~organ regeneration | 5 | 0.004754671 | F7, GSTP1, APOA2, PPARG, TGFBR2 | 0.06239867 |
| GOTERM_BP_DIRECT | GO:0045600~positive regulation of fat cell differentiation | 5 | 0.004754671 | TPH1, SULT1E1, AKT1, PPARG, PPARD | 0.06239867 |
| GOTERM_BP_DIRECT | GO:0051149~positive regulation of muscle cell differentiation | 4 | 0.004895051 | CDC42, CTNNA1, ABL1, MAPK14 | 0.063897436 |
| GOTERM_BP_DIRECT | GO:0030324~lung development | 6 | 0.004970608 | HSD11B1, RBP4, NOS3, ARG1, EGFR, FGFR2 | 0.064538583 |
| GOTERM_BP_DIRECT | GO:0009408~response to heat | 5 | 0.005128768 | HSP90AA1, PKLR, NOS3, AKT1, IGF1 | 0.065855212 |
| GOTERM_BP_DIRECT | GO:0030878~thyroid gland development | 4 | 0.005503549 | MAP2K1, THRA, MAPK1, RAF1 | 0.065855212 |
| GOTERM_BP_DIRECT | GO:0070372~regulation of ERK1 and ERK2 cascade | 4 | 0.005503549 | SYK, GSTP1, FGFR2, EPHA2 | 0.065855212 |
| GOTERM_BP_DIRECT | GO:0019433~triglyceride catabolic process | 4 | 0.005503549 | FABP3, FABP4, FABP5, FABP7 | 0.065855212 |
| GOTERM_BP_DIRECT | GO:0042752~regulation of circadian rhythm | 5 | 0.005522085 | MAPK10, MAPK8, NR1H3, PPARG, PPARA | 0.065855212 |
| GOTERM_BP_DIRECT | GO:0036092~phosphatidylinositol-3-phosphate biosynthetic process | 5 | 0.005522085 | PTPN11, PIK3R1, FGFR2, PIK3CG, FGFR1 | 0.065855212 |
| GOTERM_BP_DIRECT | GO:0008286~insulin receptor signaling pathway | 6 | 0.00554901 | PTPN1, AKT2, INSR, AKT1, PIK3R1, IGF1R | 0.065855212 |
| GOTERM_BP_DIRECT | GO:0042129~regulation of T cell proliferation | 3 | 0.005557629 | LGALS3, CD209, RAC2 | 0.065855212 |
| GOTERM_BP_DIRECT | GO:0002674~negative regulation of acute inflammatory response | 3 | 0.005557629 | APCS, GSTP1, PPARG | 0.065855212 |
| GOTERM_BP_DIRECT | GO:0032872~regulation of stress-activated MAPK cascade | 3 | 0.005557629 | MAP2K1, GSTP1, MAPK1 | 0.065855212 |
| GOTERM_BP_DIRECT | GO:0031659~positive regulation of cyclin-dependent protein serine/threonine kinase activity involved in G1/S transition of mitotic cell cycle | 3 | 0.005557629 | ADAM17, AKT1, EGFR | 0.065855212 |
| GOTERM_BP_DIRECT | GO:0010518~positive regulation of phospholipase activity | 3 | 0.005557629 | PDPK1, FGFR2, FGFR1 | 0.065855212 |
| GOTERM_BP_DIRECT | GO:0051597~response to methylmercury | 3 | 0.005557629 | ARSA, FECH, ARG1 | 0.065855212 |
| GOTERM_BP_DIRECT | GO:0006527~arginine catabolic process | 3 | 0.005557629 | NOS2, NOS3, ARG1 | 0.065855212 |
| GOTERM_BP_DIRECT | GO:0010269~response to selenium ion | 3 | 0.005557629 | MAOB, ARG1, SOD2 | 0.065855212 |
| GOTERM_BP_DIRECT | GO:0070723~response to cholesterol | 3 | 0.005557629 | F7, TGFBR1, TGFBR2 | 0.065855212 |
| GOTERM_BP_DIRECT | GO:0060396~growth hormone receptor signaling pathway | 3 | 0.005557629 | PIK3R1, JAK2, PTK2 | 0.065855212 |
| GOTERM_BP_DIRECT | GO:2000641~regulation of early endosome to late endosome transport | 3 | 0.005557629 | MAP2K1, SRC, MAPK1 | 0.065855212 |
| GOTERM_BP_DIRECT | GO:0001701~in utero embryonic development | 9 | 0.006120493 | GPI, AR, RXRA, NOS3, TGFBR1, FGFR2, FGFR1, BCL2L1, TGFBR2 | 0.071418142 |
| GOTERM_BP_DIRECT | GO:0030041~actin filament polymerization | 4 | 0.006155452 | ABL1, ANG, RAC1, JAK2 | 0.071418142 |
| GOTERM_BP_DIRECT | GO:0050999~regulation of nitric-oxide synthase activity | 4 | 0.006155452 | HSP90AA1, NOS3, AKT1, EGFR | 0.071418142 |
| GOTERM_BP_DIRECT | GO:0002053~positive regulation of mesenchymal cell proliferation | 4 | 0.006155452 | STAT1, FGFR2, FGFR1, TGFBR2 | 0.071418142 |
| GOTERM_BP_DIRECT | GO:0035264~multicellular organism growth | 6 | 0.006173383 | CDC42, AR, PDE4D, RARB, PTPN11, FGFR2 | 0.071418142 |
| GOTERM_BP_DIRECT | GO:0042542~response to hydrogen peroxide | 5 | 0.00636807 | STAT1, SRC, CASP3, HMOX1, SOD2 | 0.073322924 |
| GOTERM_BP_DIRECT | GO:0050731~positive regulation of peptidyl-tyrosine phosphorylation | 6 | 0.00684556 | SYK, SRC, ABL1, IGF1, MIF, JAK2 | 0.077818937 |
| GOTERM_BP_DIRECT | GO:0014065~phosphatidylinositol 3-kinase signaling | 4 | 0.00685153 | IGF1, PIK3R1, PIK3CG, IGF1R | 0.077818937 |
| GOTERM_BP_DIRECT | GO:0006972~hyperosmotic response | 3 | 0.007077347 | PDPK1, RAC1, HNMT | 0.077818937 |
| GOTERM_BP_DIRECT | GO:0010863~positive regulation of phospholipase C activity | 3 | 0.007077347 | KIT, ESR1, FGFR1 | 0.077818937 |
| GOTERM_BP_DIRECT | GO:0010907~positive regulation of glucose metabolic process | 3 | 0.007077347 | SRC, AKT2, AKT1 | 0.077818937 |
| GOTERM_BP_DIRECT | GO:0050778~positive regulation of immune response | 3 | 0.007077347 | TGFB2, CTSG, ELANE | 0.077818937 |
| GOTERM_BP_DIRECT | GO:0046638~positive regulation of alpha-beta T cell differentiation | 3 | 0.007077347 | ZAP70, PNP, SYK | 0.077818937 |
| GOTERM_BP_DIRECT | GO:0039694~viral RNA genome replication | 3 | 0.007077347 | CDC42, EEA1, RAB5A | 0.077818937 |
| GOTERM_BP_DIRECT | GO:0007167~enzyme linked receptor protein signaling pathway | 3 | 0.007077347 | SYK, JAK2, JAK3 | 0.077818937 |
| GOTERM_BP_DIRECT | GO:0008635~activation of cysteine-type endopeptidase activity involved in apoptotic process by cytochrome c | 3 | 0.007077347 | CASP7, APAF1, CASP3 | 0.077818937 |
| GOTERM_BP_DIRECT | GO:2000352~negative regulation of endothelial cell apoptotic process | 4 | 0.007592486 | FGG, ABL1, KDR, TEK | 0.082737766 |
| GOTERM_BP_DIRECT | GO:0050690~regulation of defense response to virus by virus | 4 | 0.007592486 | HCK, ARF1, LCK, RAC1 | 0.082737766 |
| GOTERM_BP_DIRECT | GO:0048511~rhythmic process | 5 | 0.007791528 | MAPK10, MAPK8, CSNK2A1, PPARG, CDK5R1 | 0.084529423 |
| GOTERM_BP_DIRECT | GO:0050830~defense response to Gram-positive bacterium | 6 | 0.007947395 | ADAM17, PLA2G2A, ANG, RNASE3, LYZ, EPHA2 | 0.085838898 |
| GOTERM_BP_DIRECT | GO:0001822~kidney development | 6 | 0.008340507 | TGFB2, ACE, MME, CA2, REN, TGFBR1 | 0.089688011 |
| GOTERM_BP_DIRECT | GO:0033160~positive regulation of protein import into nucleus, translocation | 3 | 0.008762377 | HSP90AA1, IGF1, JAK2 | 0.093401578 |
| GOTERM_BP_DIRECT | GO:0003149~membranous septum morphogenesis | 3 | 0.008762377 | TGFB2, FGFR2, TGFBR2 | 0.093401578 |
| GOTERM_BP_DIRECT | GO:0050680~negative regulation of epithelial cell proliferation | 5 | 0.008848 | AR, TGFB2, CDK6, FGFR2, PPARD | 0.093904211 |
| GOTERM_BP_DIRECT | GO:0006730~one-carbon metabolic process | 4 | 0.009211564 | DHFR, CA1, AHCY, CA2 | 0.096503978 |
| GOTERM_BP_DIRECT | GO:0090004~positive regulation of establishment of protein localization to plasma membrane | 4 | 0.009211564 | PDPK1, AKT1, PIK3R1, EPHA2 | 0.096503978 |
| GOTERM_BP_DIRECT | GO:0030217~T cell differentiation | 4 | 0.009211564 | ZAP70, LCK, KIT, IL2 | 0.096503978 |
| GOTERM_BP_DIRECT | GO:0070301~cellular response to hydrogen peroxide | 5 | 0.009409586 | ARG1, MDM2, ABL1, LCN2, AKR1B1 | 0.098157266 |
| GOTERM_BP_DIRECT | GO:0070373~negative regulation of ERK1 and ERK2 cascade | 5 | 0.00999387 | PTPN1, GSTP1, ABL1, CSK, DUSP6 | 0.103808671 |
| GOTERM_BP_DIRECT | GO:0030901~midbrain development | 4 | 0.010090804 | NDST1, CMA1, FGFR2, FGFR1 | 0.104371409 |
| GOTERM_BP_DIRECT | GO:0060445~branching involved in salivary gland morphogenesis | 3 | 0.010607634 | BMP7, FGFR2, FGFR1 | 0.107888473 |
| GOTERM_BP_DIRECT | GO:0003181~atrioventricular valve morphogenesis | 3 | 0.010607634 | TGFB2, MDM2, TGFBR2 | 0.107888473 |
| GOTERM_BP_DIRECT | GO:0017187~peptidyl-glutamic acid carboxylation | 3 | 0.010607634 | F7, F10, F2 | 0.107888473 |
| GOTERM_BP_DIRECT | GO:0002523~leukocyte migration involved in inflammatory response | 3 | 0.010607634 | SELE, S100A9, ELANE | 0.107888473 |
| GOTERM_BP_DIRECT | GO:0001974~blood vessel remodeling | 4 | 0.011017167 | TGFB2, ACE, NOS3, MDM2 | 0.110670394 |
| GOTERM_BP_DIRECT | GO:0045907~positive regulation of vasoconstriction | 4 | 0.011017167 | FGG, ABL1, AKT1, EGFR | 0.110670394 |
| GOTERM_BP_DIRECT | GO:0046677~response to antibiotic | 4 | 0.011017167 | HSP90AA1, CASP3, MDM2, JAK2 | 0.110670394 |
| GOTERM_BP_DIRECT | GO:0051289~protein homotetramerization | 5 | 0.011231862 | RXRA, SHMT1, HPRT1, SOD2, CRYZ | 0.111906025 |
| GOTERM_BP_DIRECT | GO:0050679~positive regulation of epithelial cell proliferation | 5 | 0.011231862 | CCL5, IGF1, HRAS, EGFR, FGFR2 | 0.111906025 |
| GOTERM_BP_DIRECT | GO:0008152~metabolic process | 8 | 0.011587624 | ARSA, GSTM1, GSTO1, EPHX2, GSTP1, LYZ, ACAT1, CES1 | 0.114981263 |
| GOTERM_BP_DIRECT | GO:0006950~response to stress | 5 | 0.011886208 | HSP90AA1, MAPK8, AKR1B1, MAPK1, EGFR | 0.117080903 |
| GOTERM_BP_DIRECT | GO:0043491~protein kinase B signaling | 4 | 0.01199108 | CCL5, AKT1, IGF1, EPHA2 | 0.117080903 |
| GOTERM_BP_DIRECT | GO:0010634~positive regulation of epithelial cell migration | 4 | 0.01199108 | TGFB2, SRC, RAB11A, TGFBR2 | 0.117080903 |
| GOTERM_BP_DIRECT | GO:0071364~cellular response to epidermal growth factor stimulus | 4 | 0.01199108 | PDPK1, GSTP1, AKT1, EGFR | 0.117080903 |
| GOTERM_BP_DIRECT | GO:0071872~cellular response to epinephrine stimulus | 3 | 0.012608145 | PDE4D, PDE4B, PRKACA | 0.120220628 |
| GOTERM_BP_DIRECT | GO:0097067~cellular response to thyroid hormone stimulus | 3 | 0.012608145 | KIT, CTSS, CTSB | 0.120220628 |
| GOTERM_BP_DIRECT | GO:0000050~urea cycle | 3 | 0.012608145 | ARG2, ARG1, OTC | 0.120220628 |
| GOTERM_BP_DIRECT | GO:0006069~ethanol oxidation | 3 | 0.012608145 | ALDH2, ADH1C, ADH5 | 0.120220628 |
| GOTERM_BP_DIRECT | GO:0019430~removal of superoxide radicals | 3 | 0.012608145 | NQO1, NOS3, SOD2 | 0.120220628 |
| GOTERM_BP_DIRECT | GO:0045667~regulation of osteoblast differentiation | 3 | 0.012608145 | PRKACA, PTK2, FGFR2 | 0.120220628 |
| GOTERM_BP_DIRECT | GO:0043524~negative regulation of neuron apoptotic process | 7 | 0.012705177 | GPI, PDPK1, HMOX1, JAK2, SOD2, HRAS, BCL2L1 | 0.120674462 |
| GOTERM_BP_DIRECT | GO:0006096~glycolytic process | 4 | 0.013012916 | GPI, TPI1, GCK, HK1 | 0.12311833 |
| GOTERM_BP_DIRECT | GO:0009611~response to wounding | 5 | 0.013267112 | TGFB2, NMNAT1, FABP5, RAC1, F2 | 0.125038689 |
| GOTERM_BP_DIRECT | GO:0005975~carbohydrate metabolic process | 8 | 0.013808677 | CHIT1, GPI, ALDH2, GLO1, INSR, GBA, AKR1B1, ABO | 0.12964223 |
| GOTERM_BP_DIRECT | GO:0034599~cellular response to oxidative stress | 5 | 0.013994242 | G6PD, PARP1, ABL1, HSPA1B, HSPA1A | 0.12972307 |
| GOTERM_BP_DIRECT | GO:0030512~negative regulation of transforming growth factor beta receptor signaling pathway | 5 | 0.013994242 | ADAM17, PDPK1, TGFBR1, HSPA1A, TGFBR2 | 0.12972307 |
| GOTERM_BP_DIRECT | GO:0001890~placenta development | 4 | 0.014083004 | ANG, PPARG, MAPK14, PTK2 | 0.12972307 |
| GOTERM_BP_DIRECT | GO:0071902~positive regulation of protein serine/threonine kinase activity | 4 | 0.014083004 | MAP2K1, SRC, RHOA, CDK5R1 | 0.12972307 |
| GOTERM_BP_DIRECT | GO:0050918~positive chemotaxis | 4 | 0.014083004 | LGALS3, CCL5, MIF, MET | 0.12972307 |
| GOTERM_BP_DIRECT | GO:0006469~negative regulation of protein kinase activity | 6 | 0.014733807 | RTN4R, FABP4, PDPK1, GSTP1, AKT1, GP1BA | 0.133432708 |
| GOTERM_BP_DIRECT | GO:0045780~positive regulation of bone resorption | 3 | 0.014759046 | SYK, CA2, EGFR | 0.133432708 |
| GOTERM_BP_DIRECT | GO:0033630~positive regulation of cell adhesion mediated by integrin | 3 | 0.014759046 | ZAP70, TGFB2, SYK | 0.133432708 |
| GOTERM_BP_DIRECT | GO:0060389~pathway-restricted SMAD protein phosphorylation | 3 | 0.014759046 | TGFB2, TGFBR1, TGFBR2 | 0.133432708 |
| GOTERM_BP_DIRECT | GO:0060644~mammary gland epithelial cell differentiation | 3 | 0.014759046 | ERBB4, AKT2, AKT1 | 0.133432708 |
| GOTERM_BP_DIRECT | GO:0010043~response to zinc ion | 4 | 0.015201623 | CA2, ARG1, SOD2, OTC | 0.13642339 |
| GOTERM_BP_DIRECT | GO:0048469~cell maturation | 4 | 0.015201623 | BTK, REN, PPARG, FGFR1 | 0.13642339 |
| GOTERM_BP_DIRECT | GO:0006897~endocytosis | 7 | 0.016040412 | CDC42, EEA1, CD209, HRAS, RAB5A, PIK3CG, BCL2L1 | 0.143423607 |
| GOTERM_BP_DIRECT | GO:0032868~response to insulin | 5 | 0.016325546 | FABP3, PCK1, PPARA, HADH, OTC | 0.145440359 |
| GOTERM_BP_DIRECT | GO:0031103~axon regeneration | 3 | 0.017055582 | DHFR, CTNNA1, JAK2 | 0.147956017 |
| GOTERM_BP_DIRECT | GO:0043254~regulation of protein complex assembly | 3 | 0.017055582 | HSPA8, HSP90AA1, PTPN11 | 0.147956017 |
| GOTERM_BP_DIRECT | GO:0019395~fatty acid oxidation | 3 | 0.017055582 | GCDH, PPARG, MAPK14 | 0.147956017 |
| GOTERM_BP_DIRECT | GO:0001678~cellular glucose homeostasis | 3 | 0.017055582 | PIK3R1, GCK, HK1 | 0.147956017 |
| GOTERM_BP_DIRECT | GO:0030194~positive regulation of blood coagulation | 3 | 0.017055582 | F7, F2, S100A9 | 0.147956017 |
| GOTERM_BP_DIRECT | GO:0060347~heart trabecula formation | 3 | 0.017055582 | FKBP1A, RBP4, TEK | 0.147956017 |
| GOTERM_BP_DIRECT | GO:0045579~positive regulation of B cell differentiation | 3 | 0.017055582 | ZAP70, SYK, BTK | 0.147956017 |
| GOTERM_BP_DIRECT | GO:0007050~cell cycle arrest | 7 | 0.017094755 | TGFB2, MAP2K1, CDK6, RHEB, ABL1, HRAS, TGFBR1 | 0.147956017 |
| GOTERM_BP_DIRECT | GO:0009749~response to glucose | 5 | 0.017153442 | PKLR, CASP3, APOA2, TGFBR2, PPARD | 0.147956017 |
| GOTERM_BP_DIRECT | GO:0042157~lipoprotein metabolic process | 4 | 0.017585349 | ALB, APOA2, PPARA, PRKACA | 0.150616971 |
| GOTERM_BP_DIRECT | GO:0034446~substrate adhesion-dependent cell spreading | 4 | 0.017585349 | SRC, ABL1, TEK, RAC1 | 0.150616971 |
| GOTERM_BP_DIRECT | GO:0035690~cellular response to drug | 5 | 0.01800712 | RAP2A, NOS2, PDE4B, REN, EGFR | 0.153690139 |
| GOTERM_BP_DIRECT | GO:0051091~positive regulation of sequence-specific DNA binding transcription factor activity | 6 | 0.018560509 | KIT, AKT1, PPARG, JAK2, ESR1, ESR2 | 0.15786133 |
| GOTERM_BP_DIRECT | GO:0055088~lipid homeostasis | 4 | 0.018850796 | GCDH, NR1H3, PPARG, ACADM | 0.159524777 |
| GOTERM_BP_DIRECT | GO:0033138~positive regulation of peptidyl-serine phosphorylation | 5 | 0.018886793 | CDC42, GSK3B, AKT1, MIF, RAF1 | 0.159524777 |
| GOTERM_BP_DIRECT | GO:2000251~positive regulation of actin cytoskeleton reorganization | 3 | 0.019493102 | HCK, TEK, HRAS | 0.162398167 |
| GOTERM_BP_DIRECT | GO:0032270~positive regulation of cellular protein metabolic process | 3 | 0.019493102 | NR1H2, NR1H3, AKT1 | 0.162398167 |
| GOTERM_BP_DIRECT | GO:0022408~negative regulation of cell-cell adhesion | 3 | 0.019493102 | ABL1, JAK2, PTK2 | 0.162398167 |
| GOTERM_BP_DIRECT | GO:0006198~cAMP catabolic process | 3 | 0.019493102 | PDE4D, PDE3B, PDE4B | 0.162398167 |
| GOTERM_BP_DIRECT | GO:0097190~apoptotic signaling pathway | 5 | 0.019792663 | APAF1, DAPK1, CASP3, BTK, PPARD | 0.164332958 |
| GOTERM_BP_DIRECT | GO:0051262~protein tetramerization | 4 | 0.020165458 | CCL5, SHMT1, HMGCR, IGF1R | 0.1662969 |
| GOTERM_BP_DIRECT | GO:0030218~erythrocyte differentiation | 4 | 0.020165458 | THRA, CASP3, KIT, JAK2 | 0.1662969 |
| GOTERM_BP_DIRECT | GO:0044255~cellular lipid metabolic process | 4 | 0.021529404 | RXRA, NR1H2, NR1H3, PPARA | 0.176353268 |
| GOTERM_BP_DIRECT | GO:0032024~positive regulation of insulin secretion | 4 | 0.021529404 | RBP4, JAK2, GCK, PPARD | 0.176353268 |
| GOTERM_BP_DIRECT | GO:0032259~methylation | 5 | 0.021683751 | PNMT, MTAP, AHCY, GSTO1, HNMT | 0.177023532 |
| GOTERM_BP_DIRECT | GO:0035162~embryonic hemopoiesis | 3 | 0.02206706 | KIT, KDR, TGFBR2 | 0.17718978 |
| GOTERM_BP_DIRECT | GO:0035994~response to muscle stretch | 3 | 0.02206706 | GPI, RAF1, MAPK14 | 0.17718978 |
| GOTERM_BP_DIRECT | GO:0010592~positive regulation of lamellipodium assembly | 3 | 0.02206706 | HSP90AA1, RAC2, RAC1 | 0.17718978 |
| GOTERM_BP_DIRECT | GO:0010613~positive regulation of cardiac muscle hypertrophy | 3 | 0.02206706 | PARP1, PDE5A, IGF1 | 0.17718978 |
| GOTERM_BP_DIRECT | GO:0097194~execution phase of apoptosis | 3 | 0.02206706 | CASP7, CASP3, AKT1 | 0.17718978 |
| GOTERM_BP_DIRECT | GO:0071356~cellular response to tumor necrosis factor | 6 | 0.022209132 | FABP4, CCL5, GBA, LCN2, RORA, PCK1 | 0.177745873 |
| GOTERM_BP_DIRECT | GO:0001889~liver development | 5 | 0.022669319 | ARG1, ACADM, SOD2, OTC, ACAT1 | 0.180246927 |
| GOTERM_BP_DIRECT | GO:0030334~regulation of cell migration | 5 | 0.022669319 | ERBB4, AKT2, AKT1, RAC1, RHOA | 0.180246927 |
| GOTERM_BP_DIRECT | GO:0001541~ovarian follicle development | 4 | 0.022942666 | KIT, CTNNA1, ANG, BCL2L1 | 0.181828073 |
| GOTERM_BP_DIRECT | GO:0007623~circadian rhythm | 5 | 0.023681784 | GSK3B, F7, TPH1, NOS2, EGFR | 0.187078426 |
| GOTERM_BP_DIRECT | GO:0014823~response to activity | 4 | 0.02440524 | PCK1, SOD2, HADH, PPARD | 0.192171582 |
| GOTERM_BP_DIRECT | GO:0031333~negative regulation of protein complex assembly | 3 | 0.024773009 | CDC42, GSK3B, RAF1 | 0.19258253 |
| GOTERM_BP_DIRECT | GO:2000811~negative regulation of anoikis | 3 | 0.024773009 | SRC, PTK2, BCL2L1 | 0.19258253 |
| GOTERM_BP_DIRECT | GO:1901216~positive regulation of neuron death | 3 | 0.024773009 | GSK3B, PARP1, ABL1 | 0.19258253 |
| GOTERM_BP_DIRECT | GO:0090201~negative regulation of release of cytochrome c from mitochondria | 3 | 0.024773009 | AKT1, IGF1, BCL2L1 | 0.19258253 |
| GOTERM_BP_DIRECT | GO:0006094~gluconeogenesis | 4 | 0.025917088 | GPI, RBP4, TPI1, PCK1 | 0.199569752 |
| GOTERM_BP_DIRECT | GO:0006635~fatty acid beta-oxidation | 4 | 0.025917088 | ACADM, HADH, ACAT1, PPARD | 0.199569752 |
| GOTERM_BP_DIRECT | GO:0016241~regulation of macroautophagy | 4 | 0.025917088 | MAPK8, CASP3, GBA, CDK5R1 | 0.199569752 |
| GOTERM_BP_DIRECT | GO:0045766~positive regulation of angiogenesis | 6 | 0.02629441 | NOS3, CMA1, KDR, HMOX1, TEK, TGFBR2 | 0.201838538 |
| GOTERM_BP_DIRECT | GO:0006629~lipid metabolic process | 7 | 0.027284914 | G6PD, FABP5, TTPA, PITPNA, PPARG, PPARA, PPARD | 0.208785185 |
| GOTERM_BP_DIRECT | GO:0060324~face development | 3 | 0.027606603 | MAP2K1, MAPK1, RAF1 | 0.209278627 |
| GOTERM_BP_DIRECT | GO:0030225~macrophage differentiation | 3 | 0.027606603 | CDC42, PARP1, MMP9 | 0.209278627 |
| GOTERM_BP_DIRECT | GO:0033189~response to vitamin A | 3 | 0.027606603 | ARG1, PPARG, PPARD | 0.209278627 |
| GOTERM_BP_DIRECT | GO:0038183~bile acid signaling pathway | 2 | 0.028851169 | VDR, NR1H4 | 0.212391369 |
| GOTERM_BP_DIRECT | GO:1901898~negative regulation of relaxation of cardiac muscle | 2 | 0.028851169 | PDE4D, PDE4B | 0.212391369 |
| GOTERM_BP_DIRECT | GO:1904722~positive regulation of mRNA endonucleolytic cleavage involved in unfolded protein response | 2 | 0.028851169 | HSPA1B, HSPA1A | 0.212391369 |
| GOTERM_BP_DIRECT | GO:0006738~nicotinamide riboside catabolic process | 2 | 0.028851169 | MTAP, PNP | 0.212391369 |
| GOTERM_BP_DIRECT | GO:0014806~smooth muscle hyperplasia | 2 | 0.028851169 | NOS3, HMOX1 | 0.212391369 |
| GOTERM_BP_DIRECT | GO:0043366~beta selection | 2 | 0.028851169 | ZAP70, SYK | 0.212391369 |
| GOTERM_BP_DIRECT | GO:0035607~fibroblast growth factor receptor signaling pathway involved in orbitofrontal cortex development | 2 | 0.028851169 | FGFR2, FGFR1 | 0.212391369 |
| GOTERM_BP_DIRECT | GO:0044209~AMP salvage | 2 | 0.028851169 | ADK, APRT | 0.212391369 |
| GOTERM_BP_DIRECT | GO:1902380~positive regulation of endoribonuclease activity | 2 | 0.028851169 | HSPA1B, HSPA1A | 0.212391369 |
| GOTERM_BP_DIRECT | GO:0007411~axon guidance | 7 | 0.028887314 | TGFB2, MAPK1, RAC1, HRAS, BMP7, PTK2, CDK5R1 | 0.212391369 |
| GOTERM_BP_DIRECT | GO:0032729~positive regulation of interferon-gamma production | 4 | 0.029088285 | PDE4D, PDE4B, HRAS, IL2 | 0.212420578 |
| GOTERM_BP_DIRECT | GO:0000186~activation of MAPKK activity | 4 | 0.029088285 | JAK2, RAF1, EGFR, TGFBR1 | 0.212420578 |
| GOTERM_BP_DIRECT | GO:0046718~viral entry into host cell | 5 | 0.029152353 | DPP4, CD209, HSPA1B, CTSB, HSPA1A | 0.212420578 |
| GOTERM_BP_DIRECT | GO:0060314~regulation of ryanodine-sensitive calcium-release channel activity | 3 | 0.030563594 | FKBP1A, PDE4D, PRKACA | 0.218145419 |
| GOTERM_BP_DIRECT | GO:0046827~positive regulation of protein export from nucleus | 3 | 0.030563594 | GSK3B, MDM2, PRKACA | 0.218145419 |
| GOTERM_BP_DIRECT | GO:0009116~nucleoside metabolic process | 3 | 0.030563594 | MTAP, PNP, APRT | 0.218145419 |
| GOTERM_BP_DIRECT | GO:1902236~negative regulation of endoplasmic reticulum stress-induced intrinsic apoptotic signaling pathway | 3 | 0.030563594 | PTPN1, HSPA1B, HSPA1A | 0.218145419 |
| GOTERM_BP_DIRECT | GO:0051493~regulation of cytoskeleton organization | 3 | 0.030563594 | MAPK1, S100A9, PTK2 | 0.218145419 |
| GOTERM_BP_DIRECT | GO:0043393~regulation of protein binding | 3 | 0.030563594 | SRC, PRKACA, TGFBR1 | 0.218145419 |
| GOTERM_BP_DIRECT | GO:0050771~negative regulation of axonogenesis | 3 | 0.030563594 | RTN4R, PTK2, RHOA | 0.218145419 |
| GOTERM_BP_DIRECT | GO:0032956~regulation of actin cytoskeleton organization | 4 | 0.030747395 | TGFB2, ABL1, RHOA, CDK5R1 | 0.218181367 |
| GOTERM_BP_DIRECT | GO:0042110~T cell activation | 4 | 0.030747395 | FKBP1A, DPP4, ZAP70, PIK3CG | 0.218181367 |
| GOTERM_BP_DIRECT | GO:0008543~fibroblast growth factor receptor signaling pathway | 5 | 0.031533198 | NDST1, MAPK1, PTPN11, FGFR2, FGFR1 | 0.2231088 |
| GOTERM_BP_DIRECT | GO:0007267~cell-cell signaling | 9 | 0.032365542 | AR, TGFB2, CCL5, PGR, TEK, S100A9, ESR2, IL2, FGFR2 | 0.228336091 |
| GOTERM_BP_DIRECT | GO:0014911~positive regulation of smooth muscle cell migration | 3 | 0.033639829 | SRC, CCL5, IGF1 | 0.233280746 |
| GOTERM_BP_DIRECT | GO:0071375~cellular response to peptide hormone stimulus | 3 | 0.033639829 | SRC, MDM2, CSK | 0.233280746 |
| GOTERM_BP_DIRECT | GO:0048545~response to steroid hormone | 3 | 0.033639829 | CA2, MDM2, TGFBR2 | 0.233280746 |
| GOTERM_BP_DIRECT | GO:0035584~calcium-mediated signaling using intracellular calcium source | 3 | 0.033639829 | SELP, KDR, PRKACA | 0.233280746 |
| GOTERM_BP_DIRECT | GO:0046697~decidualization | 3 | 0.033639829 | VDR, CTSB, PPARD | 0.233280746 |
| GOTERM_BP_DIRECT | GO:0050850~positive regulation of calcium-mediated signaling | 3 | 0.033639829 | ZAP70, HINT1, SYK | 0.233280746 |
| GOTERM_BP_DIRECT | GO:0021762~substantia nigra development | 4 | 0.034211817 | CDC42, G6PD, MAOB, RHOA | 0.23590691 |
| GOTERM_BP_DIRECT | GO:0071560~cellular response to transforming growth factor beta stimulus | 4 | 0.034211817 | APAF1, ARG1, ABL1, TGFBR1 | 0.23590691 |
| GOTERM_BP_DIRECT | GO:1901796~regulation of signal transduction by p53 class mediator | 6 | 0.034793923 | CSNK2A1, CHEK1, MDM2, AKT1, MAPK14, CDK5R1 | 0.239244973 |
| GOTERM_BP_DIRECT | GO:0006928~movement of cell or subcellular component | 5 | 0.036628665 | MAP2K1, RAC1, IGF1, JAK2, MAPK14 | 0.249736326 |
| GOTERM_BP_DIRECT | GO:0030520~intracellular estrogen receptor signaling pathway | 3 | 0.036831248 | SRC, ESR1, ESR2 | 0.249736326 |
| GOTERM_BP_DIRECT | GO:0051894~positive regulation of focal adhesion assembly | 3 | 0.036831248 | KDR, TEK, RAC1 | 0.249736326 |
| GOTERM_BP_DIRECT | GO:0045921~positive regulation of exocytosis | 3 | 0.036831248 | NCS1, FGG, RAB5A | 0.249736326 |
| GOTERM_BP_DIRECT | GO:0006921~cellular component disassembly involved in execution phase of apoptosis | 3 | 0.036831248 | CASP7, CASP3, PTK2 | 0.249736326 |
| GOTERM_BP_DIRECT | GO:0043547~positive regulation of GTPase activity | 15 | 0.037060022 | RTN4R, GSK3B, EGFR, IL2, PTK2, ERBB4, CCL5, KIT, TEK, RAC1, JAK2, HRAS, JAK3, FGFR2, FGFR1 | 0.250591448 |
| GOTERM_BP_DIRECT | GO:0046427~positive regulation of JAK-STAT cascade | 3 | 0.040133885 | CCL5, KIT, AKR1B1 | 0.267668889 |
| GOTERM_BP_DIRECT | GO:2001243~negative regulation of intrinsic apoptotic signaling pathway | 3 | 0.040133885 | SRC, MMP9, BCL2L1 | 0.267668889 |
| GOTERM_BP_DIRECT | GO:0045453~bone resorption | 3 | 0.040133885 | SRC, RAC2, RAC1 | 0.267668889 |
| GOTERM_BP_DIRECT | GO:0060999~positive regulation of dendritic spine development | 3 | 0.040133885 | ARF1, RAC1, IL2 | 0.267668889 |
| GOTERM_BP_DIRECT | GO:0001502~cartilage condensation | 3 | 0.040133885 | TGFB2, THRA, MAPK14 | 0.267668889 |
| GOTERM_BP_DIRECT | GO:0019221~cytokine-mediated signaling pathway | 6 | 0.042457157 | RTN4R, FKBP1A, HCK, KIT, GP1BA, JAK2 | 0.271699425 |
| GOTERM_BP_DIRECT | GO:0072136~metanephric mesenchymal cell proliferation involved in metanephros development | 2 | 0.04296435 | STAT1, BMP7 | 0.271699425 |
| GOTERM_BP_DIRECT | GO:0019254~carnitine metabolic process, CoA-linked | 2 | 0.04296435 | ACADM, CRAT | 0.271699425 |
| GOTERM_BP_DIRECT | GO:0070434~positive regulation of nucleotide-binding oligomerization domain containing 2 signaling pathway | 2 | 0.04296435 | HSPA1B, HSPA1A | 0.271699425 |
| GOTERM_BP_DIRECT | GO:0070141~response to UV-A | 2 | 0.04296435 | AKT1, EGFR | 0.271699425 |
| GOTERM_BP_DIRECT | GO:0071226~cellular response to molecule of fungal origin | 2 | 0.04296435 | SYK, BTK | 0.271699425 |
| GOTERM_BP_DIRECT | GO:0061684~chaperone-mediated autophagy | 2 | 0.04296435 | HSPA8, HSP90AA1 | 0.271699425 |
| GOTERM_BP_DIRECT | GO:0021847~ventricular zone neuroblast division | 2 | 0.04296435 | FGFR2, FGFR1 | 0.271699425 |
| GOTERM_BP_DIRECT | GO:0021697~cerebellar cortex formation | 2 | 0.04296435 | MAP2K1, PTPN11 | 0.271699425 |
| GOTERM_BP_DIRECT | GO:0010641~positive regulation of platelet-derived growth factor receptor signaling pathway | 2 | 0.04296435 | F7, SRC | 0.271699425 |
| GOTERM_BP_DIRECT | GO:0010871~negative regulation of receptor biosynthetic process | 2 | 0.04296435 | PPARG, PPARA | 0.271699425 |
| GOTERM_BP_DIRECT | GO:0031281~positive regulation of cyclase activity | 2 | 0.04296435 | MAPK8, MAPK14 | 0.271699425 |
| GOTERM_BP_DIRECT | GO:0060523~prostate epithelial cord elongation | 2 | 0.04296435 | ESR1, FGFR2 | 0.271699425 |
| GOTERM_BP_DIRECT | GO:0008295~spermidine biosynthetic process | 2 | 0.04296435 | AMD1, SRM | 0.271699425 |
| GOTERM_BP_DIRECT | GO:0060745~mammary gland branching involved in pregnancy | 2 | 0.04296435 | VDR, ESR1 | 0.271699425 |
| GOTERM_BP_DIRECT | GO:0006168~adenine salvage | 2 | 0.04296435 | HPRT1, APRT | 0.271699425 |
| GOTERM_BP_DIRECT | GO:0003274~endocardial cushion fusion | 2 | 0.04296435 | TGFB2, TGFBR2 | 0.271699425 |
| GOTERM_BP_DIRECT | GO:0090135~actin filament branching | 2 | 0.04296435 | CDC42, ABL1 | 0.271699425 |
| GOTERM_BP_DIRECT | GO:0045726~positive regulation of integrin biosynthetic process | 2 | 0.04296435 | AR, TGFB2 | 0.271699425 |
| GOTERM_BP_DIRECT | GO:0051791~medium-chain fatty acid metabolic process | 2 | 0.04296435 | ACADM, CES1 | 0.271699425 |
| GOTERM_BP_DIRECT | GO:0016239~positive regulation of macroautophagy | 3 | 0.043543862 | GBA, KDR, HMOX1 | 0.271842882 |
| GOTERM_BP_DIRECT | GO:0046835~carbohydrate phosphorylation | 3 | 0.043543862 | ADK, GCK, HK1 | 0.271842882 |
| GOTERM_BP_DIRECT | GO:0030316~osteoclast differentiation | 3 | 0.043543862 | GLO1, MAPK14, EPHA2 | 0.271842882 |
| GOTERM_BP_DIRECT | GO:0035924~cellular response to vascular endothelial growth factor stimulus | 3 | 0.043543862 | KDR, AKT1, MAPK14 | 0.271842882 |
| GOTERM_BP_DIRECT | GO:0009880~embryonic pattern specification | 3 | 0.043543862 | ERBB4, BMP7, FGFR2 | 0.271842882 |
| GOTERM_BP_DIRECT | GO:0060548~negative regulation of cell death | 4 | 0.045757557 | TTPA, BMP7, HSPA1B, HSPA1A | 0.284934174 |
| GOTERM_BP_DIRECT | GO:0070371~ERK1 and ERK2 cascade | 3 | 0.04705739 | MAP2K1, MAPK1, IGF1 | 0.29228267 |
| GOTERM_BP_DIRECT | GO:0001570~vasculogenesis | 4 | 0.047846777 | KDR, PTK2, EPHA2, TGFBR2 | 0.296431428 |
| GOTERM_BP_DIRECT | GO:0032091~negative regulation of protein binding | 4 | 0.049982177 | GSK3B, MAPK8, ACE, PPARA | 0.306916486 |
