# Supplementary material for: Total Flavonoids of Chuju Decrease Oxidative Stress and Cell Apoptosis in Ischemic Stroke Rats: Network and Experimental Analyses
Source: Front Neurosci. 2021 Dec 9;15:772401. doi: 10.3389/fnins.2021.772401 (PMC8695723; doi:10.3389/fnins.2021.772401)
Supplement: Supplementary file 5 [file Table_4.docx]

Supplementary Table 4 Functions of potential target genes based on GO cellular component

| Category | Term | Count | PValue | Genes | FDR |
| --- | --- | --- | --- | --- | --- |
| GOTERM_CC_DIRECT | GO:0005829~cytosol | 129 | 7.06E-34 | PNMT, HNMT, PNP, AKT2, CHEK1, AKT1, PRKACA, EPHB4, MAP2K1, G6PD, CSNK2A1, AR, RBP4, MTAP, BTK, PADI4, HPRT1, RAF1, PPIA, S100A9, CES1, TPH1, SHMT1, GLO1, PIK3R1, ADH5, TTPA, ABL1, PNPO, HMOX1, LTA4H, PCK1, HSPA8, GSTM1, EPHX2, PTK2, DHFR, BHMT, CDK6, RHEB, PAH, LCN2, MDM2, RAB5A, BCL2L1, FGFR1, GPI, GSK3B, ARF1, THRA, AMD1, PDE3B, ADK, AKR1B1, PIK3CG, HK1, EEA1, CASP7, CA1, IMPA1, CA2, CASP3, NCS1, PDE4B, CASP1, RAC2, RAC1, JAK2, JAK3, HRAS, HSP90AA1, TPI1, APAF1, SYK, PDPK1, ARG1, GSTO1, PDE4D, APOA2, DUSP6, RHOA, APRT, TGFBR2, ZAP70, HCK, RAP2A, LCK, BLVRB, PPARG, PDE5A, AHCY, ADH1C, SRC, GSTP1, XIAP, SRM, TYMP, CRYZ, CDC42, MAPK8, SULT1E1, ERBB4, CBS, CTNNA1, MAPK1, CSK, GC, PTPN1, NQO1, PKLR, NOS2, STAT1, NOS3, GSR, PTPN11, MAPK14, RAB11A, GCK, GSTZ1, MAPK10, FKBP1A, FABP3, FABP4, FABP5, FABP7, RAN, HSPA1B, CDK5R1, HSPA1A | 1.74E-31 |
| GOTERM_CC_DIRECT | GO:0070062~extracellular exosome | 112 | 4.39E-29 | REG1A, PITPNA, ICAM2, HNMT, LGALS3, PNP, PLAU, PRKACA, EPHB4, ARSA, MAP2K1, G6PD, RNASE3, MIF, RBP4, MTAP, FOLH1, HPRT1, PPIA, S100A9, CFB, RTN4R, CFD, MAOB, SHMT1, GLO1, NPR3, ADH5, ACAT1, DPP4, SEC14L2, PNPO, LTA4H, PCK1, ELANE, HSPA8, ACE, EPHX2, INSR, LYZ, BHMT, CD209, RHEB, PAH, ALB, LCN2, SHBG, RAB5A, GPI, ARF1, SERPINA1, AKR1B1, RND3, EEA1, HINT1, CA1, IMPA1, GM2A, ALDH2, CA2, NCS1, RAC2, CTSG, ACADM, RAC1, CTSD, CTSB, HSP90AA1, MMP7, TPI1, APAF1, MME, ARG1, GSTO1, ANXA5, FGG, APOA2, GP1BA, F2, MMP9, RHOA, APRT, RAP2A, LCK, BLVRB, ANG, APCS, AHCY, C1S, SRC, C1R, GSTP1, GBA, CRYZ, CDC42, TTR, MAPK1, CSK, GC, NQO1, PKLR, F11, GSR, PLA2G2A, MAPK14, SOD2, RAB11A, FKBP1A, FABP3, FABP4, FABP5, RAN | 5.40E-27 |
| GOTERM_CC_DIRECT | GO:0005615~extracellular space | 59 | 9.90E-16 | GPI, SERPINA1, AKR1B1, CTSS, LGALS3, PLAU, CA2, CHEK1, CTSG, CTSD, CTSB, ARSA, MMP7, TPI1, ARG1, MMP2, FGG, MMP3, RNASE3, MIF, MMP8, F2, MMP9, CHIT1, F7, RBP4, MMP13, KIT, ANG, S100A9, PPIA, CFB, CES1, CFD, APCS, GSTP1, GBA, EGFR, TTR, CCL5, HMOX1, GC, ELANE, HSPA8, TGFB2, ACE, PLA2G2A, F11, IGF1, LYZ, SELE, BMP7, IL2, SELP, FABP3, FAP, ALB, LCN2, REN | 8.12E-14 |
| GOTERM_CC_DIRECT | GO:0005576~extracellular region | 61 | 1.87E-13 | SERPINA1, CTSS, PNP, PLAU, CASP1, KDR, CTSG, CTSD, EPHB4, CTSB, HSP90AA1, MMP7, MMP1, MMP2, FGG, MMP3, APOA2, RNASE3, MIF, MMP8, F2, MMP9, CHIT1, MMP12, F7, RBP4, MMP13, ANG, S100A9, MET, PPIA, CFB, CFD, APCS, C1S, C1R, ABO, TTR, ERBB4, CCL5, GC, ELANE, BCHE, TGFB2, ACE, F10, CMA1, PLA2G2A, F11, IGF1, LYZ, BMP7, ESR2, IL2, ALB, LCN2, REN, TEK, SHBG, FGFR2, FGFR1 | 1.15E-11 |
| GOTERM_CC_DIRECT | GO:0005925~focal adhesion | 25 | 6.48E-10 | ARF1, RND3, EGFR, DPP4, CDC42, PLAU, CTNNA1, RAC2, MAPK1, RAC1, HSPA8, MAP2K1, MME, PDPK1, ANXA5, RHOA, PTK2, HCK, ADAM17, FAP, TEK, PPIA, HSPA1B, HSPA1A, EPHA2 | 3.19E-08 |
| GOTERM_CC_DIRECT | GO:0031234~extrinsic component of cytoplasmic side of plasma membrane | 12 | 1.62E-09 | ZAP70, HCK, SYK, SRC, LCK, BTK, ABL1, CSK, JAK2, JAK3, PTK2, RHOA | 6.63E-08 |
| GOTERM_CC_DIRECT | GO:0045121~membrane raft | 18 | 2.84E-09 | SELE, EGFR, TGFBR1, TGFBR2, HK1, BACE1, DPP4, ZAP70, ADAM17, LCK, CASP3, KDR, BTK, CSK, TEK, JAK2, CTSD, RAB5A | 9.99E-08 |
| GOTERM_CC_DIRECT | GO:0031012~extracellular matrix | 19 | 1.12E-07 | APCS, HSPA8, TGFB2, HSP90AA1, MMP7, MMP1, CMA1, MMP2, MMP8, BMP7, FKBP1A, LGALS3, MMP13, CTSG, RAC1, CTSD, S100A9, RAN, FGFR2 | 3.45E-06 |
| GOTERM_CC_DIRECT | GO:0072562~blood microparticle | 13 | 1.03E-06 | APCS, BCHE, HSPA8, C1S, C1R, FGG, APOA2, F2, ALB, GC, HSPA1B, CFB, HSPA1A | 2.81E-05 |
| GOTERM_CC_DIRECT | GO:0005886~plasma membrane | 89 | 1.37E-06 | GPI, GSK3B, ARF1, ICAM2, PIK3CG, IGF1R, LGALS3, HINT1, STS, PLAU, CA2, CASP3, AKT2, NCS1, KDR, RAC2, AKT1, CTSG, RAC1, PRKACA, HRAS, EPHB4, MAP2K1, HSP90AA1, CSNK2A1, SYK, MME, PDPK1, DAPK1, MMP2, FGG, GP1BA, F2, RHOA, TGFBR1, TGFBR2, BACE1, F7, AR, ZAP70, RAP2A, ADAM17, FOLH1, LCK, KIT, BTK, BLVRB, RAF1, S100A9, MET, CFB, EPHA2, RTN4R, SRC, GSTP1, NPR3, PIK3R1, EGFR, DPP4, CDC42, ERBB4, CTNNA1, HMOX1, CSK, LTA4H, PTPN1, HSPA8, ACE, F10, NOS3, INSR, PLA2G2A, F11, IGF1, SELE, ESR1, RAB11A, PTK2, SELP, MAPK10, FAP, CD209, MDM2, REN, TEK, RAB5A, FGFR2, CDK5R1, FGFR1 | 3.37E-05 |
| GOTERM_CC_DIRECT | GO:0005901~caveola | 9 | 2.48E-06 | HCK, SRC, NOS3, INSR, HMOX1, MAPK1, JAK2, SELE, TGFBR2 | 5.55E-05 |
| GOTERM_CC_DIRECT | GO:0005739~mitochondrion | 40 | 3.27E-06 | GCDH, GSK3B, MAOB, FECH, SRC, SHMT1, GSTP1, ADH5, HK1, ACAT1, MAPK8, GM2A, ERBB4, CASP1, ABL1, AKT1, MAPK1, ACADM, HADH, PRKACA, CTSB, ARG2, MAP2K1, PARP1, MMP2, GSR, TAP1, PTPN11, MAPK14, SOD2, GCK, ESR2, RAB11A, GSTZ1, MAPK10, CRAT, HSPA1B, OTC, BCL2L1, HSPA1A | 6.50E-05 |
| GOTERM_CC_DIRECT | GO:0005654~nucleoplasm | 66 | 3.43E-06 | GPI, THRA, ADK, AKR1B1, RORA, NR3C1, HNMT, NR3C2, CASP7, CASP3, AKT2, CHEK1, AKT1, JAK2, PRKACA, HADH, HSP90AA1, PARP1, CSNK2A1, PDPK1, MIF, DUSP6, APRT, AR, BLVRB, RARB, PPARG, PGR, PADI4, PPARA, PPARD, NR1I2, XIAP, RXRB, MAPK8, RXRA, ERBB4, ABL1, PNPO, PMS2, MAPK1, LTA4H, HSPA8, STAT1, VDR, NR1H2, NR1H4, NR1H3, ESRRG, MAPK14, ESR1, ESR2, GCK, SELP, DHFR, MAPK10, CDK6, NMNAT1, FABP5, FABP7, MDM2, RAN, FGFR2, HSPA1B, CDK5R1, HSPA1A | 6.50E-05 |
| GOTERM_CC_DIRECT | GO:0005737~cytoplasm | 104 | 4.48E-06 | PITPNA, NR3C1, HNMT, LGALS3, PNP, AKT1, HADH, MAP2K1, G6PD, DAPK1, MIF, AR, MTAP, ADAM17, FOLH1, BTK, PADI4, HPRT1, RAF1, RTN4R, SHMT1, GLO1, PIK3R1, SEC14L2, ABL1, PNPO, LTA4H, PCK1, ELANE, GSTM1, EPHX2, ESR1, PTK2, SELP, BHMT, CDK6, CD209, MDM2, RAB5A, FGFR2, BCL2L1, GPI, GSK3B, ADK, AKR1B1, PIK3CG, EEA1, HINT1, CASP7, CA1, IMPA1, CA2, CASP3, NCS1, RAC2, RAC1, JAK2, HRAS, HSP90AA1, SYK, MME, PDPK1, ARG1, GSTO1, ANXA5, GP1BA, DUSP6, APRT, ZAP70, RARB, BLVRB, AHCY, SRC, GSTP1, XIAP, EGFR, CRYZ, CDC42, TTR, CBS, CCL5, PMS2, MAPK1, CSK, NQO1, NOS2, STAT1, CMA1, NOS3, NR1H2, PTPN11, MAPK14, GSTZ1, MAPK10, FKBP1A, FABP4, FABP5, FAP, FABP7, TEK, RAN, HSPA1B, CDK5R1, HSPA1A | 7.87E-05 |
| GOTERM_CC_DIRECT | GO:0043235~receptor complex | 11 | 8.48E-06 | RXRA, ERBB4, VDR, INSR, NR1H3, EGFR, TGFBR1, NR3C2, FGFR1, TGFBR2, IGF1R | 1.39E-04 |
| GOTERM_CC_DIRECT | GO:0009986~cell surface | 21 | 4.68E-05 | RTN4R, HSP90AA1, MMP7, FGG, GP1BA, MIF, EGFR, TGFBR1, BACE1, DPP4, ADAM17, FOLH1, FAP, PLAU, CD209, CTSG, TEK, MET, ELANE, FGFR2, EPHA2 | 7.20E-04 |
| GOTERM_CC_DIRECT | GO:0016020~membrane | 52 | 6.37E-05 | GPI, PDE3B, ICAM2, PIK3CG, IGF1R, EEA1, LGALS3, STS, PDE4B, RAC2, RAC1, JAK2, PRKACA, JAK3, HRAS, G6PD, HSP90AA1, PARP1, PDPK1, PDE4D, ANXA5, TAP1, GP1BA, TGFBR1, RAP2A, ADAM17, FOLH1, KIT, PPIA, PIK3R1, CYP19A1, ABO, EGFR, DPP4, HSD11B1, CDC42, HMOX1, BCHE, HSPA8, ACE, INSR, F11, ESR1, RAB11A, SELP, FKBP1A, CD209, RHEB, RAN, FGFR2, CDK5R1, BCL2L1 | 9.21E-04 |
| GOTERM_CC_DIRECT | GO:0043202~lysosomal lumen | 8 | 1.47E-04 | HSPA8, ARSA, HSP90AA1, GM2A, GBA, GC, CTSD, CTSS | 0.00201531 |
| GOTERM_CC_DIRECT | GO:0048471~perinuclear region of cytoplasm | 21 | 2.92E-04 | GSK3B, ARF1, HSP90AA1, NOS2, STAT1, SRC, PLA2G2A, AKR1B1, SELE, EGFR, RAB11A, NCS1, BTK, ABL1, HMOX1, PPARG, HRAS, HSPA1B, CTSB, HSPA1A, CDK5R1 | 0.003782292 |
| GOTERM_CC_DIRECT | GO:0042470~melanosome | 8 | 4.29E-04 | HSPA8, HSP90AA1, AHCY, RAC1, CTSD, RAB5A, RAN, CTSB | 0.005271103 |
| GOTERM_CC_DIRECT | GO:0005759~mitochondrial matrix | 14 | 5.08E-04 | GCDH, ARG2, FECH, GSR, SOD2, NR3C1, ACAT1, ALDH2, ERBB4, ACADM, HADH, AGXT, OTC, BCL2L1 | 0.005952455 |
| GOTERM_CC_DIRECT | GO:0005911~cell-cell junction | 10 | 5.44E-04 | CDC42, ZAP70, ADAM17, FABP7, KIT, CTNNA1, AKT1, CSK, TEK, PIK3R1 | 0.006087234 |
| GOTERM_CC_DIRECT | GO:0032587~ruffle membrane | 7 | 8.29E-04 | ADAM17, HSP90AA1, FAP, SRC, AKT2, RAC1, EPHA2 | 0.008590841 |
| GOTERM_CC_DIRECT | GO:0031093~platelet alpha granule lumen | 6 | 8.48E-04 | CFD, TGFB2, SERPINA1, ALB, FGG, IGF1 | 0.008590841 |
| GOTERM_CC_DIRECT | GO:0030424~axon | 11 | 8.73E-04 | BACE1, TGFB2, MME, STAT1, CA2, NCS1, MAPK1, ACADM, RAB5A, RAB11A, CDK5R1 | 0.008590841 |
| GOTERM_CC_DIRECT | GO:0043209~myelin sheath | 9 | 0.001059262 | CDC42, HSPA8, GPI, HSP90AA1, CA2, ALB, PITPNA, MIF, SOD2 | 0.010022253 |
| GOTERM_CC_DIRECT | GO:0005788~endoplasmic reticulum lumen | 10 | 0.001189686 | BACE1, ARSA, F7, BCHE, SERPINA1, STS, F10, APOA2, F2, CES1 | 0.010839357 |
| GOTERM_CC_DIRECT | GO:0042629~mast cell granule | 4 | 0.002668827 | KIT, BTK, AKR1B1, PIK3CG | 0.023447549 |
| GOTERM_CC_DIRECT | GO:0005768~endosome | 10 | 0.00348871 | BACE1, ARSA, TGFB2, ACE, STS, KDR, RAB5A, RHOA, EGFR, TGFBR1 | 0.028678967 |
| GOTERM_CC_DIRECT | GO:0005764~lysosome | 10 | 0.003592217 | CHIT1, ARSA, HCK, ACE, STS, SRC, REN, CTSD, CTSS, CTSB | 0.028678967 |
| GOTERM_CC_DIRECT | GO:0043025~neuronal cell body | 12 | 0.003716765 | RTN4R, CDC42, NQO1, GSK3B, TGFB2, HSP90AA1, ARG1, FABP7, ABL1, ANG, RAB5A, CDK5R1 | 0.028678967 |
| GOTERM_CC_DIRECT | GO:0043231~intracellular membrane-bounded organelle | 17 | 0.003730597 | G6PD, CMA1, INSR, CTSS, IGF1R, CYP2C9, CASP7, STS, NCS1, CHEK1, BTK, CTNNA1, PPARG, HRAS, FGFR2, CTSB, CDK5R1 | 0.028678967 |
| GOTERM_CC_DIRECT | GO:0043005~neuron projection | 10 | 0.004889536 | RTN4R, CDC42, GPI, ARF1, HSP90AA1, TPH1, AHCY, SRC, ARG1, HNMT | 0.036449266 |
| GOTERM_CC_DIRECT | GO:0030027~lamellipodium | 8 | 0.005976262 | DPP4, FAP, CTNNA1, RAC2, RAC1, PTK2, RHOA, EPHA2 | 0.043195818 |
| GOTERM_CC_DIRECT | GO:0005770~late endosome | 7 | 0.006145746 | BACE1, HSPA8, MAP2K1, ARF1, SRC, TTPA, MAPK1 | 0.043195818 |
| GOTERM_CC_DIRECT | GO:0005622~intracellular | 30 | 0.006958962 | GSTP1, NPR3, PITPNA, RND3, PIK3CG, CDC42, SEC14L2, MAPK8, PNP, RAC2, MAPK1, RAC1, HRAS, CTSB, HSPA8, NOS2, PDPK1, ANXA5, MAPK14, RHOA, TGFBR1, IL2, RAB11A, MAPK10, RAP2A, RHEB, RAF1, RAB5A, RAN, EPHA2 | 0.047552905 |
| GOTERM_CC_DIRECT | GO:0031982~vesicle | 7 | 0.007719255 | F7, GSTP1, AKT1, MIF, RHOA, ABO, RAB11A | 0.051322616 |
| GOTERM_CC_DIRECT | GO:0090575~RNA polymerase II transcription factor complex | 4 | 0.009726718 | RXRA, VDR, NR1H3, PPARG | 0.0629677 |
| GOTERM_CC_DIRECT | GO:0043234~protein complex | 13 | 0.010118129 | HSP90AA1, SYK, PARP1, PTPN11, NR3C1, RAB11A, AR, RBP4, TTR, ALB, MDM2, AKT1, MAPK1 | 0.063822044 |
| GOTERM_CC_DIRECT | GO:0005884~actin filament | 5 | 0.01137431 | HCK, SRC, RAC2, TEK, RAC1 | 0.069952008 |
| GOTERM_CC_DIRECT | GO:0031258~lamellipodium membrane | 3 | 0.026481068 | DPP4, FAP, EPHA2 | 0.158886411 |
| GOTERM_CC_DIRECT | GO:0005634~nucleus | 88 | 0.028313557 | GSK3B, THRA, ADK, RORA, NR3C1, NR3C2, LGALS3, HINT1, CASP7, PNP, CASP3, AKT2, CHEK1, KDR, AKT1, CTSG, ACADM, RAC1, JAK2, PRKACA, HRAS, MAP2K1, G6PD, HSP90AA1, TPI1, PARP1, CSNK2A1, APAF1, SYK, ARG1, MMP2, AR, HCK, MTAP, BTK, RARB, ANG, PPARG, PGR, PADI4, RAF1, PPARA, S100A9, PPIA, PPARD, APCS, AHCY, SRC, GSTP1, SHMT1, NR1I2, XIAP, PIK3R1, EGFR, RXRB, SEC14L2, MAPK8, RXRA, ERBB4, CBS, ABL1, PMS2, HMOX1, MAPK1, LTA4H, HSPA8, NOS2, NOS3, STAT1, VDR, NR1H2, NR1H4, NR1H3, PTPN11, ESRRG, MAPK14, ESR1, ESR2, PTK2, FABP4, CDK6, NMNAT1, ALB, MDM2, RAN, FGFR2, CDK5R1, FGFR1 | 0.162054728 |
| GOTERM_CC_DIRECT | GO:0005578~proteinaceous extracellular matrix | 9 | 0.028813562 | MMP12, SERPINA1, MMP13, MMP7, MMP1, MMP2, MMP3, MMP8, MMP9 | 0.162054728 |
| GOTERM_CC_DIRECT | GO:0005856~cytoskeleton | 11 | 0.028985399 | HCK, HINT1, PNP, NOS3, MAPK1, TEK, JAK2, JAK3, S100A9, PTK2, RHOA | 0.162054728 |
| GOTERM_CC_DIRECT | GO:0005913~cell-cell adherens junction | 10 | 0.031073376 | HSPA8, PTPN1, STAT1, SRC, CTNNA1, EGFR, HSPA1B, RAN, EPHA2, HSPA1A | 0.169867791 |
| GOTERM_CC_DIRECT | GO:0005769~early endosome | 8 | 0.035561296 | EEA1, PTPN1, MAP2K1, AKT2, APOA2, KDR, MAPK1, RAB5A | 0.190175624 |
| GOTERM_CC_DIRECT | GO:0014069~postsynaptic density | 7 | 0.038208736 | GSK3B, ARF1, PDPK1, SRC, NCS1, PDE4B, CDK5R1 | 0.198830037 |
| GOTERM_CC_DIRECT | GO:0016023~cytoplasmic, membrane-bounded vesicle | 6 | 0.038796105 | PDPK1, KDR, ANG, RAC1, FGFR2, FGFR1 | 0.198830037 |
| GOTERM_CC_DIRECT | GO:0031410~cytoplasmic vesicle | 8 | 0.039997613 | BACE1, EEA1, PTPN1, BTK, ANG, FGFR2, RAB11A, FGFR1 | 0.200804344 |
| GOTERM_CC_DIRECT | GO:0005623~cell | 5 | 0.045991456 | LCN2, GCK, TGFBR1, IL2, HK1 | 0.226277966 |
