# Supplementary material for: Total Flavonoids of Chuju Decrease Oxidative Stress and Cell Apoptosis in Ischemic Stroke Rats: Network and Experimental Analyses
Source: Front Neurosci. 2021 Dec 9;15:772401. doi: 10.3389/fnins.2021.772401 (PMC8695723; doi:10.3389/fnins.2021.772401)
Supplement: Supplementary file 6 [file Table_5.docx]

Supplementary Table 5 Functions of potential target genes based on KEGG analysis.

| Category | Term | Count | PValue | Genes | FDR |
| --- | --- | --- | --- | --- | --- |
| KEGG_PATHWAY | hsa05200:Pathways in cancer | 48 | 1.73E-16 | GSK3B, GSTP1, XIAP, PIK3R1, EGFR, PIK3CG, IGF1R, CDC42, RXRB, MAPK8, RXRA, CASP3, AKT2, CTNNA1, ABL1, RAC2, AKT1, MAPK1, RAC1, PRKACA, HRAS, TGFB2, MAP2K1, HSP90AA1, NOS2, DAPK1, MMP1, STAT1, MMP2, IGF1, MMP9, RHOA, TGFBR1, PTK2, TGFBR2, MAPK10, AR, CDK6, KIT, MDM2, RARB, PPARG, RAF1, MET, FGFR2, FGFR1, BCL2L1, PPARD | 2.64E-14 |
| KEGG_PATHWAY | hsa05205:Proteoglycans in cancer | 31 | 3.04E-13 | SRC, PIK3R1, EGFR, PIK3CG, IGF1R, CDC42, PLAU, ERBB4, CASP3, AKT2, KDR, AKT1, MAPK1, RAC1, PRKACA, HRAS, TGFB2, MAP2K1, PDPK1, MMP2, PTPN11, IGF1, MAPK14, MMP9, ESR1, RHOA, PTK2, MDM2, RAF1, MET, FGFR1 | 2.31E-11 |
| KEGG_PATHWAY | hsa05212:Pancreatic cancer | 19 | 4.61E-13 | TGFB2, MAP2K1, STAT1, PIK3R1, EGFR, TGFBR1, PIK3CG, TGFBR2, MAPK10, CDC42, MAPK8, CDK6, AKT2, RAC2, AKT1, MAPK1, RAC1, RAF1, BCL2L1 | 2.34E-11 |
| KEGG_PATHWAY | hsa04014:Ras signaling pathway | 32 | 1.38E-12 | PIK3R1, EGFR, PIK3CG, IGF1R, CDC42, MAPK8, AKT2, KDR, ABL1, RAC2, AKT1, MAPK1, RAC1, PRKACA, HRAS, MAP2K1, INSR, PLA2G2A, PTPN11, IGF1, RHOA, MAPK10, ZAP70, KIT, TEK, RAF1, MET, RAB5A, FGFR2, FGFR1, BCL2L1, EPHA2 | 5.26E-11 |
| KEGG_PATHWAY | hsa04917:Prolactin signaling pathway | 18 | 2.76E-11 | GSK3B, MAP2K1, STAT1, SRC, PIK3R1, MAPK14, ESR1, PIK3CG, ESR2, GCK, MAPK10, MAPK8, AKT2, AKT1, MAPK1, JAK2, RAF1, HRAS | 8.00E-10 |
| KEGG_PATHWAY | hsa05210:Colorectal cancer | 17 | 3.16E-11 | GSK3B, TGFB2, MAP2K1, PIK3R1, RHOA, TGFBR1, PIK3CG, TGFBR2, MAPK10, MAPK8, CASP3, AKT2, RAC2, AKT1, MAPK1, RAC1, RAF1 | 8.00E-10 |
| KEGG_PATHWAY | hsa04915:Estrogen signaling pathway | 20 | 1.15E-10 | HSPA8, MAP2K1, HSP90AA1, NOS3, SRC, MMP2, PIK3R1, ESR1, MMP9, EGFR, PIK3CG, ESR2, AKT2, AKT1, MAPK1, RAF1, PRKACA, HRAS, HSPA1B, HSPA1A | 2.49E-09 |
| KEGG_PATHWAY | hsa04370:VEGF signaling pathway | 16 | 2.82E-10 | MAP2K1, NOS3, SRC, PIK3R1, MAPK14, PIK3CG, PTK2, CDC42, AKT2, KDR, RAC2, AKT1, MAPK1, RAC1, RAF1, HRAS | 5.36E-09 |
| KEGG_PATHWAY | hsa05230:Central carbon metabolism in cancer | 16 | 5.88E-10 | MAP2K1, G6PD, PIK3R1, EGFR, PIK3CG, GCK, HK1, AKT2, KIT, AKT1, MAPK1, RAF1, HRAS, MET, FGFR2, FGFR1 | 9.29E-09 |
| KEGG_PATHWAY | hsa04068:FoxO signaling pathway | 22 | 6.11E-10 | TGFB2, MAP2K1, PDPK1, INSR, PIK3R1, IGF1, MAPK14, SOD2, EGFR, TGFBR1, PIK3CG, TGFBR2, IGF1R, MAPK10, MAPK8, AKT2, MDM2, AKT1, MAPK1, RAF1, PCK1, HRAS | 9.29E-09 |
| KEGG_PATHWAY | hsa05215:Prostate cancer | 18 | 1.03E-09 | GSK3B, MAP2K1, HSP90AA1, PDPK1, PIK3R1, IGF1, EGFR, PIK3CG, IGF1R, AR, AKT2, MDM2, AKT1, MAPK1, RAF1, HRAS, FGFR2, FGFR1 | 1.42E-08 |
| KEGG_PATHWAY | hsa04151:PI3K-Akt signaling pathway | 35 | 1.15E-09 | GSK3B, PIK3R1, EGFR, PIK3CG, IGF1R, RXRA, AKT2, KDR, AKT1, MAPK1, RAC1, JAK2, PCK1, HRAS, JAK3, MAP2K1, HSP90AA1, SYK, NOS3, PDPK1, INSR, IGF1, IL2, PTK2, CDK6, RHEB, KIT, MDM2, TEK, RAF1, MET, FGFR2, FGFR1, BCL2L1, EPHA2 | 1.46E-08 |
| KEGG_PATHWAY | hsa04664:Fc epsilon RI signaling pathway | 16 | 1.47E-09 | MAP2K1, SYK, PDPK1, PIK3R1, MAPK14, PIK3CG, MAPK10, MAPK8, AKT2, BTK, RAC2, AKT1, MAPK1, RAC1, RAF1, HRAS | 1.71E-08 |
| KEGG_PATHWAY | hsa04520:Adherens junction | 16 | 2.78E-09 | PTPN1, CSNK2A1, SRC, INSR, RHOA, EGFR, TGFBR1, TGFBR2, IGF1R, CDC42, CTNNA1, RAC2, MAPK1, RAC1, MET, FGFR1 | 3.02E-08 |
| KEGG_PATHWAY | hsa05220:Chronic myeloid leukemia | 16 | 3.42E-09 | TGFB2, MAP2K1, PTPN11, PIK3R1, TGFBR1, PIK3CG, TGFBR2, CDK6, AKT2, MDM2, ABL1, AKT1, MAPK1, RAF1, HRAS, BCL2L1 | 3.47E-08 |
| KEGG_PATHWAY | hsa04910:Insulin signaling pathway | 21 | 6.53E-09 | PTPN1, GSK3B, MAP2K1, PKLR, PDPK1, INSR, PDE3B, PIK3R1, PIK3CG, GCK, HK1, MAPK10, MAPK8, RHEB, AKT2, AKT1, MAPK1, RAF1, PCK1, PRKACA, HRAS | 6.20E-08 |
| KEGG_PATHWAY | hsa05223:Non-small cell lung cancer | 14 | 9.73E-09 | MAP2K1, PDPK1, PIK3R1, EGFR, PIK3CG, RXRB, RXRA, CDK6, AKT2, AKT1, MAPK1, RARB, RAF1, HRAS | 8.70E-08 |
| KEGG_PATHWAY | hsa03320:PPAR signaling pathway | 15 | 1.12E-08 | PDPK1, MMP1, APOA2, NR1H3, RXRB, FABP3, RXRA, FABP4, FABP5, FABP7, PPARG, ACADM, PCK1, PPARA, PPARD | 8.93E-08 |
| KEGG_PATHWAY | hsa04919:Thyroid hormone signaling pathway | 19 | 1.12E-08 | GSK3B, MAP2K1, THRA, PDPK1, STAT1, SRC, PIK3R1, ESR1, PIK3CG, RXRB, RXRA, RHEB, AKT2, MDM2, AKT1, MAPK1, RAF1, PRKACA, HRAS | 8.93E-08 |
| KEGG_PATHWAY | hsa04015:Rap1 signaling pathway | 25 | 2.37E-08 | SRC, PIK3R1, EGFR, PIK3CG, IGF1R, CDC42, AKT2, KDR, RAC2, AKT1, MAPK1, RAC1, HRAS, MAP2K1, INSR, IGF1, MAPK14, RHOA, KIT, TEK, RAF1, MET, FGFR2, FGFR1, EPHA2 | 1.78E-07 |
| KEGG_PATHWAY | hsa05218:Melanoma | 15 | 2.46E-08 | MAP2K1, PIK3R1, IGF1, EGFR, PIK3CG, IGF1R, CDK6, AKT2, MDM2, AKT1, MAPK1, RAF1, HRAS, MET, FGFR1 | 1.78E-07 |
| KEGG_PATHWAY | hsa05219:Bladder cancer | 12 | 2.71E-08 | MAP2K1, MMP1, SRC, DAPK1, MMP2, MDM2, MAPK1, RAF1, HRAS, MMP9, EGFR, TYMP | 1.87E-07 |
| KEGG_PATHWAY | hsa04914:Progesterone-mediated oocyte maturation | 16 | 5.16E-08 | MAP2K1, HSP90AA1, PDE3B, PIK3R1, IGF1, MAPK14, PIK3CG, IGF1R, MAPK10, MAPK8, AKT2, AKT1, MAPK1, PGR, RAF1, PRKACA | 3.27E-07 |
| KEGG_PATHWAY | hsa04012:ErbB signaling pathway | 16 | 5.16E-08 | GSK3B, MAP2K1, SRC, PIK3R1, EGFR, PIK3CG, PTK2, MAPK10, MAPK8, ERBB4, AKT2, ABL1, AKT1, MAPK1, RAF1, HRAS | 3.27E-07 |
| KEGG_PATHWAY | hsa04510:Focal adhesion | 24 | 7.34E-08 | GSK3B, MAP2K1, PDPK1, SRC, XIAP, PIK3R1, IGF1, RHOA, EGFR, PIK3CG, PTK2, IGF1R, MAPK10, CDC42, MAPK8, AKT2, KDR, RAC2, AKT1, MAPK1, RAC1, RAF1, HRAS, MET | 4.46E-07 |
| KEGG_PATHWAY | hsa05152:Tuberculosis | 22 | 9.89E-08 | TGFB2, SYK, NOS2, APAF1, STAT1, SRC, VDR, MAPK14, RHOA, CTSS, MAPK10, EEA1, MAPK8, CD209, CASP3, AKT2, AKT1, MAPK1, JAK2, RAF1, CTSD, RAB5A | 5.78E-07 |
| KEGG_PATHWAY | hsa04722:Neurotrophin signaling pathway | 18 | 1.32E-07 | GSK3B, MAP2K1, PDPK1, PTPN11, PIK3R1, MAPK14, RHOA, PIK3CG, MAPK10, CDC42, MAPK8, AKT2, ABL1, AKT1, MAPK1, RAC1, RAF1, HRAS | 7.44E-07 |
| KEGG_PATHWAY | hsa05145:Toxoplasmosis | 17 | 2.16E-07 | HSPA8, TGFB2, NOS2, PDPK1, STAT1, XIAP, MAPK14, MAPK10, MAPK8, CASP3, AKT2, AKT1, MAPK1, JAK2, HSPA1B, HSPA1A, BCL2L1 | 1.17E-06 |
| KEGG_PATHWAY | hsa04660:T cell receptor signaling pathway | 16 | 3.48E-07 | GSK3B, MAP2K1, PDPK1, PIK3R1, MAPK14, RHOA, IL2, PIK3CG, CDC42, ZAP70, LCK, AKT2, AKT1, MAPK1, RAF1, HRAS | 1.82E-06 |
| KEGG_PATHWAY | hsa05213:Endometrial cancer | 12 | 3.84E-07 | GSK3B, MAP2K1, PDPK1, AKT2, CTNNA1, MAPK1, AKT1, PIK3R1, RAF1, HRAS, EGFR, PIK3CG | 1.94E-06 |
| KEGG_PATHWAY | hsa04380:Osteoclast differentiation | 18 | 4.82E-07 | TGFB2, MAP2K1, SYK, STAT1, PIK3R1, MAPK14, TGFBR1, PIK3CG, TGFBR2, MAPK10, MAPK8, LCK, AKT2, BTK, AKT1, MAPK1, PPARG, RAC1 | 2.36E-06 |
| KEGG_PATHWAY | hsa05214:Glioma | 13 | 5.40E-07 | MAP2K1, PIK3R1, IGF1, EGFR, PIK3CG, IGF1R, CDK6, AKT2, MDM2, AKT1, MAPK1, RAF1, HRAS | 2.57E-06 |
| KEGG_PATHWAY | hsa05211:Renal cell carcinoma | 13 | 6.43E-07 | TGFB2, MAP2K1, PTPN11, PIK3R1, PIK3CG, CDC42, AKT2, AKT1, MAPK1, RAC1, RAF1, HRAS, MET | 2.96E-06 |
| KEGG_PATHWAY | hsa04071:Sphingolipid signaling pathway | 17 | 7.30E-07 | MAP2K1, NOS3, PDPK1, PIK3R1, MAPK14, RHOA, PIK3CG, MAPK10, MAPK8, AKT2, RAC2, AKT1, MAPK1, RAC1, RAF1, HRAS, CTSD | 3.26E-06 |
| KEGG_PATHWAY | hsa05120:Epithelial cell signaling in Helicobacter pylori infection | 13 | 7.62E-07 | SRC, PTPN11, MAPK14, EGFR, MAPK10, CDC42, ADAM17, MAPK8, CASP3, CCL5, CSK, RAC1, MET | 3.31E-06 |
| KEGG_PATHWAY | hsa04931:Insulin resistance | 16 | 9.65E-07 | PTPN1, GSK3B, NOS3, PDPK1, NR1H2, INSR, NR1H3, PTPN11, PIK3R1, PIK3CG, MAPK10, MAPK8, AKT2, AKT1, PCK1, PPARA | 4.07E-06 |
| KEGG_PATHWAY | hsa04062:Chemokine signaling pathway | 21 | 1.01E-06 | GSK3B, MAP2K1, STAT1, SRC, PIK3R1, RHOA, PIK3CG, PTK2, CDC42, HCK, CCL5, AKT2, RAC2, AKT1, MAPK1, RAC1, JAK2, RAF1, PRKACA, HRAS, JAK3 | 4.15E-06 |
| KEGG_PATHWAY | hsa04662:B cell receptor signaling pathway | 13 | 1.06E-06 | GSK3B, MAP2K1, SYK, PIK3R1, PIK3CG, AKT2, BTK, RAC2, AKT1, MAPK1, RAC1, RAF1, HRAS | 4.24E-06 |
| KEGG_PATHWAY | hsa04066:HIF-1 signaling pathway | 15 | 1.23E-06 | MAP2K1, NOS2, NOS3, INSR, PIK3R1, IGF1, EGFR, PIK3CG, IGF1R, HK1, AKT2, AKT1, HMOX1, MAPK1, TEK | 4.80E-06 |
| KEGG_PATHWAY | hsa05161:Hepatitis B | 18 | 2.05E-06 | TGFB2, MAP2K1, APAF1, STAT1, SRC, PIK3R1, MMP9, TGFBR1, PIK3CG, MAPK10, MAPK8, CDK6, CASP3, AKT2, AKT1, MAPK1, RAF1, HRAS | 7.81E-06 |
| KEGG_PATHWAY | hsa05231:Choline metabolism in cancer | 15 | 2.30E-06 | MAP2K1, PDPK1, PIK3R1, EGFR, PIK3CG, MAPK10, MAPK8, RHEB, AKT2, RAC2, AKT1, MAPK1, RAC1, RAF1, HRAS | 8.53E-06 |
| KEGG_PATHWAY | hsa04010:MAPK signaling pathway | 24 | 2.93E-06 | HSPA8, TGFB2, MAP2K1, MAPK14, EGFR, TGFBR1, DUSP6, TGFBR2, MAPK10, CDC42, MAPK8, CASP3, AKT2, RAC2, AKT1, MAPK1, RAC1, RAF1, PRKACA, HRAS, HSPA1B, FGFR2, HSPA1A, FGFR1 | 1.04E-05 |
| KEGG_PATHWAY | hsa05160:Hepatitis C | 17 | 2.95E-06 | GSK3B, PDPK1, STAT1, NR1H3, PIK3R1, MAPK14, EGFR, PIK3CG, MAPK10, MAPK8, RXRA, AKT2, AKT1, MAPK1, RAF1, PPARA, HRAS | 1.04E-05 |
| KEGG_PATHWAY | hsa05142:Chagas disease (American trypanosomiasis) | 15 | 3.29E-06 | TGFB2, ACE, NOS2, PIK3R1, MAPK14, TGFBR1, IL2, PIK3CG, TGFBR2, MAPK10, MAPK8, CCL5, AKT2, AKT1, MAPK1 | 1.14E-05 |
| KEGG_PATHWAY | hsa04668:TNF signaling pathway | 15 | 4.63E-06 | MAP2K1, MMP3, PIK3R1, MAPK14, SELE, MMP9, PIK3CG, MAPK10, CASP7, MAPK8, CASP3, CCL5, AKT2, AKT1, MAPK1 | 1.56E-05 |
| KEGG_PATHWAY | hsa05222:Small cell lung cancer | 13 | 1.02E-05 | NOS2, APAF1, XIAP, PIK3R1, PIK3CG, PTK2, RXRB, RXRA, CDK6, AKT2, AKT1, RARB, BCL2L1 | 3.38E-05 |
| KEGG_PATHWAY | hsa05164:Influenza A | 18 | 2.43E-05 | HSPA8, GSK3B, MAP2K1, STAT1, PIK3R1, MAPK14, PIK3CG, MAPK10, MAPK8, CCL5, AKT2, CASP1, AKT1, MAPK1, JAK2, RAF1, HSPA1B, HSPA1A | 7.87E-05 |
| KEGG_PATHWAY | hsa04550:Signaling pathways regulating pluripotency of stem cells | 16 | 2.51E-05 | GSK3B, MAP2K1, PIK3R1, IGF1, MAPK14, PIK3CG, IGF1R, AKT2, AKT1, MAPK1, JAK2, RAF1, HRAS, JAK3, FGFR2, FGFR1 | 7.93E-05 |
| KEGG_PATHWAY | hsa01130:Biosynthesis of antibiotics | 20 | 2.74E-05 | GPI, GCDH, ARG2, G6PD, TPI1, PKLR, ARG1, SHMT1, HMGCR, ADH5, GCK, ACAT1, HK1, ALDH2, CBS, ACADM, PCK1, HADH, AGXT, OTC | 8.49E-05 |
| KEGG_PATHWAY | hsa04610:Complement and coagulation cascades | 11 | 4.51E-05 | CFD, F7, SERPINA1, C1S, F10, PLAU, C1R, F11, FGG, F2, CFB | 1.37E-04 |
| KEGG_PATHWAY | hsa05221:Acute myeloid leukemia | 10 | 4.72E-05 | MAP2K1, AKT2, KIT, MAPK1, AKT1, PIK3R1, RAF1, HRAS, PIK3CG, PPARD | 1.41E-04 |
| KEGG_PATHWAY | hsa04666:Fc gamma R-mediated phagocytosis | 12 | 4.94E-05 | CDC42, HCK, MAP2K1, SYK, AKT2, RAC2, MAPK1, AKT1, RAC1, PIK3R1, RAF1, PIK3CG | 1.44E-04 |
| KEGG_PATHWAY | hsa05162:Measles | 15 | 5.72E-05 | HSPA8, GSK3B, CSNK2A1, STAT1, PIK3R1, IL2, PIK3CG, CDK6, CD209, AKT2, AKT1, JAK2, JAK3, HSPA1B, HSPA1A | 1.64E-04 |
| KEGG_PATHWAY | hsa04932:Non-alcoholic fatty liver disease (NAFLD) | 16 | 6.11E-05 | GSK3B, PKLR, INSR, NR1H3, PIK3R1, PIK3CG, MAPK10, CDC42, CASP7, MAPK8, RXRA, CASP3, AKT2, AKT1, RAC1, PPARA | 1.72E-04 |
| KEGG_PATHWAY | hsa04650:Natural killer cell mediated cytotoxicity | 14 | 9.30E-05 | MAP2K1, SYK, ICAM2, PTPN11, PIK3R1, PIK3CG, ZAP70, LCK, CASP3, RAC2, MAPK1, RAC1, RAF1, HRAS | 2.54E-04 |
| KEGG_PATHWAY | hsa05133:Pertussis | 11 | 9.35E-05 | MAPK10, CASP7, MAPK8, NOS2, C1S, C1R, CASP3, CASP1, MAPK1, MAPK14, RHOA | 2.54E-04 |
| KEGG_PATHWAY | hsa04930:Type II diabetes mellitus | 9 | 9.61E-05 | MAPK10, MAPK8, PKLR, INSR, MAPK1, PIK3R1, GCK, PIK3CG, HK1 | 2.56E-04 |
| KEGG_PATHWAY | hsa04912:GnRH signaling pathway | 12 | 1.04E-04 | MAPK10, CDC42, MAP2K1, MAPK8, SRC, MMP2, MAPK1, RAF1, MAPK14, PRKACA, HRAS, EGFR | 2.73E-04 |
| KEGG_PATHWAY | hsa04611:Platelet activation | 14 | 1.78E-04 | SYK, NOS3, SRC, FGG, GP1BA, PIK3R1, MAPK14, RHOA, PIK3CG, AKT2, BTK, AKT1, MAPK1, PRKACA | 4.59E-04 |
| KEGG_PATHWAY | hsa04920:Adipocytokine signaling pathway | 10 | 2.79E-04 | RXRB, MAPK10, MAPK8, RXRA, AKT2, AKT1, PTPN11, PCK1, JAK2, PPARA | 7.06E-04 |
| KEGG_PATHWAY | hsa04024:cAMP signaling pathway | 17 | 3.96E-04 | MAP2K1, PDE4D, PDE3B, PIK3R1, RHOA, PIK3CG, MAPK10, MAPK8, AKT2, PDE4B, RAC2, AKT1, MAPK1, RAC1, RAF1, PPARA, PRKACA | 9.88E-04 |
| KEGG_PATHWAY | hsa04620:Toll-like receptor signaling pathway | 12 | 4.07E-04 | MAPK10, MAP2K1, MAPK8, STAT1, CCL5, AKT2, MAPK1, AKT1, RAC1, PIK3R1, MAPK14, PIK3CG | 9.99E-04 |
| KEGG_PATHWAY | hsa04210:Apoptosis | 9 | 5.94E-04 | CASP7, APAF1, AKT2, CASP3, XIAP, AKT1, PIK3R1, PIK3CG, BCL2L1 | 0.001432596 |
| KEGG_PATHWAY | hsa00330:Arginine and proline metabolism | 8 | 7.96E-04 | ARG2, MAOB, ALDH2, NOS2, NOS3, ARG1, AMD1, SRM | 0.001889448 |
| KEGG_PATHWAY | hsa04670:Leukocyte transendothelial migration | 12 | 8.17E-04 | CDC42, MMP2, CTNNA1, RAC2, PTPN11, RAC1, PIK3R1, MAPK14, MMP9, PTK2, RHOA, PIK3CG | 0.001911534 |
| KEGG_PATHWAY | hsa00010:Glycolysis / Gluconeogenesis | 9 | 0.001003383 | GPI, TPI1, PKLR, ALDH2, ADH1C, PCK1, ADH5, GCK, HK1 | 0.002310821 |
| KEGG_PATHWAY | hsa04960:Aldosterone-regulated sodium reabsorption | 7 | 0.001139554 | PDPK1, INSR, MAPK1, IGF1, PIK3R1, PIK3CG, NR3C2 | 0.002585256 |
| KEGG_PATHWAY | hsa05134:Legionellosis | 8 | 0.001270431 | HSPA8, ARF1, CASP7, APAF1, CASP3, CASP1, HSPA1B, HSPA1A | 0.002839786 |
| KEGG_PATHWAY | hsa04152:AMPK signaling pathway | 12 | 0.00142656 | PDPK1, RHEB, AKT2, INSR, AKT1, PPARG, HMGCR, IGF1, PIK3R1, PCK1, PIK3CG, IGF1R | 0.003142567 |
| KEGG_PATHWAY | hsa05146:Amoebiasis | 11 | 0.001560349 | ARG2, TGFB2, NOS2, ARG1, CASP3, CTSG, PIK3R1, PRKACA, RAB5A, PTK2, PIK3CG | 0.003380316 |
| KEGG_PATHWAY | hsa04923:Regulation of lipolysis in adipocytes | 8 | 0.001578963 | FABP4, AKT2, INSR, PDE3B, AKT1, PIK3R1, PRKACA, PIK3CG | 0.003380316 |
| KEGG_PATHWAY | hsa00071:Fatty acid degradation | 7 | 0.001696149 | GCDH, ALDH2, ADH1C, ACADM, HADH, ADH5, ACAT1 | 0.003580758 |
| KEGG_PATHWAY | hsa05216:Thyroid cancer | 6 | 0.001757235 | RXRB, MAP2K1, RXRA, MAPK1, PPARG, HRAS | 0.003658901 |
| KEGG_PATHWAY | hsa04360:Axon guidance | 12 | 0.001848446 | CDC42, GSK3B, RAC2, ABL1, MAPK1, RAC1, HRAS, MET, EPHB4, PTK2, RHOA, EPHA2 | 0.003796807 |
| KEGG_PATHWAY | hsa05202:Transcriptional misregulation in cancer | 14 | 0.001942669 | MMP3, IGF1, MMP9, PTK2, TGFBR2, IGF1R, RXRB, RXRA, PLAU, MDM2, PPARG, MET, ELANE, BCL2L1 | 0.003885904 |
| KEGG_PATHWAY | hsa04150:mTOR signaling pathway | 8 | 0.001942952 | PDPK1, RHEB, AKT2, MAPK1, AKT1, IGF1, PIK3R1, PIK3CG | 0.003885904 |
| KEGG_PATHWAY | hsa04810:Regulation of actin cytoskeleton | 16 | 0.002106104 | MAP2K1, SRC, PIK3R1, F2, RHOA, EGFR, PIK3CG, PTK2, CDC42, RAC2, MAPK1, RAC1, RAF1, HRAS, FGFR2, FGFR1 | 0.004157503 |
| KEGG_PATHWAY | hsa01200:Carbon metabolism | 11 | 0.002521048 | GPI, G6PD, TPI1, PKLR, SHMT1, ACADM, AGXT, ADH5, GCK, ACAT1, HK1 | 0.004912811 |
| KEGG_PATHWAY | hsa05100:Bacterial invasion of epithelial cells | 9 | 0.002697655 | CDC42, SRC, CTNNA1, RAC1, PIK3R1, MET, PTK2, RHOA, PIK3CG | 0.005190426 |
| KEGG_PATHWAY | hsa01100:Metabolic pathways | 55 | 0.002881091 | PNMT, GPI, AMD1, ADK, AKR1B1, HK1, NDST1, PNP, IMPA1, ALDH2, ACADM, HADH, ARG2, G6PD, TPI1, ARG1, APRT, MTAP, FOLH1, HPRT1, AGXT, CES1, GCDH, TPH1, AHCY, MAOB, FECH, ADH1C, SHMT1, GBA, HMGCR, CYP19A1, ADH5, ABO, ACAT1, SRM, TYMP, HSD11B1, CBS, PNPO, LTA4H, PCK1, PKLR, NOS2, NOS3, EPHX2, PLA2G2A, GCK, GSTZ1, DHFR, CYP2C9, BHMT, NMNAT1, PAH, OTC | 0.005474073 |
| KEGG_PATHWAY | hsa00220:Arginine biosynthesis | 5 | 0.002930469 | ARG2, NOS2, NOS3, ARG1, OTC | 0.005499152 |
| KEGG_PATHWAY | hsa04144:Endocytosis | 17 | 0.003172472 | HSPA8, ARF1, SRC, RHOA, EGFR, TGFBR1, RAB11A, TGFBR2, IGF1R, CDC42, EEA1, MDM2, HRAS, RAB5A, HSPA1B, FGFR2, HSPA1A | 0.005880679 |
| KEGG_PATHWAY | hsa05131:Shigellosis | 8 | 0.003433299 | MAPK10, CDC42, MAPK8, SRC, ABL1, MAPK1, RAC1, MAPK14 | 0.006287487 |
| KEGG_PATHWAY | hsa00350:Tyrosine metabolism | 6 | 0.004132566 | PNMT, GSTZ1, MAOB, ADH1C, MIF, ADH5 | 0.007477977 |
| KEGG_PATHWAY | hsa05014:Amyotrophic lateral sclerosis (ALS) | 7 | 0.004185056 | APAF1, CASP3, CASP1, RAC1, MAPK14, RAB5A, BCL2L1 | 0.007483865 |
| KEGG_PATHWAY | hsa05203:Viral carcinogenesis | 15 | 0.004418622 | SYK, SRC, PIK3R1, RHOA, PIK3CG, CDC42, CDK6, CASP3, CHEK1, MDM2, MAPK1, RAC1, PRKACA, HRAS, JAK3 | 0.007809658 |
| KEGG_PATHWAY | hsa04614:Renin-angiotensin system | 5 | 0.004980678 | ACE, MME, CMA1, REN, CTSG | 0.008701875 |
| KEGG_PATHWAY | hsa05166:HTLV-I infection | 17 | 0.005289911 | GSK3B, TGFB2, XIAP, PIK3R1, TGFBR1, IL2, PIK3CG, TGFBR2, LCK, AKT2, CHEK1, AKT1, PRKACA, HRAS, JAK3, RAN, BCL2L1 | 0.00913712 |
| KEGG_PATHWAY | hsa00270:Cysteine and methionine metabolism | 6 | 0.005928158 | BHMT, MTAP, AHCY, CBS, AMD1, SRM | 0.010124494 |
| KEGG_PATHWAY | hsa01230:Biosynthesis of amino acids | 8 | 0.006614029 | ARG2, TPI1, PKLR, CBS, ARG1, SHMT1, PAH, OTC | 0.01117036 |
| KEGG_PATHWAY | hsa04621:NOD-like receptor signaling pathway | 7 | 0.007336231 | MAPK10, HSP90AA1, MAPK8, CCL5, CASP1, MAPK1, MAPK14 | 0.012214835 |
| KEGG_PATHWAY | hsa00380:Tryptophan metabolism | 6 | 0.00739319 | GCDH, TPH1, MAOB, ALDH2, HADH, ACAT1 | 0.012214835 |
| KEGG_PATHWAY | hsa05169:Epstein-Barr virus infection | 10 | 0.013110033 | MAPK10, MAPK8, SYK, AKT2, MDM2, AKT1, PIK3R1, MAPK14, JAK3, PIK3CG | 0.02142715 |
| KEGG_PATHWAY | hsa05132:Salmonella infection | 8 | 0.014027439 | MAPK10, CDC42, MAPK8, NOS2, CASP1, MAPK1, RAC1, MAPK14 | 0.022682668 |
| KEGG_PATHWAY | hsa04064:NF-kappa B signaling pathway | 8 | 0.017809674 | ZAP70, CSNK2A1, SYK, PLAU, LCK, BTK, XIAP, BCL2L1 | 0.028495478 |
| KEGG_PATHWAY | hsa00982:Drug metabolism - cytochrome P450 | 7 | 0.018234535 | CYP2C9, GSTM1, MAOB, ADH1C, GSTO1, GSTP1, ADH5 | 0.028871348 |
| KEGG_PATHWAY | hsa00480:Glutathione metabolism | 6 | 0.020089322 | G6PD, GSTM1, GSTO1, GSTP1, GSR, SRM | 0.031480175 |
| KEGG_PATHWAY | hsa04725:Cholinergic synapse | 9 | 0.021406126 | MAP2K1, AKT2, MAPK1, AKT1, PIK3R1, JAK2, PRKACA, HRAS, PIK3CG | 0.032865971 |
| KEGG_PATHWAY | hsa04726:Serotonergic synapse | 9 | 0.021406126 | CYP2C9, MAP2K1, TPH1, MAOB, CASP3, MAPK1, RAF1, PRKACA, HRAS | 0.032865971 |
| KEGG_PATHWAY | hsa05150:Staphylococcus aureus infection | 6 | 0.025117042 | SELP, CFD, C1S, C1R, FGG, CFB | 0.038177904 |
| KEGG_PATHWAY | hsa00980:Metabolism of xenobiotics by cytochrome P450 | 7 | 0.026524423 | HSD11B1, CYP2C9, GSTM1, ADH1C, GSTO1, GSTP1, ADH5 | 0.039917943 |
| KEGG_PATHWAY | hsa04310:Wnt signaling pathway | 10 | 0.027048607 | MAPK10, GSK3B, MAPK8, MMP7, CSNK2A1, RAC2, RAC1, PRKACA, RHOA, PPARD | 0.040307728 |
| KEGG_PATHWAY | hsa04612:Antigen processing and presentation | 7 | 0.029765131 | HSPA8, HSP90AA1, TAP1, CTSS, HSPA1B, CTSB, HSPA1A | 0.043925242 |
| KEGG_PATHWAY | hsa00260:Glycine, serine and threonine metabolism | 5 | 0.031532104 | BHMT, MAOB, CBS, SHMT1, AGXT | 0.046035152 |
| KEGG_PATHWAY | hsa04750:Inflammatory mediator regulation of TRP channels | 8 | 0.031800599 | MAPK10, MAPK8, SRC, IGF1, PIK3R1, MAPK14, PRKACA, PIK3CG | 0.046035152 |
| KEGG_PATHWAY | hsa00620:Pyruvate metabolism | 5 | 0.034214205 | PKLR, ALDH2, GLO1, PCK1, ACAT1 | 0.049061879 |
| KEGG_PATHWAY | hsa04630:Jak-STAT signaling pathway | 10 | 0.035618915 | STAT1, AKT2, AKT1, PTPN11, PIK3R1, JAK2, JAK3, IL2, PIK3CG, BCL2L1 | 0.050598832 |
| KEGG_PATHWAY | hsa05204:Chemical carcinogenesis | 7 | 0.037002103 | HSD11B1, CYP2C9, GSTM1, ADH1C, GSTO1, GSTP1, ADH5 | 0.052077034 |
| KEGG_PATHWAY | hsa04730:Long-term depression | 6 | 0.037431479 | MAP2K1, MAPK1, IGF1, RAF1, HRAS, IGF1R | 0.052198026 |
| KEGG_PATHWAY | hsa04973:Carbohydrate digestion and absorption | 5 | 0.039965341 | AKT2, AKT1, PIK3R1, PIK3CG, HK1 | 0.055224835 |
