# Supplementary material for: Total Flavonoids of Chuju Decrease Oxidative Stress and Cell Apoptosis in Ischemic Stroke Rats: Network and Experimental Analyses
Source: Front Neurosci. 2021 Dec 9;15:772401. doi: 10.3389/fnins.2021.772401 (PMC8695723; doi:10.3389/fnins.2021.772401)
Supplement: Supplementary file 7 [file Table_6.docx]

Supplement Table 6 The F value and df of Figures

| Name | |  | df | F value | Figures |
| --- | --- | --- | --- | --- | --- |
|  | Neurofunctional score | Between Group | 5 | 13.338 | Fig.1 |
|  |  | Within Group | 34 |  |  |
|  |  | Total | 39 |  |  |
|  | Infarction size | Between Group | 5 | 420.673 |  |
|  |  | Within Group | 34 |  |  |
|  |  | Total | 39 |  |  |
| serum | SOD | Between Group | 5 | 15.771 | Fig.2 |
|  |  | Within Group | 34 |  |  |
|  |  | Total | 39 |  |  |
|  | GSH-Px | Between Group | 5 | 113.24 |  |
|  |  | Within Group | 34 |  |  |
|  |  | Total | 39 |  |  |
|  | CAT | Between Group | 5 | 61.851 |  |
|  |  | Within Group | 34 |  |  |
|  |  | Total | 39 |  |  |
|  | MDA | Between Group | 5 | 67.739 |  |
|  |  | Within Group | 34 |  |  |
|  |  | Total | 39 |  |  |
| brain tissue | SOD | Between Group | 5 | 19.753 |  |
|  |  | Within Group | 34 |  |  |
|  |  | Total | 39 |  |  |
|  | GSH-Px | Between Group | 5 | 55.767 |  |
|  |  | Within Group | 34 |  |  |
|  |  | Total | 39 |  |  |
|  | CAT | Between Group | 5 | 9.186 |  |
|  |  | Within Group | 34 |  |  |
|  |  | Total | 39 |  |  |
|  | MDA | Between Group | 5 | 11.321 |  |
|  |  | Within Group | 34 |  |  |
|  |  | Total | 39 |  |  |
|  | TUNEL positive cells | Between Group | 5 | 57.397 | Fig.3 |
|  |  | Within Group | 24 |  |  |
|  |  | Total | 29 |  |  |
|  | Akt | Between Group | 3 | 0.564 | Fig.5 |
|  |  | Within Group | 16 |  |  |
|  |  | Total | 19 |  |  |
|  | p-Akt | Between Group | 3 | 996.504 |  |
|  |  | Within Group | 16 |  |  |
|  |  | Total | 19 |  |  |
|  | mTOR | Between Group | 3 | 1.812 |  |
|  |  | Within Group | 16 |  |  |
|  |  | Total | 19 |  |  |
|  | p-mTOR | Between Group | 3 | 665.334 |  |
|  |  | Within Group | 16 |  |  |
|  |  | Total | 19 |  |  |
| oxidative stress and PI3K/AKT/mTOR pathway | SOD | Between Group | 3 | 21.113 | Fig.6 |
|  |  | Within Group | 16 |  |  |
|  |  | Total | 19 |  |  |
|  | GSH-Px | Between Group | 3 | 144.201 |  |
|  |  | Within Group | 16 |  |  |
|  |  | Total | 19 |  |  |
|  | CAT | Between Group | 3 | 106.823 |  |
|  |  | Within Group | 16 |  |  |
|  |  | Total | 19 |  |  |
|  | MDA | Between Group | 3 | 299.417 |  |
|  |  | Within Group | 16 |  |  |
|  |  | Total | 19 |  |  |
| WB | BCL-2 | Between Group | 3 | 1043.665 | Fig.7 |
|  |  | Within Group | 16 |  |  |
|  |  | Total | 19 |  |  |
|  | BAX | Between Group | 3 | 777.901 |  |
|  |  | Within Group | 16 |  |  |
|  |  | Total | 19 |  |  |
|  | cleaved-Caspase-3 | Between Group | 3 | 1741.512 |  |
|  |  | Within Group | 16 |  |  |
|  |  | Total | 19 |  |  |
| PCR | BCL-2 | Between Group | 3 | 22.795 |  |
|  |  | Within Group | 16 |  |  |
|  |  | Total | 19 |  |  |
|  | BAX | Between Group | 3 | 88.473 |  |
|  |  | Within Group | 16 |  |  |
|  |  | Total | 19 |  |  |
|  | cleaved-Caspase-3 | Between Group | 3 | 72.649 |  |
|  |  | Within Group | 16 |  |  |
|  |  | Total | 19 |  |  |
